# Supplementary material for: Histone Methylation by SETD1A Protects Nascent DNA through the Nucleosome Chaperone Activity of FANCD2
Source: Mol Cell. 2018 Jul 5;71(1):25–41.e6. doi: 10.1016/j.molcel.2018.05.018 (PMC6039718; doi:10.1016/j.molcel.2018.05.018)
Supplement: Document S1. Figures S1–S7 [file mmc1.pdf]

Molecular Cell, Volume *71*

Supplemental Information

Histone Methylation by SETD1A Protects Nascent DNA through the Nucleosome Chaperone Activity of FANCD2

Martin R. Higgs, Koichi Sato, John J. Reynolds, Shabana Begum, Rachel Bayley, Amalia Goula, Audrey Vernet, Karissa L. Paquin, David G. Skalnik, Wataru Kobayashi, Minoru Takata, Niall G. Howlett, Hitoshi Kurumizaka, Hiroshi Kimura, and Grant S. Stewart

Figure S1

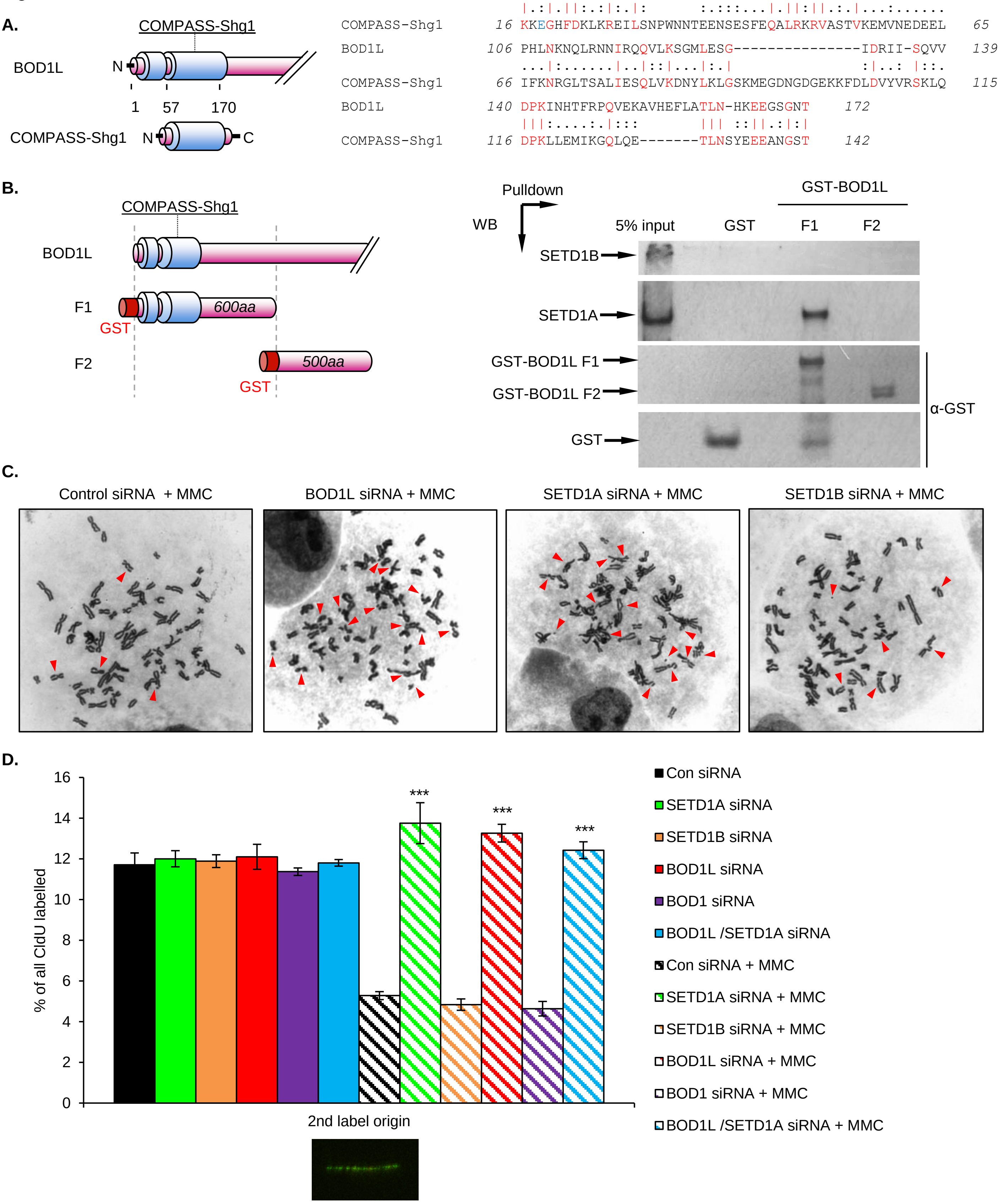
A.
COMPASS-Shg1
BOD1L
N
1
57
170
COMPASS-Shg1
N
C
BOD1L 62 KSQGLFDQFRRDCLA------DVDTKPAYQNLRQRVDNFVANHLATHTWS 105
COMPASS-Shg1 16 KKEGHFDKLKREILSNPWNNTEENSESFEQALRKRVASTVKEMVNEDEEL 65
BOD1L 106 PHLNKNQLRNNIRQQVLKSGMLESG---------------IDRII-SQVV 139
COMPASS-Shg1 66 IFKNRGLTSALIESQLVKDNYLKLGSKMEGDNGDGEKKFDLDVYVRSKLQ 115
BOD1L 140 DPKINHTFRPQVEKAVHEFLATLN-HKEEGSGNT 172
COMPASS-Shg1 116 DPKLLEMIKGQLQE-------TLNSYEEEANGST 142
B.
COMPASS-Shg1
BOD1L
F1
600aa
GST
F2
500aa
GST
Pulldown
WB
GST-BOD1L
5% input
GST
F1
F2
SETD1B
SETD1A
GST-BOD1L F1
GST-BOD1L F2
GST
α-GST
C.
Control siRNA + MMC
BOD1L siRNA + MMC
SETD1A siRNA + MMC
SETD1B siRNA + MMC
D.
% of all CldU labelled
0
2
4
6
8
10
12
14
16
***
***
***
2nd label origin
Con siRNA
SETD1A siRNA
SETD1B siRNA
BOD1L siRNA
BOD1 siRNA
BOD1L /SETD1A siRNA
Con siRNA + MMC
SETD1A siRNA + MMC
SETD1B siRNA + MMC
BOD1L siRNA + MMC
BOD1 siRNA + MMC
BOD1L /SETD1A siRNA + MMC

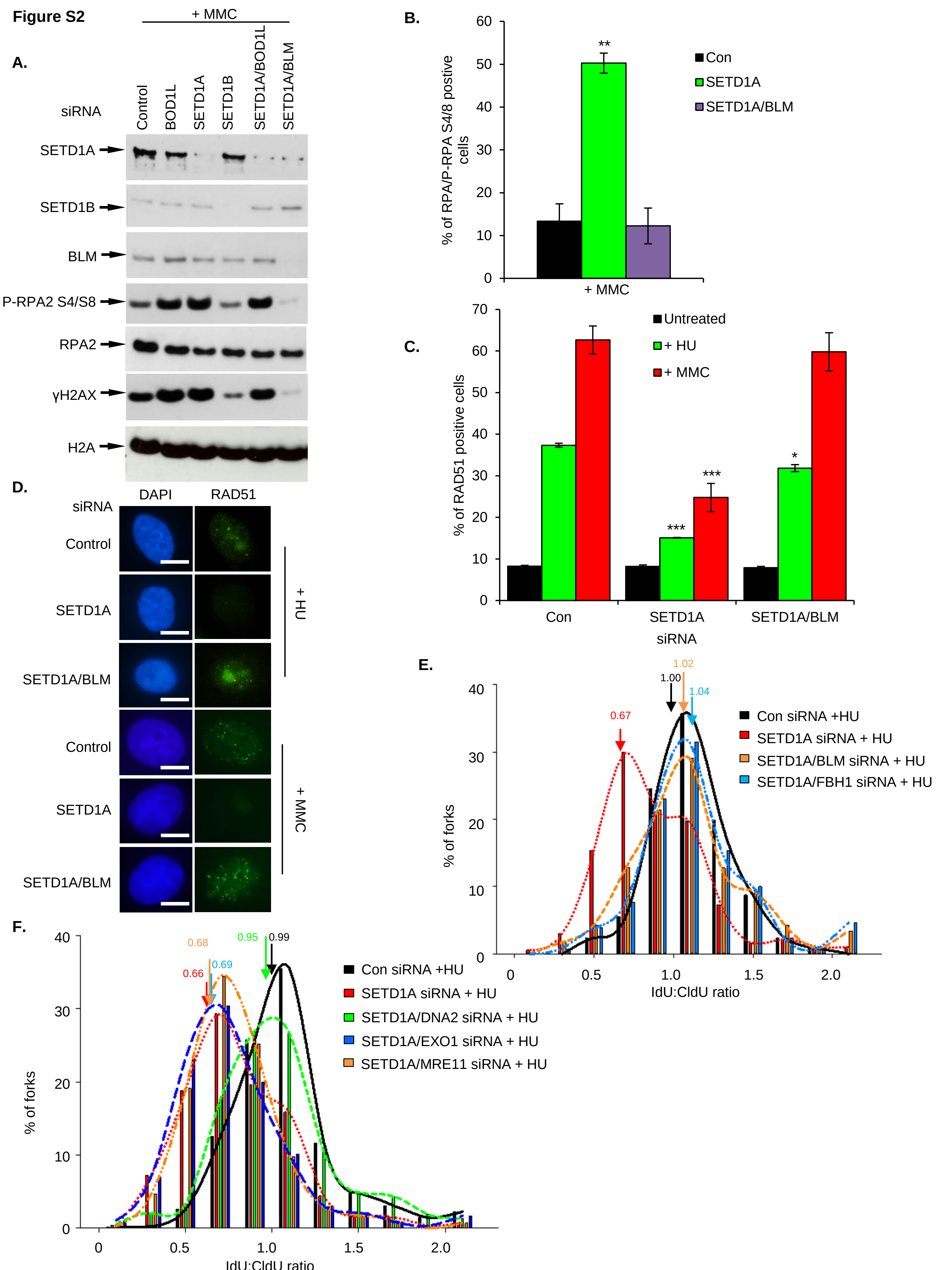

Figure S2
A.
+ MMC
siRNA
Control
BOD1L
SETD1A
SETD1B
SETD1A/BOD1L
SETD1A/BLM
SETD1A
SETD1B
BLM
P-RPA2 S4/S8
RPA2
γH2AX
H2A
B.
% of RPA/P-RPA S4/8 postive cells
Con
SETD1A
SETD1A/BLM
**
+ MMC
C.
% of RAD51 positive cells
Untreated
+ HU
+ MMC
*
***
***
Con
SETD1A
SETD1A/BLM
siRNA
D.
siRNA
DAPI
RAD51
Control
SETD1A
SETD1A/BLM
+ HU
+ MMC
E.
% of forks
IdU:CldU ratio
0.67
1.00
1.02
1.04
Con siRNA +HU
SETD1A siRNA + HU
SETD1A/BLM siRNA + HU
SETD1A/FBH1 siRNA + HU
F.
% of forks
IdU:CldU ratio
0.66
0.68
0.69
0.95
0.99
Con siRNA +HU
SETD1A siRNA + HU
SETD1A/DNA2 siRNA + HU
SETD1A/EXO1 siRNA + HU
SETD1A/MRE11 siRNA + HU

Figure S3

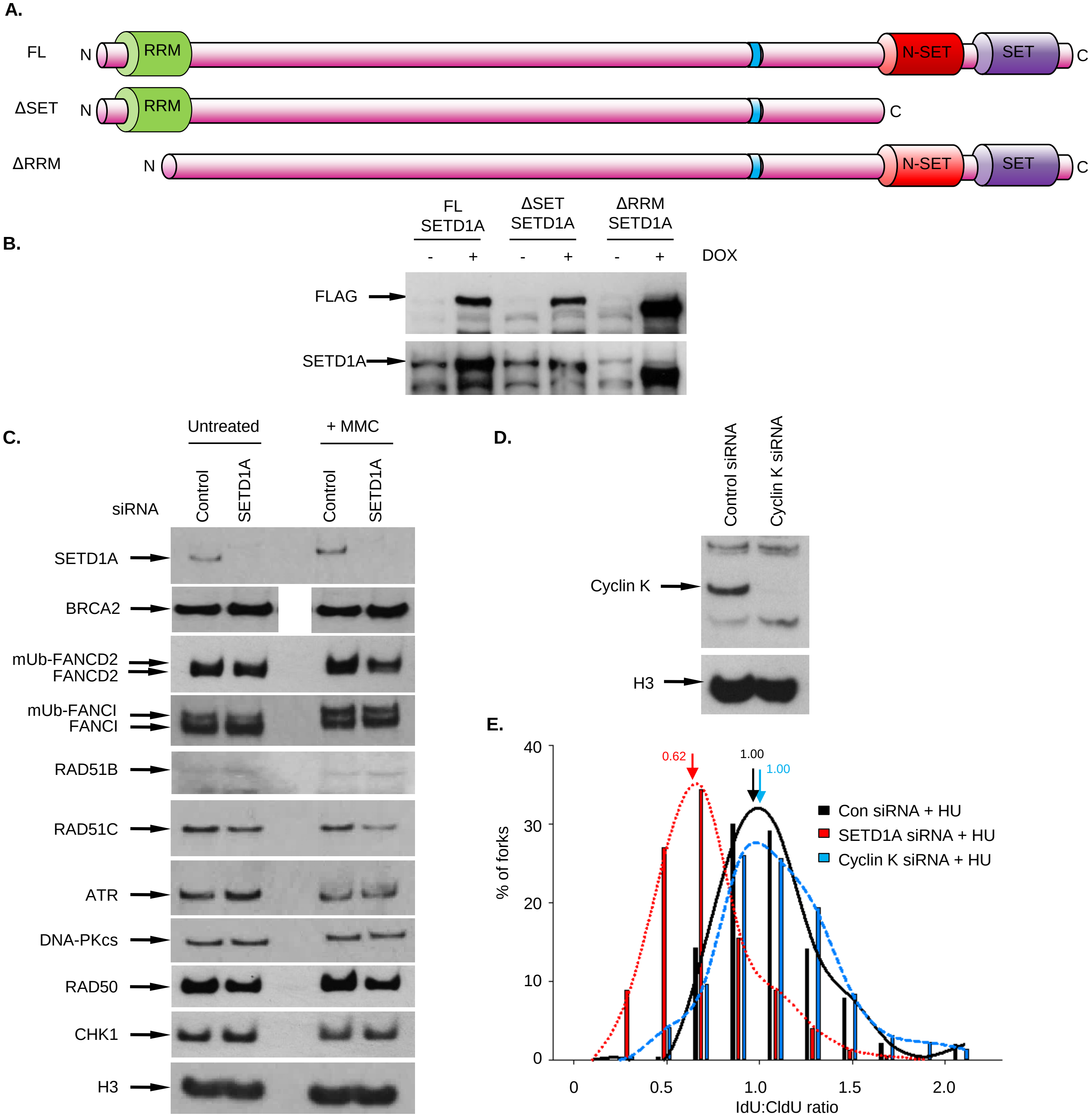
A.
FL
N
RRM
N-SET
SET
C
ΔSET
N
RRM
C
ΔRRM
N
N-SET
SET
C
B.
FL SETD1A
ΔSET SETD1A
ΔRRM SETD1A
- + - + - +
DOX
FLAG
SETD1A
C.
Untreated
+ MMC
siRNA
Control
SETD1A
Control
SETD1A
SETD1A
BRCA2
mUb-FANCD2
FANCD2
mUb-FANCI
FANCI
RAD51B
RAD51C
ATR
DNA-PKcs
RAD50
CHK1
H3
D.
Control siRNA
Cyclin K siRNA
Cyclin K
H3
E.
% of forks
40
30
20
10
0
0.62
1.00
1.00
Con siRNA + HU
SETD1A siRNA + HU
Cyclin K siRNA + HU
0
0.5
1.0
1.5
2.0
IdU:CldU ratio

Figure S4

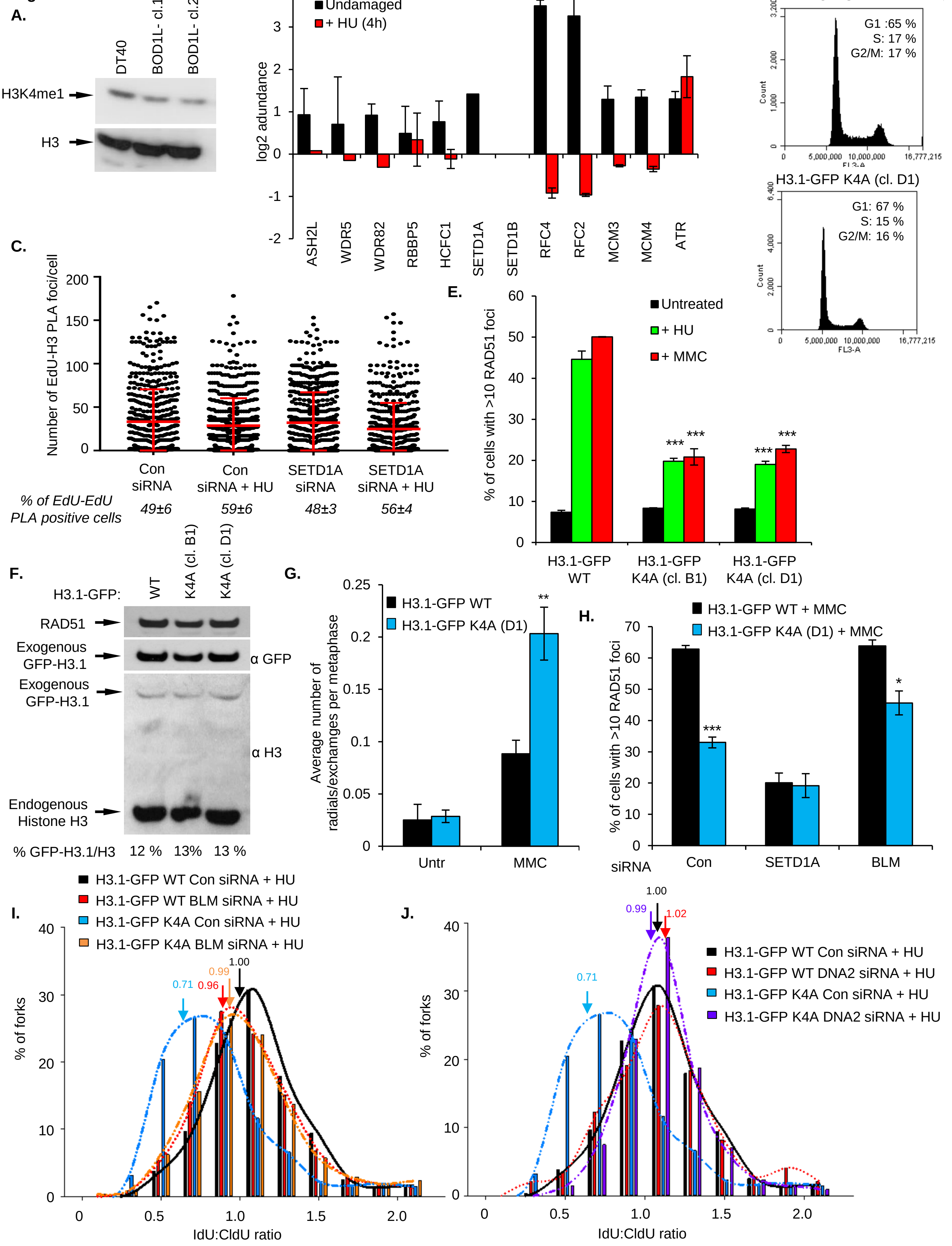
A.
DT40
BOD1L- cl.1
BOD1L- cl.2
H3K4me1
H3
B.
Undamaged
+ HU (4h)
log2 adundance
ASH2L
WDR5
WDR82
RBBP5
HCFC1
SETD1A
SETD1B
RFC4
RFC2
MCM3
MCM4
ATR
C.
Number of EdU-H3 PLA foci/cell
Con siRNA
Con siRNA + HU
SETD1A siRNA
SETD1A siRNA + HU
% of EdU-EdU PLA positive cells
49±6
59±6
48±3
56±4
D.
H3.1-GFP WT
G1 :65 %
S: 17 %
G2/M: 17 %
H3.1-GFP K4A (cl. D1)
G1: 67 %
S: 15 %
G2/M: 16 %
Count
FL3-A
E.
Untreated
+ HU
+ MMC
% of cells with >10 RAD51 foci
H3.1-GFP WT
H3.1-GFP K4A (cl. B1)
H3.1-GFP K4A (cl. D1)
F.
H3.1-GFP:
WT
K4A (cl. B1)
K4A (cl. D1)
RAD51
Exogenous GFP-H3.1
α GFP
Exogenous GFP-H3.1
α H3
Endogenous Histone H3
% GFP-H3.1/H3
12 %
13%
13 %
G.
H3.1-GFP WT
H3.1-GFP K4A (D1)
Average number of radials/exchamges per metaphase
Untr
MMC
H.
H3.1-GFP WT + MMC
H3.1-GFP K4A (D1) + MMC
% of cells with >10 RAD51 foci
siRNA
Con
SETD1A
BLM
I.
H3.1-GFP WT Con siRNA + HU
H3.1-GFP WT BLM siRNA + HU
H3.1-GFP K4A Con siRNA + HU
H3.1-GFP K4A BLM siRNA + HU
0.71
0.96
0.99
1.00
% of forks
IdU:CldU ratio
J.
H3.1-GFP WT Con siRNA + HU
H3.1-GFP WT DNA2 siRNA + HU
H3.1-GFP K4A Con siRNA + HU
H3.1-GFP K4A DNA2 siRNA + HU
0.71
0.99
1.00
1.02
% of forks
IdU:CldU ratio

Figure S5

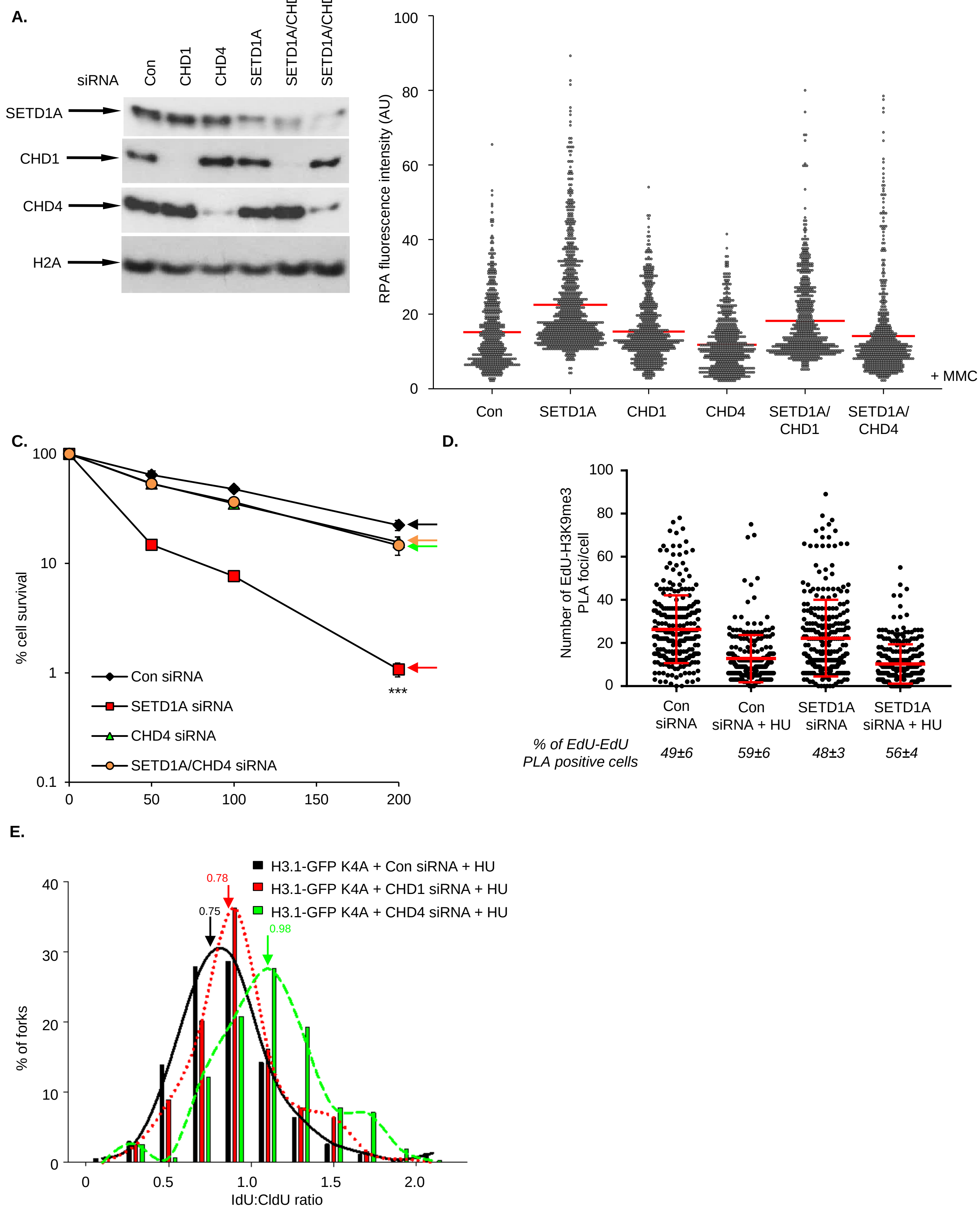

A.
siRNA
Con
CHD1
CHD4
SETD1A
SETD1A/CHD1
SETD1A/CHD4
SETD1A
CHD1
CHD4
H2A
B.
RPA fluorescence intensity (AU)
100
80
60
40
20
0
Con
SETD1A
CHD1
CHD4
SETD1A/ CHD1
SETD1A/ CHD4
+ MMC
C.
% cell survival
100
10
1
0.1
0
50
100
150
200
***
Con siRNA
SETD1A siRNA
CHD4 siRNA
SETD1A/CHD4 siRNA
D.
Number of EdU-H3K9me3 PLA foci/cell
100
80
60
40
20
0
Con siRNA
Con siRNA + HU
SETD1A siRNA
SETD1A siRNA + HU
% of EdU-EdU PLA positive cells
49±6
59±6
48±3
56±4
E.
H3.1-GFP K4A + Con siRNA + HU
H3.1-GFP K4A + CHD1 siRNA + HU
H3.1-GFP K4A + CHD4 siRNA + HU
0.78
0.75
0.98
% of forks
40
30
20
10
0
0
0.5
1.0
1.5
2.0
IdU:CldU ratio

Figure S6

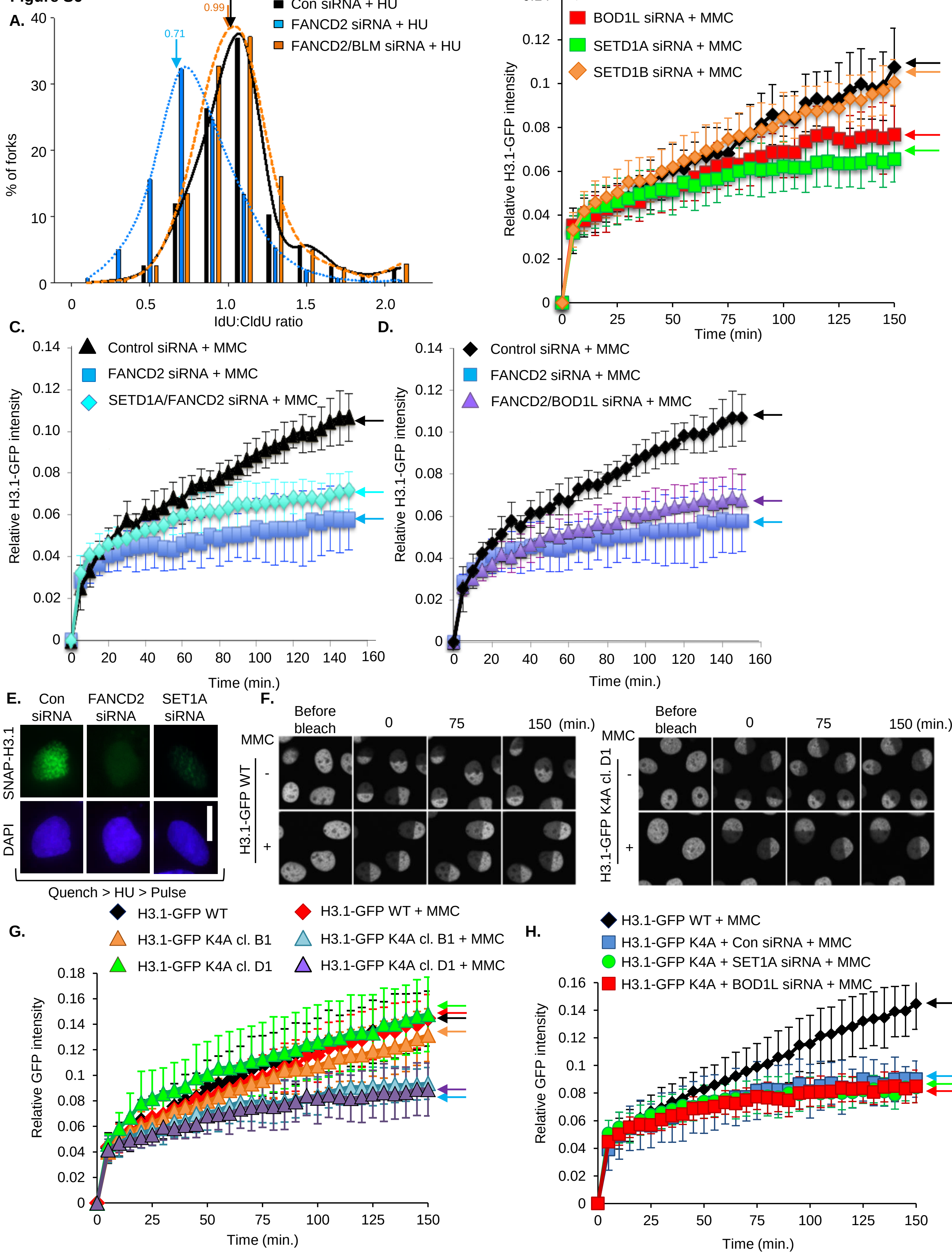

A.
0.99
0.99
0.71
Con siRNA + HU
FANCD2 siRNA + HU
FANCD2/BLM siRNA + HU
% of forks
IdU:CldU ratio
B.
Con siRNA + MMC
BOD1L siRNA + MMC
SETD1A siRNA + MMC
SETD1B siRNA + MMC
Relative H3.1-GFP intensity
Time (min)
C.
Control siRNA + MMC
FANCD2 siRNA + MMC
SETD1A/FANCD2 siRNA + MMC
Relative H3.1-GFP intensity
Time (min.)
D.
Control siRNA + MMC
FANCD2 siRNA + MMC
FANCD2/BOD1L siRNA + MMC
Relative H3.1-GFP intensity
Time (min.)
E.
Con siRNA
FANCD2 siRNA
SET1A siRNA
SNAP-H3.1
DAPI
Quench > HU > Pulse
F.
Before bleach
0
75
150 (min.)
MMC
H3.1-GFP WT
-
+
H3.1-GFP K4A cl. D1
G.
H3.1-GFP WT
H3.1-GFP K4A cl. B1
H3.1-GFP K4A cl. D1
H3.1-GFP WT + MMC
H3.1-GFP K4A cl. B1 + MMC
H3.1-GFP K4A cl. D1 + MMC
Relative GFP intensity
Time (min.)
H.
H3.1-GFP WT + MMC
H3.1-GFP K4A + Con siRNA + MMC
H3.1-GFP K4A + SET1A siRNA + MMC
H3.1-GFP K4A + BOD1L siRNA + MMC
Relative GFP intensity
Time (min.)

Figure S7

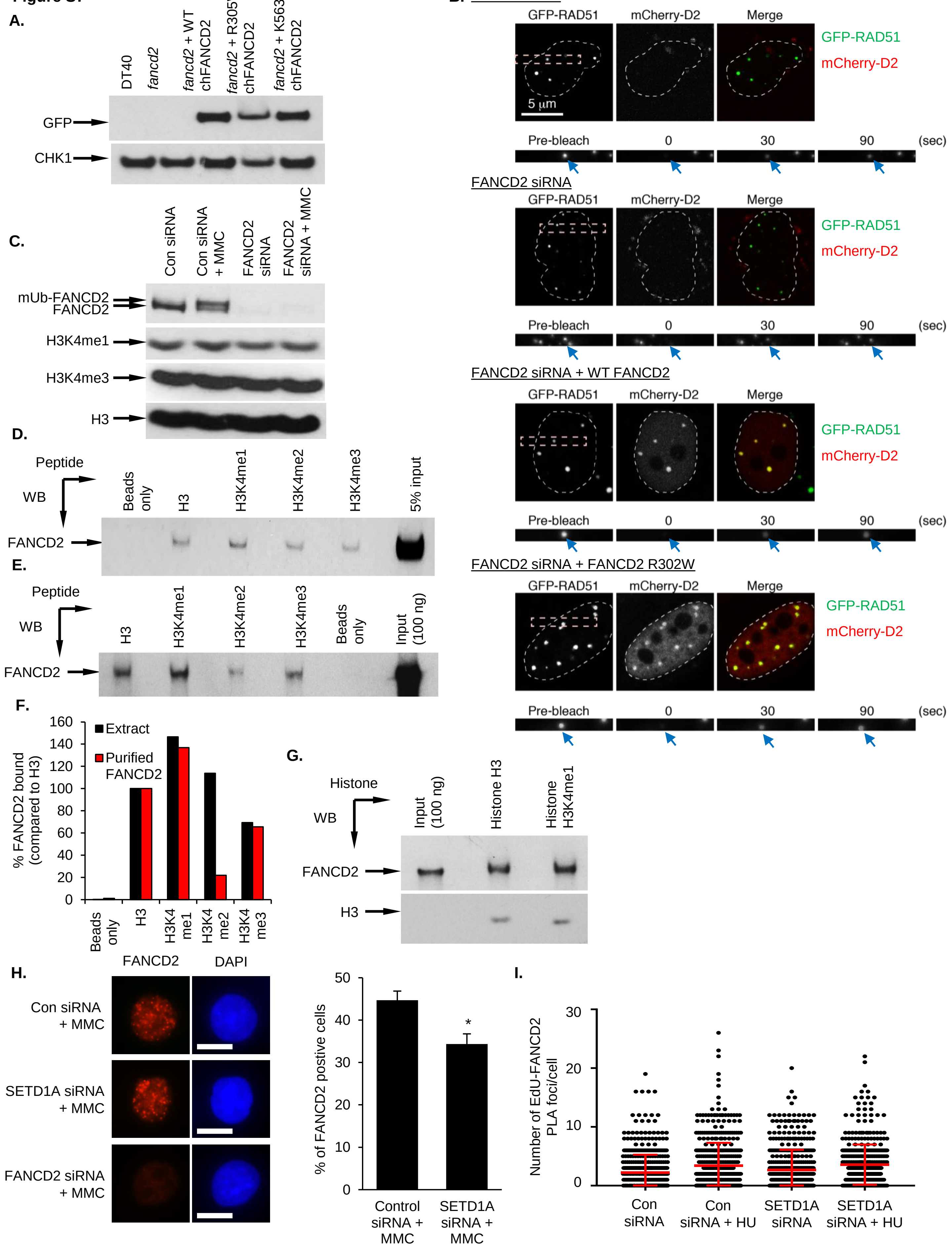
A.
DT40
fancd2
fancd2 + WT chFANCD2
fancd2 + R305W chFANCD2
fancd2 + K563R chFANCD2
GFP
CHK1
B. Control siRNA
GFP-RAD51
mCherry-D2
Merge
5 µm
Pre-bleach
0
30
90
(sec)
FANCD2 siRNA
FANCD2 siRNA + WT FANCD2
FANCD2 siRNA + FANCD2 R302W
C.
Con siRNA
Con siRNA + MMC
FANCD2 siRNA
FANCD2 siRNA + MMC
mUb-FANCD2
FANCD2
H3K4me1
H3K4me3
H3
D.
Peptide
WB
Beads only
H3
H3K4me1
H3K4me2
H3K4me3
5% input
FANCD2
E.
Input (100 ng)
F.
Extract
Purified FANCD2
% FANCD2 bound (compared to H3)
H3K4 me1
H3K4 me2
H3K4 me3
G.
Histone
Histone H3
Histone H3K4me1
H.
FANCD2
DAPI
Con siRNA + MMC
SETD1A siRNA + MMC
FANCD2 siRNA + MMC
% of FANCD2 postive cells
Control siRNA + MMC
SETD1A siRNA + MMC
I.
Number of EdU-FANCD2 PLA foci/cell
Con siRNA
Con siRNA + HU
SETD1A siRNA
SETD1A siRNA + HU

HISTONE METHYLATION BY SETD1A PROTECTS NASCENT DNA THROUGH THE NUCLEOSOME CHAPERONE ACTIVITY OF FANCD2

SUPPLEMENTAL INFORMATION

SUPPLEMENTAL FIGURE LEGENDS

Figure S1, related to Figure 1: BOD1L and SETD1A functionally interact to regulate the replication stress response. (A) *Left:* Schematic comparison of the N-terminus of human BOD1L and the yeast COMPASS-Shg1 protein. *Right:* Amino acid sequence alignment of BOD1L and COMPASS-Shg1. Conserved residues are denoted (red). **(B)** Analysis of proteins that co-precipitate with GST or GST–BOD1L fragments isolated by glutathione-sepharose from HeLa nuclear cell extracts. Inputs and co-precipitates were analyzed by immunoblotting with the indicated antibodies. **(C)** HeLa cells were treated as in Figure 1C. Representative metaphase spreads are shown, with chromosomal damage denoted by arrowheads. **(D)** HeLa cells were transfected with the indicated siRNAs for 72 h, exposed to 50 ng/ml MMC for 24 h, then pulsed for 20 min with sequential pulses of CldU and IdU. DNA was spread onto glass slides and visualised with antibodies to CldU and IdU. DNA fibres were quantified, and the percentage of new origins is displayed.

Figure S2, related to Figure 2: SETD1A protects RAD51 from BLM/FBH1 to prevent over-resection of stalled replication forks. (A) HeLa cells were transfected with the indicated siRNAs for 72 h, exposed to 50 ng/ml MMC for 24 h, and WCE were analysed by immunoblotting. **(B-D)** Transfected HeLa cells from (A) were left untreated or treated with MMC or HU (4 mM for 5 h) as indicated, immunostained with antibodies to antibodies to RPA2 and phospho-RPA S4/S8 (B) or RAD51 (C), and foci formation was analysed by fluorescence microscopy. Plots indicate quantification of foci-positive cells from three independent experiments. Representative images of RAD51 foci are shown in (D). **(E-F)** U-2-OS cells were transfected with the indicated siRNAs for 72 h, pulsed for 20 min each with CldU and IdU, and exposed to 4 mM HU for 5 h. DNA was visualised with antibodies to CldU and IdU, and tract length was calculated. Plots denote the

average ratios of IdU:CldU label length from three independent experiments. Arrows indicate mean ratios. Average values and SEM are denoted in Table S1.

Figure S3, related to Figure 4: The methyltransferase activity of SETD1A is required for fork protection after replication stress. (A) Schematic of the FL, ΔSET and ΔRRM SETD1A constructs. **(B)** U-2-OS Flp-In T-Rex cell lines bearing the indicated SETD1A constructs were exposed to doxycycline for 24 h, and WCE prepared. Extracts were analysed by immunoblotting with the indicated antibodies. **(C)** U-2-OS cells were transfected with the indicated siRNAs for 72 h, exposed to 50 ng/ml MMC for 24 h, and WCE were analysed by immunoblotting. Spaces denote removal of irrelevant lanes. **(D-E)** U-2-OS cells were transfected with the indicated siRNAs for 72 h, and cells were either harvested for immunoblotting (D) or were pulsed for 20 min each with CldU and IdU, and exposed to 4 mM HU for 5 h (E). DNA was visualised with antibodies to CldU and IdU, and tract length was calculated. Plots denote the average ratios of IdU:CldU label length from three independent experiments. Arrows indicate mean ratios. Average values and SEM are denoted in Table S1.

Figure S4, related to Figure 5: SETD1A-mediated H3.1 methylation is required for fork protection after replication stress. (A) WCE of WT DT40s (cl.18), and two clones lacking exons 1-5 and exon 10 of BOD1L (cl.1 and cl.2), were analysed by immunoblotting with the indicated antibodies. **(B)** Data from mass-spectometry analysis of EdU-coprecipitates from HEK293T cells left untreated or pulsed with HU for 4 h. Data is taken from (Dungrawala *et al.,* 2015). **(C)** Quantification of PLA signals between EdU and H3 in U-2-OS cells transfected with the indicated siRNAs. Where denoted, cells were exposed to 4 mM HU for 5 h. The mean±SD number of Biotin/Biotin PLA-signal positive cells (below) was used as control for the number of S-phase cells in each condition. **(D)** HeLa cells expressing WT H3.1-GFP or a K4A mutant (clone D1) were fixed and cell cycle profiles analysed by flow cytometry. Representative profiles with the mean percentages from three independent experiments are shown. **(E)** Plots indicate quantification of cells from Figure 5F, and represent enumeration of cells with >10 foci from three independent experiments. **(F)** WCE of untreated cells from (E) were analysed by immunoblotting with the

indicated antibodies. **(G)** Quantification of the incidence of radial chromosome formation in cells from Figure 5H. **(H)** HeLa cells from (D) were transfected with the indicated siRNAs and treated as above. Cells were immunostained with antibodies to antibodies to RAD51, and cells with >10 foci enumerated. Data represents mean and SEM from three independent experiments. **(I-J)** HeLa cells from (D) were transfected with the indicated siRNAs, pulsed for 20 min each with CldU and IdU, and exposed to 4 mM HU for 5 h (E). DNA was visualised with antibodies to CldU and IdU, and tract length was calculated. Plots denote the average ratios of IdU:CldU label length from three independent experiments. Arrows indicate mean ratios. Average values and SEM are denoted in Table S1.

Figure S5, related to Figure 6: Histone methylation protects against CHD4-mediated fork degradation. (A) WCE of U-2-OS cells from Figure 6A were subjected to immunoblotting using the indicated antibodies. **(B)** U-2-OS cells from Figure 6A were immunostained with antibodies to RPA2, foci formation was analysed by fluorescence microscopy, and fluorescence intensity per nucleus was quantified using ImageJ. Lines denote mean values from three independent experiments. **(C)** HeLa cells were transfected with the indicated siRNAs, exposed to the indicated doses of mitomycin C (MMC), left to form colonies for 14 days, and then stained with methylene blue and colonies counted. Error bars = SEM. **(D)** Quantification of PLA signals between EdU and H3K9me3 in U-2-OS cells transfected with the indicated siRNAs. Where denoted, cells were exposed to 4 mM HU for 5 h. The mean±SD number of Biotin/Biotin PLA-signal positive cells (below) was used as control for the number of S-phase cells in each condition. **(E)** Stable HeLa cells expressing WT H3.1-GFP or K4A (clone D1) were transfected with the indicated siRNAs, pulsed for 20 min each with CldU and IdU, and exposed to 4 mM HU for 5 h. Plots denote the average ratios of IdU:CldU label length from three independent experiments. Arrows indicate mean ratios. Average values, SEM and p values from three independent experiments are denoted in Table S1.

Figure S6, related to Figure 7: SETD1A and FANCD2 promote histone H3 remodelling and fork protection. (A) U-2-OS cells were transfected with the indicated siRNAs, treated as in Figure

S2E, and tract length was calculated. Plots denote the average ratios of IdU:CldU label length, and arrows indicate mean ratios. Average values and SEM from three independent experiments are given in Table S1. **(B-D)** Stable HeLa cells expressing H3.1-GFP WT were transfected with the indicated siRNAs, exposed to 50 ng/ml MMC for 24 h, and the mobility of histone H3.1-GFP was analysed by FRAP. Graphs represent mean relative fluorescence intensities with SD (n = 10-11). In (C) and (D), control siRNA and FANCD2 siRNA data are presented in for comparison. (**E**) Representative images of U-2-OS cells transfected with the indicated siRNAs and expressing newly synthesised SNAP-H3.1 after HU exposure. See Figure 7C for schematic and average values. Scale bar = 10 μm. **(F)** Representative images of stable HeLa cells expressing WT H3.1-GFP or a K4A mutant (clone D1) from Figure 7D. **(G)** Stable HeLa cells from (F) were treated and analysed as in Figure 7D. Plots represent mean relative fluorescence intensities. Error bars indicate SD of values from n= 21-31 cells. Data from Figure 7D (WT and K4A (clone D1)) are presented for comparison. **(H)** Stable HeLa cells from (F) (WT and K4A clone D1) were transfected with the indicated siRNAs, exposed to MMC and analysed by FRAP as above (n = 10-22).

Figure S7, related to Figure 7: H3K4 methylation is required to promote fork protection through FANCD2-dependent histone H3 remodelling. (A) WCE from DT40 cells expressing the denoted GFP-tagged chFANCD2 variants were analysed by immunoblotting. **(B)** Representative FRAP images of stable U-2-OS-GFP-RAD51 cells from Figure 7F after exposure to MMC. Areas analysed are indicated with dashed boxes, and foci analysed by FRAP are denoted with arrows. Represented images are contrast enhanced for display purposes to make foci clearly visible. **(C)** U-2-OS cells were transfected with the indicated siRNAs, exposed to 50 ng/ml MMC for 24 h, and WCE analysed by immunoblotting. **(D)** HeLa nuclear cell extracts were incubated with the indicated immobilised histone peptides, and inputs and immunoprecipitates were analyzed by immunoblotting. **(E)** Purified FANCD2 (100 ng) was incubated with the indicated immobilised histone peptides, and inputs and immunoprecipitates were analyzed by immunoblotting. **(F)** Densitometric quantification of signals from (D-E). Immunoblots from two independent experiments were used. **(G)** Purified FANCD2 (100 ng) was incubated with recombinant histone H3 or an H3K4me1 EPL mimic, and inputs and immunoprecipitates were analyzed by immunoblotting. **(H)**

U-2-OS cells were transfected with the indicated siRNAs, treated as in (C), and immunostained with an antibody to FANCD2. Representative images are shown (*left*), and foci-positive cells were enumerated (*right*). **(I)** Quantification of PLA signals between EdU and FANCD2 in U-2-OS cells from (H). Where denoted, cells were exposed to 4 mM HU for 5 h.

SUPPLEMENTAL TABLE LEGEND

Table S1, related to Figures 3-7: Average values, SEM and p values for fork degradation experiments. The average tract length ratios (IdU:CldU) and SEM for all nascent strand degradation experiments are collated, along with the p values obtained for each condition relative to relevant control cells (denoted with '-'). ND = not determined.
