# Supplementary material for: Histone Methylation by SETD1A Protects Nascent DNA through the Nucleosome Chaperone Activity of FANCD2
Source: Mol Cell. 2018 Jul 5;71(1):25–41.e6. doi: 10.1016/j.molcel.2018.05.018 (PMC6039718; doi:10.1016/j.molcel.2018.05.018)
Supplement: Document S2. Article plus Supplemental Information [file mmc3.pdf]

Article

Molecular Cell

Histone Methylation by SETD1A Protects Nascent DNA through the Nucleosome Chaperone Activity of FANCD2

Graphical Abstract

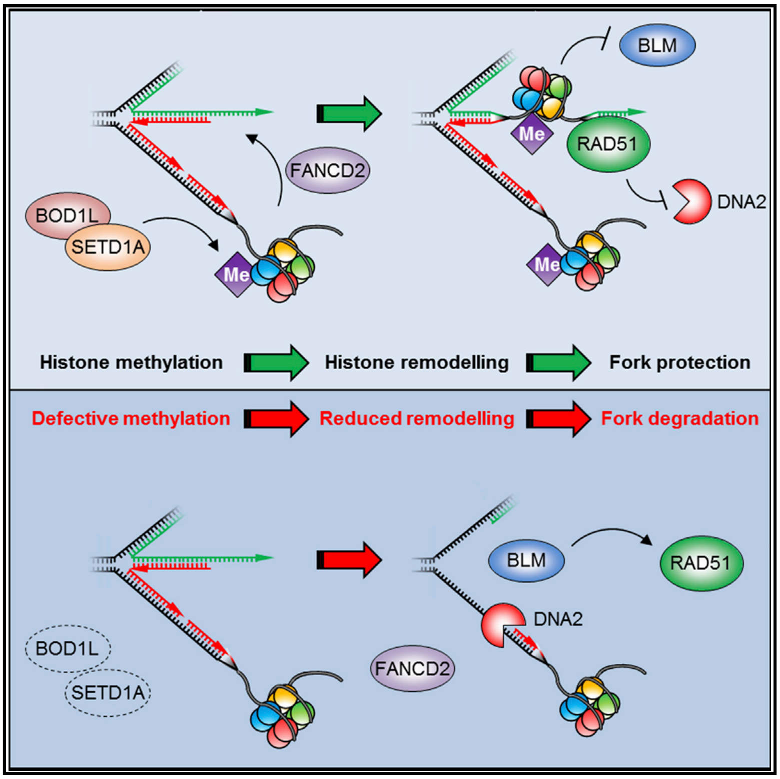

Authors

Martin R. Higgs, Koichi Sato, John J. Reynolds, ..., Hitoshi Kurumizaka, Hiroshi Kimura, Grant S. Stewart

Correspondence

m.r.higgs@bham.ac.uk (M.R.H.), g.s.stewart@bham.ac.uk (G.S.S.)

In Brief

Higgs et al. identify histone H3K4 methylation by SETD1A as essential to prevent genome instability during replication stress by enhancing FANCD2-dependent nucleosome remodeling. Loss of SETD1A or deficiencies in H3 mobilization by FANCD2 leads to RAD51 nucleofilament instability and severe nucleolytic degradation of stalled replication forks.

Highlights

- Methylation of H3K4 by SETD1A maintains genome stability during replication stress
- SETD1A and H3K4 methylation stabilize RAD51 nucleofilaments to protect nascent DNA
- SETD1A-dependent H3K4 methylation enhances FANCD2-dependent histone remodeling
- Histone mobility stabilizes RAD51 nucleofilaments to inhibit fork degradation

Higgs et al., 2018, Molecular Cell *71*, 25–41
July 5, 2018 © 2018 The Author(s). Published by Elsevier Inc.
https://doi.org/10.1016/j.molcel.2018.05.018

CellPress

Histone Methylation by SETD1A Protects Nascent DNA through the Nucleosome Chaperone Activity of FANCD2

Martin R. Higgs,[1,*] Koichi Sato,[2,8] John J. Reynolds,[3] Shabana Begum,[1] Rachel Bayley,[1] Amalia Goula,[1] Audrey Vernet,[3] Karissa L. Paquin,[4] David G. Skalnik,[5] Wataru Kobayashi,[2] Minoru Takata,[6] Niall G. Howlett,[4] Hitoshi Kurumizaka,[2] Hiroshi Kimura,[7] and Grant S. Stewart[3,9,*]

[1]Lysine Methylation and DNA Damage Laboratory, Institute of Cancer and Genomic Sciences, University of Birmingham, Birmingham B15 2TT, UK
[2]Department of Electrical Engineering and Bioscience, Waseda University, Shinjuku, Tokyo 169-8050, Japan
[3]Genome Stability and Human Disease Laboratory, Institute of Cancer and Genomic Sciences, University of Birmingham, Birmingham B15 2TT, UK
[4]Department of Cell and Molecular Biology, University of Rhode Island, Kingston, RI 02881, USA
[5]Biology Department, School of Science, Indiana University-Purdue University Indianapolis, Indianapolis, IN 46202, USA
[6]Laboratory of DNA Damage Signaling, Department of Late Effects Studies Radiation Biology Center, Kyoto University Yoshida konoe cho, Sakyo ku, Kyoto 606-8501, Japan
[7]Cell Biology Center, Institute of Innovative Research, Tokyo Institute of Technology, Kanagawa Prefecture 226-8501, Japan
[8]Present address: Hubrecht Institute–KNAW and University Medical Center Utrecht, 3584 CT Utrecht, the Netherlands
[9]Lead Contact
*Correspondence: m.r.higgs@bham.ac.uk (M.R.H.), g.s.stewart@bham.ac.uk (G.S.S.)
https://doi.org/10.1016/j.molcel.2018.05.018

SUMMARY

Components of the Fanconi anemia and homologous recombination pathways play a vital role in protecting newly replicated DNA from uncontrolled nucleolytic degradation, safeguarding genome stability. Here we report that histone methylation by the lysine methyltransferase SETD1A is crucial for protecting stalled replication forks from deleterious resection. Depletion of SETD1A sensitizes cells to replication stress and leads to uncontrolled DNA2-dependent resection of damaged replication forks. The ability of SETD1A to prevent degradation of these structures is mediated by its ability to catalyze methylation on Lys4 of histone H3 (H3K4) at replication forks, which enhances FANCD2-dependent histone chaperone activity. Suppressing H3K4 methylation or expression of a chaperone-defective FANCD2 mutant leads to loss of RAD51 nucleofilament stability and severe nucleolytic degradation of replication forks. Our work identifies epigenetic modification and histone mobility as critical regulatory mechanisms in maintaining genome stability by restraining nucleases from irreparably damaging stalled replication forks.

INTRODUCTION

To maintain genome stability during genome duplication, numerous cellular pathways have evolved to detect and repair structures and/or lesions that impair DNA replication. One key response to compromised replication (known as replication stress) involves the active reversal of stalled replication forks to form 4-way DNA junctions. This represents a critical step in stabilizing damaged forks (Neelsen and Lopes, 2015) and involves homologous recombination (HR) factors such as RAD51 (Wang et al., 2015; Zellweger et al., 2015). However, despite the importance of fork reversal in protecting genome stability, it is also clear that the regressed arms of reversed forks are highly susceptible to nucleolytic degradation (Thangavel et al., 2015). Although controlled processing of these structures can help to maintain fork integrity and allow fork restart, uncontrolled degradation of nascent DNA leads to severe genome instability (Quinet et al., 2017).

Components of the Fanconi anemia (FA) and HR pathways, including RAD51 (FANCR), FANCD2, BRCA1 (FANCS), and BRCA2 (FANCD1), play a vital role in protecting nascent DNA at reversed replication forks (Schlacher et al., 2011, 2012; Somyajit et al., 2015; Ying et al., 2012; Zadorozhny et al., 2017). Moreover, the deleterious resection of replication forks observed in the absence of these factors can be prevented by limiting fork reversal via depletion of "pro-reversal" factors; e.g., SMARCAL1 (Mijic et al., 2017; Taglialatela et al., 2017). However, despite intensive study, the mechanisms by which cells protect nascent DNA still remain poorly understood. It is imperative that we better understand these processes because restoring fork protection in tumor cells facilitates their ability to evade chemotherapy and acquire drug resistance (Ray Chaudhuri et al., 2016).

Presently, it is unclear how reversed replication forks requiring protection are marked; this may involve the presence of specific factors, post-translational modification of the replication machinery and/or surrounding chromatin, and/or chromatin remodeling. In keeping with the premise that histone dynamics may

This is an open access article under the CC BY license (http://creativecommons.org/licenses/by/4.0/).

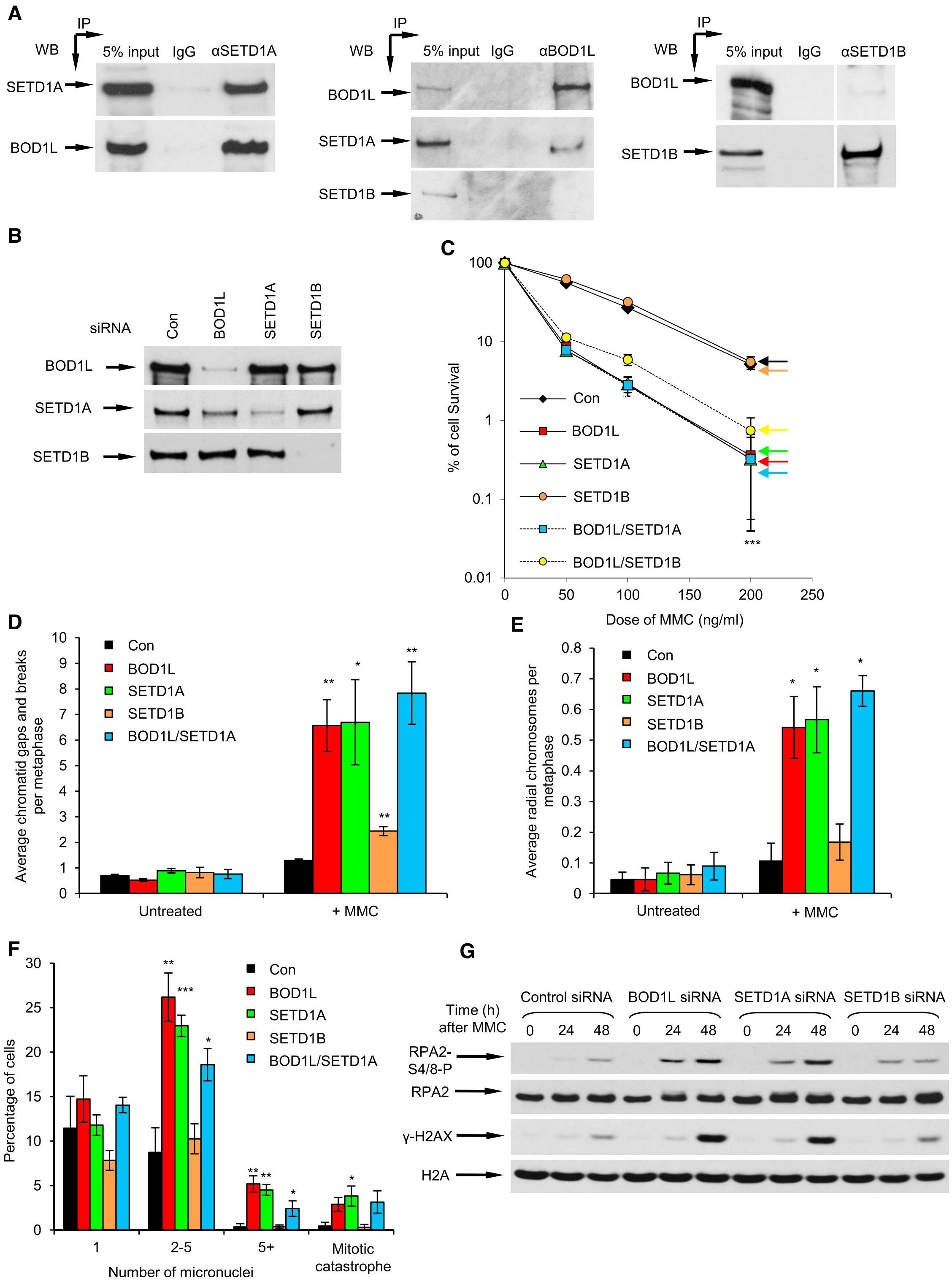

Figure 1. BOD1L Acts with SETD1A to Maintain Genome Stability during Replication Stress

(A) HeLa nuclear cell extracts were subjected to immunoprecipitation with the denoted antibodies, and inputs and immunoprecipitates were analyzed by immunoblotting. White lines denote removal of irrelevant lanes.

(B) HeLa cells were transfected with the indicated siRNAs for 72 hr and whole-cell extracts (WCEs) were analyzed by immunoblotting.

(legend continued on next page)

play an important role in this process, members of the SNF2 family of remodeling ATPases promote fork degradation in the absence of protective factors (Taglialatela et al., 2017). Moreover, the fork protection factor FANCD2 also remodels histones at sites of replication stress (Sato et al., 2012). Interestingly, several chromatin modifiers have also been implicated in preventing fork degradation: the lysine methyltransferase (KMT) EZH2 regulates recruitment of MUS81 to stalled forks (Rondinelli et al., 2017), whereas the KMTs KMT2C/KMT2D (MLL2/3) enhance MRE11-dependent fork processing (Ray Chaudhuri et al., 2016). In contrast, the yeast KMT Set1, a component of the evolutionarily conserved "complex proteins associated with Set1p" (COMPASS) that catalyzes methylation of lysine 4 of histone H3 (H3K4), is required in the response to replication stress (Faucher and Wellinger, 2010).

Recently, we identified BOD1L as a fork protection factor that protects nascent DNA from degradation by DNA2 (Higgs et al., 2015; Higgs and Stewart, 2016). Here we demonstrate that BOD1L functionally interacts with the KMT SETD1A and that cells lacking SETD1A phenocopy those depleted of BOD1L. Furthermore, we show that SETD1A methylates H3K4 at stalled replication forks, which facilitates the mobilization of histones by FANCD2 and prevents replication fork degradation. Compromising H3K4 methylation abrogates FANCD2-dependent histone chaperone activity, leads to fork degradation, and mimics the inability of cells lacking SETD1A to recruit RAD51 to stalled forks. Our data therefore establish that SETD1A-dependent histone methylation and subsequent histone remodeling protect stalled forks from uncontrolled processing, thereby maintaining genome stability.

RESULTS

BOD1L Interacts with SETD1A to Regulate Genome Stability Following Replication Stress

We recently identified BOD1L as a factor that protects stalled replication forks from degradation (Higgs et al., 2015). Previous studies (van Nuland et al., 2013) have suggested that BOD1L forms complexes with the KMTs SETD1A and SETD1B, two closely related members of the KMT2 family that catalyze H3K4 methylation (Bledau et al., 2014; Brici et al., 2017; Lee and Skalnik, 2005; Lee et al., 2007). This suggested that SETD1A and/or SETD1B may function with BOD1L to regulate replication fork stability. We therefore first sought to confirm these interactions before investigating any potential role of these enzymes in fork protection. Interestingly, reciprocal immunoprecipitations confirmed that BOD1L interacts with SETD1A, but not with SETD1B, as was suggested previously (van Nuland et al., 2013; Figure 1A).

The N-terminal region of BOD1L contains a region with sequence homology to the Shg1 component of the yeast COMPASS complex (Figure S1A) (PFAM: 05205; http://pfam.xfam.org/family/PF05205) (Marchler-Bauer et al., 2011; Roguev et al., 2001). We hypothesized that this "COMPASS-Shg1" domain may, by analogy, mediate the interaction of BOD1L with the SETD1A complex. To assess this, we generated glutathione S-transferase (GST)-tagged fragments of BOD1L spanning this domain or neighboring regions and analyzed their ability to interact with SETD1A. These experiments revealed that a fragment of BOD1L containing this COMPASS-Shg1 domain was necessary and sufficient to mediate interaction with SETD1A but not with SETD1B (Figure S1B).

To analyze the functional consequences of this interaction, we depleted HeLa cells of BOD1L, SETD1A, or SETD1B, either alone or in combination (Figure 1B), and exposed them to mitomycin C (MMC), which induces DNA interstrand crosslinks (ICLs). Notably, depletion of SETD1A or BOD1L alone exquisitely hypersensitized cells to MMC (Figure 1C). Moreover, cells lacking SETD1A exhibited elevated chromosomal damage (Figures 1D, 1E, and S1C) and increased micronucleus formation after treatment with MMC (Figure 1F), indicating a critical role for this KMT in maintaining genome stability after replication damage. In all cases, loss of SETD1A alone or alongside BOD1L was comparable with BOD1L depletion, consistent with these two factors residing within the same protein complex. In contrast, depletion of SETD1B had no effect on cellular sensitivity to replication stress (Figure 1), in keeping with our interaction data, although we cannot completely exclude a role of this enzyme in regulating genome stability. Moreover, cells lacking SETD1A or BOD1L, but not SETD1B or BOD1 (a BOD1L paralog), were unable to suppress replication origin firing after MMC exposure (Figure S1D), a characteristic of BOD1L deficiency (Higgs et al., 2015). Together with our previous data demonstrating that depletion of BOD1 had no effect on MMC cellular sensitivity (Higgs et al., 2015), these observations are consistent with a model in which BOD1L/SETD1A and BOD1/SETD1B form functionally distinct KMT complexes.

SETD1A and BOD1L Suppress BLM/FBH1 to Stabilize RAD51 and Prevent DNA2-Dependent Degradation of Nascent DNA

Preventing aberrant replication fork resection is essential for genome integrity during replication stress. The RAD51 recombinase plays a crucial role in protecting stalled forks from such degradation (Schlacher et al., 2011, 2012; Zadorozhny et al., 2017; Zellweger et al., 2015); indeed, our previous studies demonstrated that BOD1L suppresses degradation of stalled replication forks by stabilizing RAD51 at these sites (Higgs et al., 2015). To investigate whether SETD1A functioned in a similar fashion, we first analyzed DNA resection in cells depleted of BOD1L, SETD1A, or SETD1B, using phosphorylation of RPA2

(C) HeLa cells were transfected as above, exposed to the indicated doses of MMC, and left for 14 days, and colonies were stained with methylene blue and counted.

(D and E) Cells from (C) were exposed to 50 ng/mL MMC for 20 hr and treated with colcemid for a further 4 hr. Chromosomal damage (D) and radial chromosome formation (E) were enumerated by light microscopy after Giemsa staining. Representative images are shown in Figure S1.

(F) Cells from (C) were treated with MMC and left for 24 hr, and micronucleus formation was assessed by fluorescence microscopy.

(G) Cells from (C) were exposed to MMC for the indicated times, and WCE was analyzed by immunoblotting.

The plots in (C)–(E) represent mean data ± SEM from 3 independent experiments. *$p < 0.05$, **$p < 0.01$, and ***$p < 0.001$. See also Figure S1.

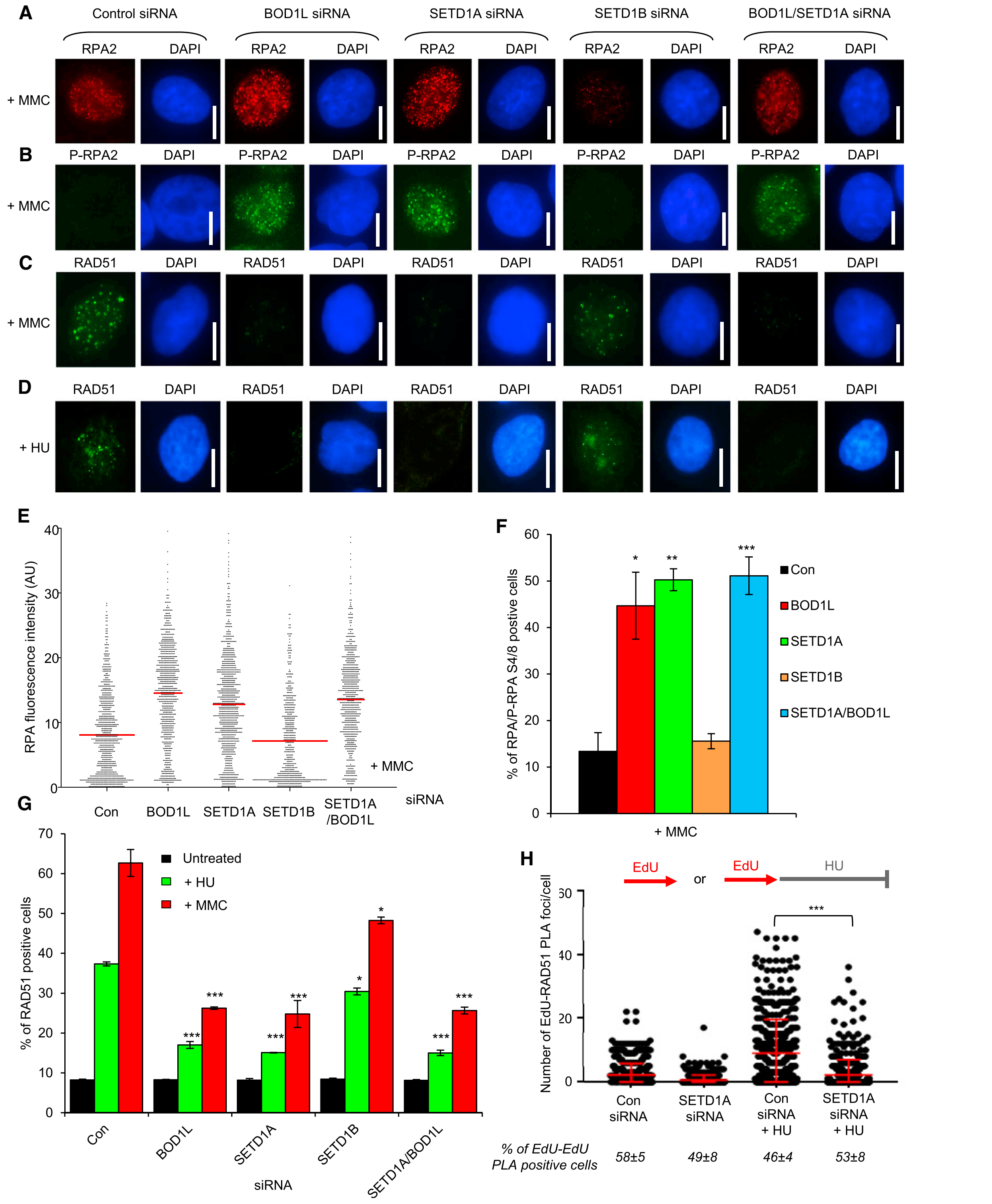

Figure 2. SETD1A Prevents Excessive ssDNA Formation after Replication Stress by Stabilizing RAD51

(A–C) HeLa cells were transfected with the indicated siRNAs for 72 hr; exposed to 50 ng/mL MMC for 24 hr; immunostained with antibodies to RPA2 (A), phospho-RPA2 S4/S8 (B), or RAD51 (C). Focus formation was analyzed by fluorescence microscopy.

(D) Cells from (A) were exposed to 4 mM HU for 5 hr and immunostained with antibodies to RAD51, and focus formation was analyzed as above.

(E) Nuclear fluorescence intensity of cells from (A) was quantified using ImageJ. Lines denote mean values.

(F) Double-positive (RPA- and phospho-RPA2) cells from (A) and (B) were enumerated and are displayed as a percentage of total cells.

(legend continued on next page)

on S4/S8 as a well-established marker of resected DNA. Interestingly, levels of MMC-induced RPA2-P-S4/8 were substantially elevated upon loss of BOD1L or SETD1A but not SETD1B (Figures 1G, 2A, and 2B), consistent with increased generation of single-stranded DNA (ssDNA). Moreover, similar to BOD1L, SETD1A was required to recruit or stabilize RAD51 at stalled forks upon exposure to either MMC or hydroxyurea (HU) (Figures 2C and 2D). Co-depletion of SETD1A and BOD1L had no additional effect on RPA/RPA2-P-S4/8 or RAD51 focus formation, again suggesting that these factors act together (Figures 2B–2G). Next, using 5-ethynyl-2'-deoxyuridine (EdU)-Click coupled to proximity ligation (PLA) (Petruk et al., 2012; Taglialatela et al., 2017), we analyzed whether the association of RAD51with nascent DNA was affected by loss of SETD1A. In agreement with our previous data, SETD1A depletion significantly decreased the levels of RAD51 on newly replicated DNA after HU exposure (Figure 2H).

Given the importance of RAD51 in suppressing deleterious fork processing, we next assessed whether SETD1A was required to protect nascent DNA at replication forks. To this end, we used a well-characterized fork protection assay (Schlacher et al., 2011) to monitor the stability of nascent DNA during prolonged replication arrest by HU. Loss of SETD1A, but not SETD1B, increased the degradation of HU-stalled forks, apparent as a decreased iododeoxyuridine (IdU):chlorodeoxyuridine (CldU) ratio (Figure 3A; Table S1), supporting our hypothesis that SETD1A and SETD1B are functionally distinct. Moreover, the fork degradation observed upon BOD1L loss was comparable with that arising from SETD1A depletion (Figure 3B; Table S1). Because fork remodeling enzymes such as SMARCAL1 catalyze fork reversal and, thus, provide a substrate for nucleolytic degradation in the absence of protective factors (Kolinjivadi et al., 2017), we investigated whether loss of SMARCAL1 suppressed fork resection observed upon SETD1A loss. Indeed, depletion of this annealing helicase reduced nascent strand degradation in cells lacking SETD1A (Figure 3C; Table S1). Together, this suggests that SETD1A-BOD1L prevent the resection of reversed replication forks.

Recent studies have suggested that other members of the KMT2 family (KMT2C [MLL3] and KMT2D [MLL2]) promote nascent strand degradation in the absence of BRCA2 (Ray Chaudhuri et al., 2016). However, although we also observed that depletion of KMT2C/KMT2D rescued fork stability in the absence of BRCA2, this was not the case when these factors were co-depleted in combination with SETD1A (Figures 3D–3F; Table S1). Moreover, in stark contrast to cells lacking SETD1A, loss of KMT2C/D alone had no effect on fork stability after HU. These data reinforce the notion that different KMT2 enzymes have diverse functions during replication stress.

Factors that regulate the stability of RAD51 filaments, such as PARI, BLM, FBH1, and the RAD51 paralogs, also play vital roles in maintaining replication fork stability (Mochizuki et al., 2017; Somyajit et al., 2015). Previous work has demonstrated that uncontrolled BLM/FBH1 activity can destabilize RAD51 nucleofilaments at stalled replication forks, leading to fork degradation (Higgs et al., 2015; Higgs and Stewart, 2016; Leuzzi et al., 2016). We therefore predicted that SETD1A may also counteract the activities of these two anti-recombinases to stabilize RAD51 at stalled forks. To investigate this, we co-depleted SETD1A and BLM (Figure S2A), exposed the cells to MMC, and monitored RPA S4/S8 phosphorylation and RAD51 focus formation. Strikingly, loss of BLM reduced MMC-induced RPA S4/S8 phosphorylation (Figure S2B) and restored HU- and MMC-induced RAD51 focus formation in the absence of SETD1A (Figures S2C and S2D). Moreover, depletion of either FBH1 or BLM abrogated the degradation of nascent DNA observed in cells lacking SETD1A (Figure S2E; Table S1).

Because BOD1L, RAD51, and the FA pathway are crucial for suppressing fork resection by DNA2 (Higgs et al., 2015; Karanja et al., 2014; Liu et al., 2016), we assessed whether the over-resection observed in the absence of SETD1A was dependent on DNA2 or whether other nucleases implicated in fork resection were also involved. In keeping with our previous observations in cells lacking BOD1L (Higgs et al., 2015), co-depletion of DNA2, but not EXO1 or MRE11, suppressed the degradation of HU-stalled forks in the absence of SETD1A (Figure S2F; Table S1). Therefore, SETD1A and BOD1L act together to stabilize RAD51 on nascent DNA by restraining the anti-recombinase functions of BLM/FBH1, protecting damaged replication forks from DNA2-dependent resection.

The Catalytic Activity of SETD1A Is Required for Fork Protection

Previous studies have demonstrated that the methyltransferase activity of SETD1A toward H3K4 is mediated by its C-terminal N-SET (COMPASS component N-Su(var)3-9, Enhancer-of-zeste, Trithorax domain) and SET catalytic domains, whereas interactions with WDR82, RNA, and RNA polymerase II (Pol II) occur via the N-terminal RRM domain (Lee and Skalnik, 2008; Luciano et al., 2017; Schlichter and Cairns, 2005). To examine which of these domains is required for fork protection by SETD1A, we established U-2-OS cell lines in which endogenous SETD1A could be depleted with small interfering RNA (siRNA), and either full-length (FL) FLAG-tagged SETD1A or variants lacking the RRM (ΔRRM) or catalytic SET (ΔSET) domains could be inducibly expressed (Figures S3A and S3B). Strikingly, the genome instability (Figure 4A), defective RAD51 focus formation (Figures 4B and 4C), and increased fork degradation (Figure 4D) observed in the absence of endogenous SETD1A were all restored following induced expression of FL and ΔRRM SETD1A, but not the catalytically inactive ΔSET variant. This suggests that the role for SETD1A in resolving replication stress is independent of its ability to regulate transcription. This contrasts with recent publications suggesting that SETD1A

(G) The mean percentage of cells from (C) and (D) exhibiting RAD51 foci was enumerated as above.

(H) Quantification of PLA signals between EdU and RAD51 in U-2-OS cells transfected with the indicated siRNAs. Where denoted, cells were exposed to 4 mM HU for 5 hr (as shown in the schematic). The mean ± SD of the number of biotin/biotin PLA signal-positive cells (below) indicates the number of S phase cells in each condition.

The plots in (E)–(H) represent mean data ± SEM from 3 independent experiments. Scale bars, 10 μm. *$p < 0.05$, **$p < 0.01$, and ***$p < 0.001$. See also Figure S2.

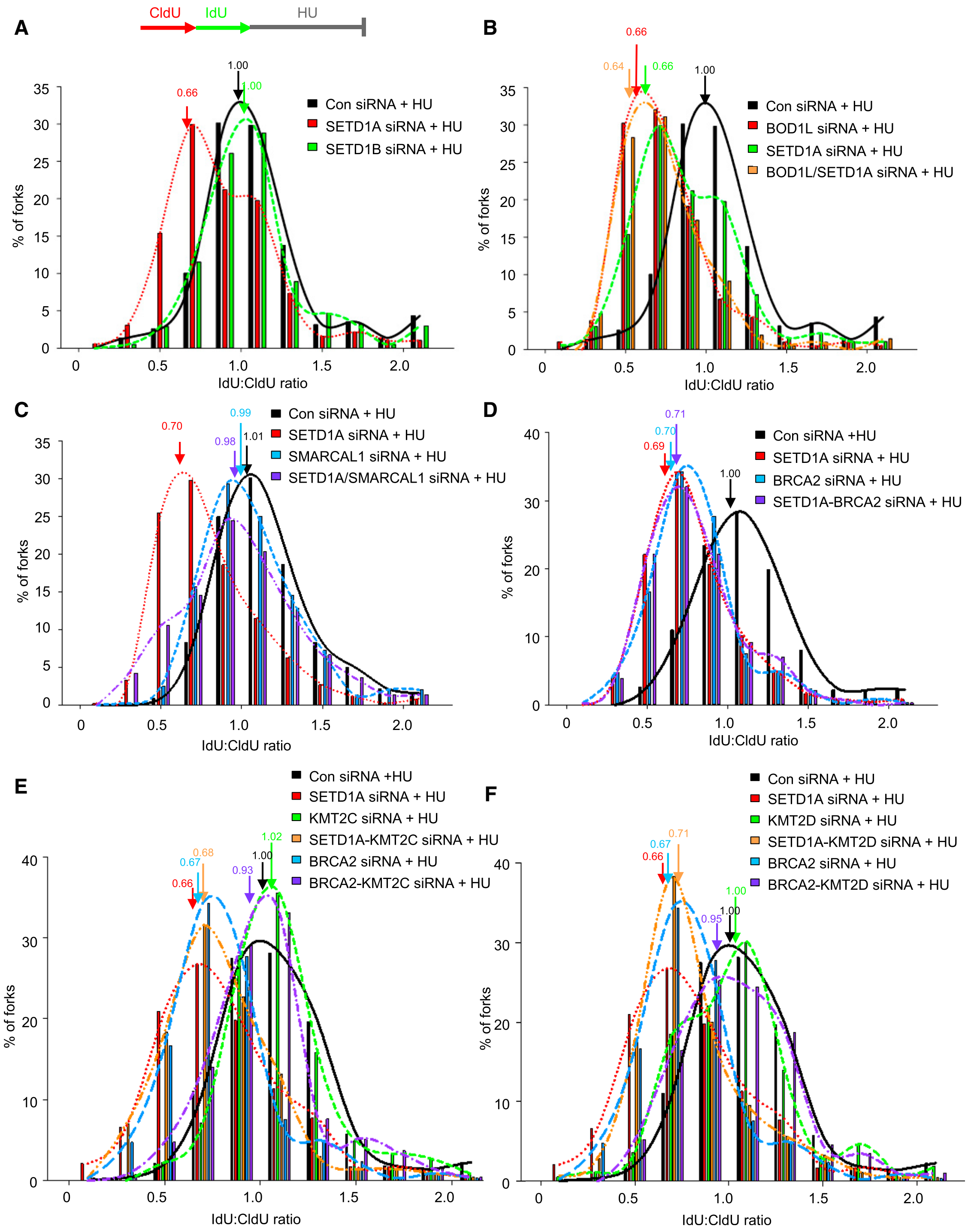

Figure 3. SETD1A Suppresses Fork Degradation after Replication Stress

(A–F) U-2-OS cells were transfected with control siRNA or those targeting: SETD1A and SETD1B (A); BOD1L and SETD1A (B); SETD1A and SMARCAL1 (C); SETD1A and BRCA2 (D); SETD1A, BRCA2, and either KMT2C (E), or KMT2D (F). 72 hr post transfection, cells were pulsed for 20 min each with CldU and IdU and exposed to 4 mM HU for 5 hr (as in the schematic). DNA was visualized with antibodies to CldU and IdU, and tract lengths were calculated. Plots denote average ratios of IdU:CldU label length from 3 independent experiments; arrows indicate mean ratios. Plots in (E) and (F) amalgamate data from the same experiments. See also Table S1.

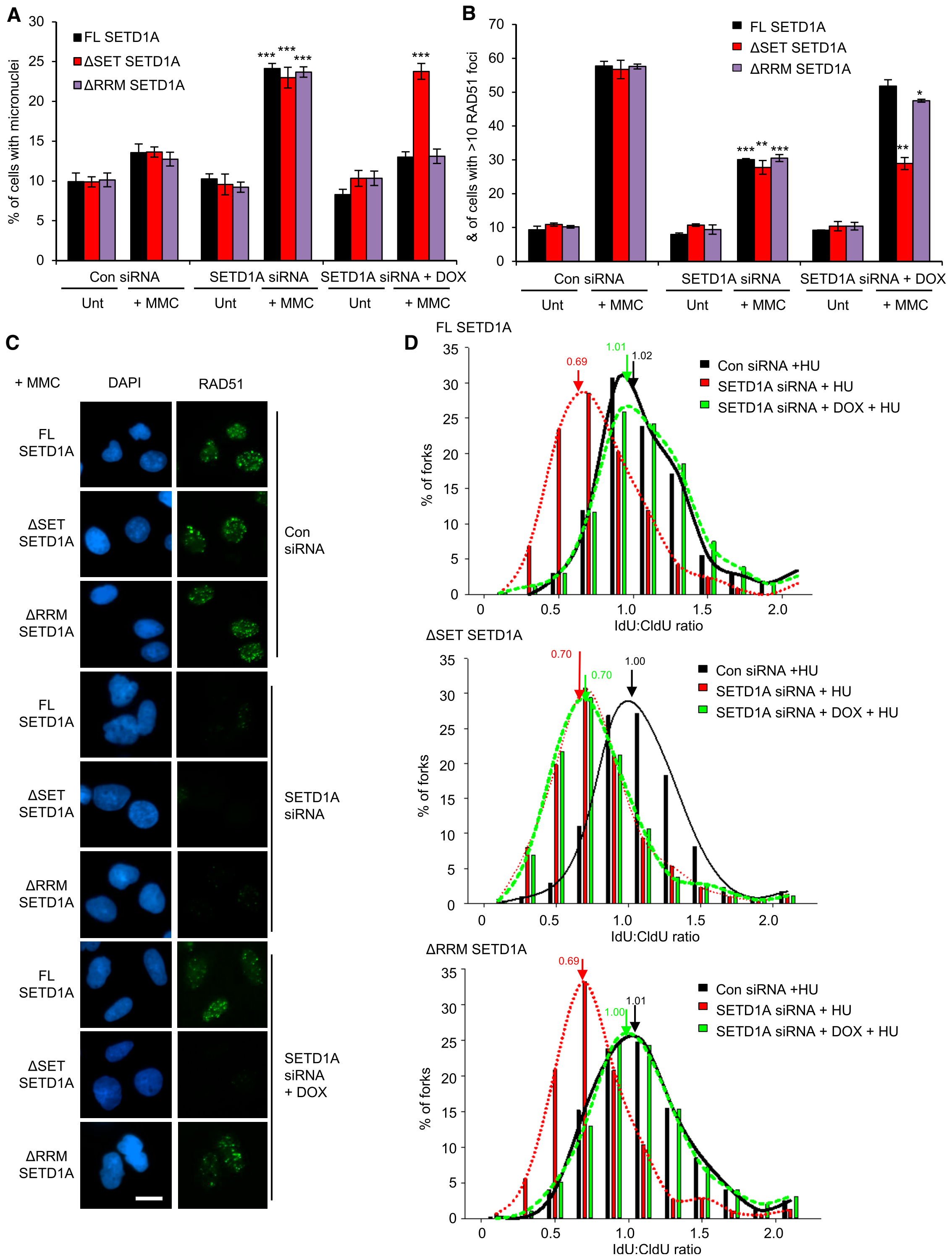

Figure 4. The KMT Activity of SETD1A Is Required to Protect Nascent DNA from Degradation

(A) U-2-OS cell lines bearing inducible full-length (FL) SETD1A or mutants lacking the RRM (ΔRRM) or N-SET and SET (ΔSET) domains were transfected with the indicated siRNAs for 48 hr, exposed to 1 μg/mL doxycycline for 24 hr where denoted, and then exposed to 50 ng/mL MMC for a further 24 hr. Micronucleus formation was quantified by fluorescence microscopy.

(legend continued on next page)

functions within the DNA damage response (DDR) in hematopoietic cells to regulate the transcription of a subset of DDR genes, in part via association with CDK12/Cyclin K (Arndt et al., 2018; Hoshii et al., 2018). To address this disparity, we therefore examined the protein levels of DDR genes identified to be deregulated in the absence of SETD1A. These analyses revealed little or no change in the expression of these DDR proteins upon SETD1A depletion (Figure S3C). Moreover, and in support of a transcription-independent role of SETD1A in regulating fork protection, depletion of Cyclin K had no effect on nascent DNA degradation of HU-stalled forks (Figures S3D and S3E) despite its apparent role in SETD1A-dependent transcription (Hoshii et al., 2018). Our findings therefore demonstrate that the catalytic methyltransferase activity of SETD1A is necessary for its role in fork protection. Importantly, this function is unlikely to be mediated through regulation of DDR gene transcription or via an interaction with Cyclin K.

SETD1A Catalyzes H3K4 Methylation at Replication Forks to Prevent Deleterious Fork Processing

Because the catalytic activity of SETD1A protects nascent DNA from degradation, we next wanted to assess the effect of depleting SETD1A or its closely related paralog, SETD1B, on H3K4 methylation. Interestingly, depletion of these separate KMT components from HeLa cells led to a significant, albeit different, reduction in levels of global H3K4 mono-methylation (H3K4me1), with depletion of SETD1A or SETD1B having effects comparable with loss of BOD1L or BOD1, respectively (Figure 5A), a finding recapitulated in two independent DT40 BOD1L knockout clones (Figure S4A). These data suggest that, although SETD1A and SETD1B KMT complexes both target the same residue on histone H3, their loss has a differential effect on total levels of H3K4 methylation.

Given our findings, we next investigated whether SETD1A catalyzed H3K4 methylation specifically at stalled or reversed replication forks during replication stress. We first used isolation of proteins on nascent DNA (iPOND) coupled with mass spectrometry to assess protein abundance on newly replicated DNA. This approach demonstrated that components present in all KMT2 complexes (WDR5, RBBP5, and ASH2L) and those only found in the SETD1A and SETD1B KMT complexes (WDR82 and HCFC1) all associate with nascent DNA and HU-stalled forks (Figure S4B). Moreover, SETD1A, but not SETD1B, was also detected at these sites, albeit infrequently. We next analyzed whether mono-methylated H3K4 at forks was affected by replication stress or by loss of SETD1A or SETD1B using EdU-PLA. This revealed that the association of H3K4me1 with nascent DNA increased upon exposure to HU in a manner dependent on SETD1A (Figure 5B). Importantly, this was not due to any global changes in histone abundance after HU exposure, since levels of H3 on nascent DNA were unaffected by either HU or SETD1A loss (Figure S4C). Moreover, this effect was specific to H3K4me1 because the levels of H3K4me3 at similar sites were markedly less affected by SETD1A loss (Figure 5C). These data suggest that SETD1A catalyzes H3K4me1 at stalled replication forks.

To directly investigate the effect of H3K4 methylation on replication fork resection, we disrupted the balance of steady-state methylation of H3K4 by creating stable HeLa cell lines expressing wild-type (WT) GFP-tagged histone H3.1 or a K4A mutant. Expression of these variants in addition to endogenous H3.1 had no overt effects on cell cycle progression, regardless of the presence of replication stress (Figure S4D). Next, we exposed these cells to prolonged HU treatment and then assayed for loss of fork protection. Strikingly, mutation of Lys4 of H3 recapitulated the elevated fork degradation observed in cells lacking BOD1L or SETD1A (Figure 5D). Notably, loss of SETD1A in cells expressing the H3K4A mutant had no additional effect on fork resection (Figure 5E), further reinforcing the hypothesis that this KMT complex mediates fork protection via H3K4 methylation.

To analyze whether H3K4 methylation affected the stability of RAD51 at stalled or damaged replication forks, we next monitored RAD51 focus formation in these cells. Strikingly, the formation of MMC- and HU-induced RAD51 foci was severely compromised by expression of H3.1-GFP K4A but not by its WT counterpart (Figures 5F and S4E). This was recapitulated in two separate cell clones and could not be accounted for by any alterations in RAD51 or H3-GFP protein expression (Figure S4F). Furthermore, expression of the K4A variant significantly decreased the levels of RAD51 on nascent DNA after HU exposure (Figure 5G), resulted in MMC hyper-sensitivity (Figure 5H), and increased the levels of MMC-induced chromosomal damage (Figures 5I andS4G). Consistent with a model in which BOD1L, SETD1A, and H3K4 methylation act together to protect RAD51 from the destabilizing activity of BLM, depletion of BLM in cells expressing H3.1-GFP K4A restored RAD51 focus formation (Figure S4H) and abrogated fork degradation (Figure S4I). Finally, depletion of DNA2 in cells expressing H3.1-GFP K4A also restored fork stability (Figure S4J), in line with a role for H3K4 methylation in preventing fork processing by this exonuclease.

Together, these data suggest that the failure of cells lacking SETD1A to stabilize RAD51 at stalled replication forks and the subsequent inability to protect these structures from degradation are intimately linked to a defect in H3K4 methylation on nascent DNA.

H3.1 Methylation Suppresses CHD4-Mediated Fork Degradation

To investigate the mechanisms underlying methylation-dependent fork protection, we first examined the involvement of methyl-histone "reader" proteins. Members of the chromodomain helicase DNA-binding (CHD) family play important roles in replication stress, and their activities or chromatin localization are intimately linked with H3K4 methylation status. In particular,

(B and C) Cells from (A) were immunostained with antibodies to RAD51, and focus formation was analyzed by fluorescence microscopy (B). Representative images are shown in (C). Scale bar, 20 μm.

(D) Cells from (A) were treated as described in Figure 3; arrows denote mean ratios.

Plots represent mean data ± SEM from 3 independent experiments. $^{*}p < 0.05$, $^{**}p < 0.01$, and $^{***}p < 0.001$. See also Figure S3 and Table S1.

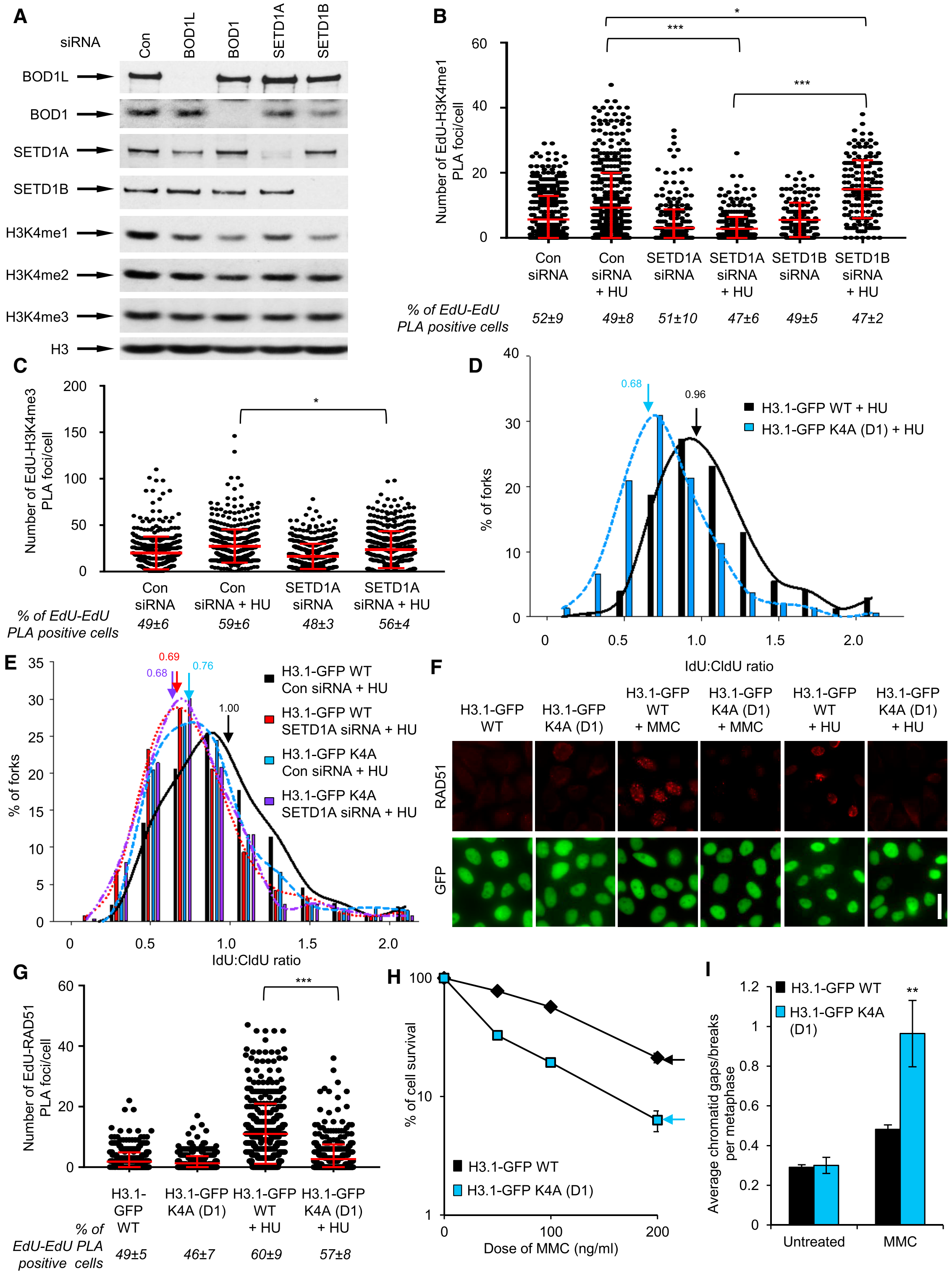

Figure 5. SETD1A-Mediated H3K4 Methylation Is Required for Fork Protection

(A) HeLa cells were transfected with the indicated siRNAs for 72 hr, and WCEs were analyzed by immunoblotting.

(B and C) Quantification of PLA signals between EdU and H3K4me1 (B) or H3K4me3 (C) in U-2-OS cells transfected with the indicated siRNAs. Cells were exposed to 4 mM HU for 5 hr where denoted.

(legend continued on next page)

the H3K4 methyl-binding protein CHD1 is implicated in DNA resection and RAD51 loading (Kari et al., 2016; Shenoy et al., 2017; Sims et al., 2005), whereas the nucleosome remodeling deacetylase (NuRD) component and tri-methylated lysine 9 of histone H3 (H3K9me3) reader CHD4 is negatively regulated by H3K4 methylation and promotes nascent DNA degradation in BRCA2-deficient cells (Guillemette et al., 2015; Ray Chaudhuri et al., 2016; Watson et al., 2012).

We therefore predicted that SETD1A-dependent fork protection may involve either of these remodelers. To examine this, we co-depleted SETD1A and either CHD1 or CHD4 and examined the effect on ssDNA prevalence, RAD51 focus formation, and replication fork and genome stability following exposure to MMC. Interestingly, depletion of CHD4, but not CHD1, reduced the elevated RPA focus formation observed upon SETD1A loss (Figures S5A and S5B). However, abrogation of CHD4 expression in the absence of SETD1A did not restore RAD51 focus formation (Figure 6A), suggesting that CHD4 does not act to destabilize RAD51 nucleofilaments in the absence of H3K4 methylation. Nevertheless, depletion of CHD4 in cells lacking SETD1A restored replication fork stability (Figures 6B and 6C), rescued MMC hyper-sensitivity (Figure S5C), and reduced MMC-induced genome instability (Figure 6D), consistent with a role for CHD4 in promoting nascent strand degradation in the absence of SETD1A. In keeping with the known role for H3K4 methylation in negatively regulating CHD4, loss of SETD1A increased CHD4 localization on nascent DNA upon HU treatment (Figure 6E), despite having no effect on H3K9me3 levels at these sites (Figure S5D). In further support, depletion of CHD4 (but not CHD1) from cells expressing H3.1-GFP K4A also restored fork stability to normal levels (Figure S5E). Together, these findings demonstrate that CHD4-mediated fork degradation underlies the genome instability arising in cells lacking SETD1A and that H3K4 methylation protects genome stability in part by restricting the accessibility of CHD4 to reversed forks.

The Histone Chaperone Activity of FANCD2 Acts Downstream of Histone Methylation to Protect Stalled Forks

It is well-established that FANCD2 plays multiple roles in protecting against replication stress, including the ability to suppress DNA2-dependent degradation of nascent DNA in the absence of BRCA1/2 (Kais et al., 2016; Karanja et al., 2014; Schlacher et al., 2012). Given the functional overlap with SETD1A, and because we have previously demonstrated that FANCD2 interacts with BOD1L (Higgs et al., 2015), we postulated that BOD1L, SETD1A, and FANCD2 may function together. In agreement, co-depletion of FANCD2 and SETD1A had no additional effect on the extent of fork resection compared with loss of the individual genes alone (Figure 7A; Table S1), and depletion of BLM alleviated the fork resection observed in cells lacking FANCD2 (Figure S6A; Table S1). Furthermore, loss of FANCD2 did not further increase nascent DNA degradation in cells expressing GFP-H3.1 K4A (Figure 7B). Together, this provides strong evidence that SETD1A, H3K4 methylation, and FANCD2 function within the same pathway to prevent deleterious resection of stalled forks.

FANCD2 also possesses a histone chaperone activity that is critical for ICL repair *in vitro* and *in vivo* (Sato et al., 2012). Given the links between SETD1A, H3 methylation, and FANCD2, we postulated that the BOD1L/SETD1A complex may also be required for histone chaperoning upon replication stress. To assess this, we depleted BOD1L, SETD1A, or SETD1B from cells expressing WT H3.1-GFP and analyzed the mobility of GFP-tagged H3.1 before and after MMC exposure using fluorescence recovery after photobleaching (FRAP). Previous data demonstrated that, in the absence of FANCD2, the recovery kinetics of H3.1-GFP were perturbed specifically in the presence of replication stress (Sato et al., 2012). Strikingly, the mobility of H3.1-GFP after MMC treatment was also impaired in the absence of SETD1A or BOD1L (but not SETD1B) (Figure S6B) in a manner similar to cells lacking FANCD2. Furthermore, co-depletion of FANCD2 alongside either BOD1L or SETD1A had no significant additional effect on H3.1-GFP mobility (Figures S6C and S6D), suggesting that these three proteins function together to remodel chromatin after replication stress. To assess whether SETD1A and FANCD2 were specifically required for the mobility of newly synthesized histones, we next made use of the SNAP-tagged H3.1 system (Adam et al., 2013). These analyses revealed that SETD1A and FANCD2 also promote the mobility or deposition of new H3.1 histones after HU exposure (Figures 7C and S6E).

Given that loss of BOD1L/SETD1A perturbs histone mobility, we postulated that impaired H3K4me may also negatively affect this process. We therefore analyzed histone mobility by FRAP in cells expressing the H3.1-GFP K4A variant. When compared with WT H3.1-GFP, mutation of Lys4 lead to impaired H3.1-GFP mobility specifically after replication stress (Figures 7D and S6F), a finding recapitulated in both cell clones (Figure S6G). Together, these data suggest that H3K4 methylation promotes H3 mobility in the presence of replication damage. In agreement, depletion of either BOD1L or SETD1A had no additional effect on H3.1-GFP K4A mobility (Figure S6H), indicating that this KMT complex promotes histone mobility through its ability to methylate H3K4.

(D) Stable HeLa cells expressing WT H3.1-GFP or a K4A mutant (clone D1) were treated as described in Figure 3, and average IdU:CldU ratios were calculated (denoted by an arrow).
(E) Cells from (D) were transfected with the indicated siRNAs and treated as above.
(F) Cells from (D) were exposed to 50 ng/mL MMC for 24 hr or 4 mM HU for 5 hr and immunostained with antibodies to RAD51, and focus formation was analyzed by fluorescence microscopy. Scale bar, 20 μm.
(G) Quantification of PLA signals between EdU and RAD51 in cells from (D). Where denoted, cells were exposed to 4 mM HU for 5 hr.
(H) Cells from (D) were exposed to the indicated doses of MMC and left for 14 days, and colonies were stained with methylene blue and enumerated.
(I) Cells from (D) were exposed to 50 ng/mL MMC for 20 hr and treated with colcemid, and the incidence of chromosomal damage was analyzed by Giemsa staining and light microscopy.
The plots in all cases represent mean data ± SEM from 3 independent experiments. *p < 0.05, **p < 0.01, and ***p < 0.001. See also Figure S4 and Table S1.

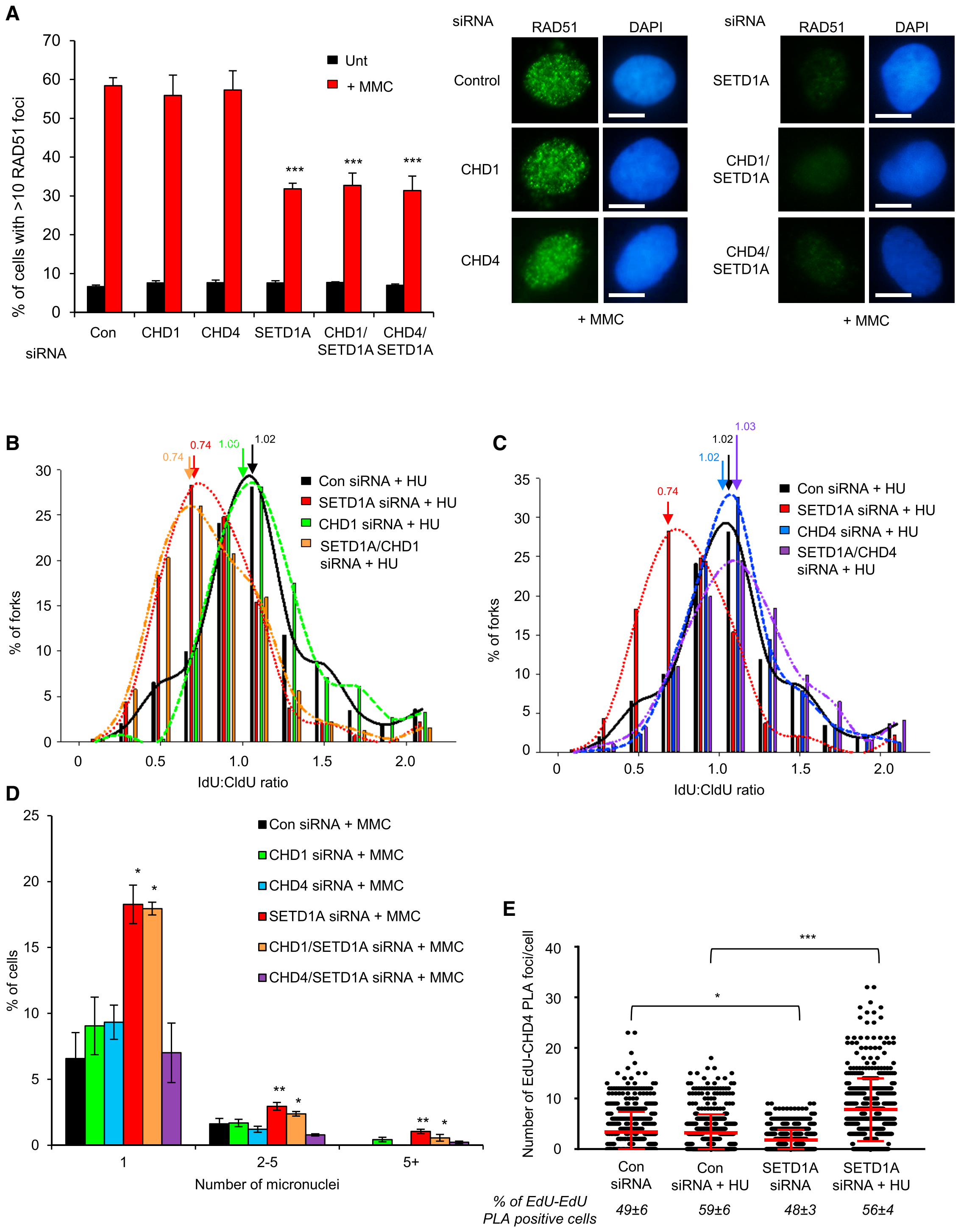

Figure 6. H3K4 Methylation by SETD1A Protects against CHD4-Mediated Fork Degradation

(A) U-2-OS cells were transfected with the indicated siRNAs for 72 hr, exposed to 50 ng/mL MMC for 24 hr, and immunostained with antibodies to RAD51, and focus formation was analyzed by fluorescence microscopy. Representative images are shown (right). Scale bars, 10 μm.

(B and C) Cells were transfected with control siRNA or those targeting SETD1A and CHD1 (A) or SETD1A and CHD4 (B), exposed to HU, and treated as described in Figure 3. Plots in (B) and (C) amalgamate data from the same experiments; arrows indicate mean ratios.

(legend continued on next page)

Intriguingly, these data also suggest that stalled replication forks may be protected from degradation by the chaperone activity of FANCD2. To address this possibility, we made use of DT40 cells expressing either WT chFANCD2, the mono-ubiquitylation-deficient chFANCD2-K563R mutant, or the histone chaperone-defective mutant chFANCD2-R305W (Sato et al., 2012; Figure S7A). We then compared the ability of these variants to prevent fork degradation after prolonged HU treatment. Notably, loss of the histone chaperone function of FANCD2 compromised its ability to protect nascent DNA from processing (Figure 7E; Table S1). Moreover, pharmacological inhibition of DNA2 (Liu et al., 2016), but not MRE11, in cells expressing chFANCD2-R305W restored fork stability (Table S1), suggesting that the histone chaperone function of FANCD2 protects against DNA2-dependent fork degradation. Finally, and in keeping with a role for the histone chaperone activity of FANCD2 in promoting RAD51-dependent fork protection, the destabilization of MMC-induced RAD51 nucleofilaments in human cells lacking FANCD2 (measured by FRAP) (Sato et al., 2016) was not restored by expression of the histone chaperone-defective R302W mutant (Figures 7F and S7B).

To further delineate the link between the histone chaperone activity of FANCD2 and H3K4 methylation, we examined whether binding of FANCD2 to H3 was affected by H3K4 methylation or whether FANCD2 was necessary for SETD1A activity. Interestingly, although loss of FANCD2 expression had no effect on H3K4me1 levels (Figure S7C), we observed a small but reproducible increase in the binding of FANCD2 (either from extracts or using recombinant protein) to H3 peptides or proteins that were mono-methylated on K4 (Figures S7D–S7G), suggesting that H3K4me1 may modulate FANCD2 binding, albeit mildly. In agreement, loss of SETD1A had a mild effect on the recruitment of FANCD2 to damaged chromatin (Figure S7H), but not to nascent DNA (Figure S7I). Although we did not observe a marked effect of H3K4me1 on FANCD2-histone binding, our data suggest that this modification might, in part, facilitate recruitment of FANCD2 to sites of replication stress.

Combined, our data demonstrate that, during replication stress, H3K4 methylation by SETD1A protects replication forks from over-resection by limiting CHD4 localization and by enhancing FANCD2-dependent histone chaperone activity. Subsequent histone mobility protects stalled replication forks from degradation by promoting RAD51-dependent fork protection (Figure 7G). In the absence of H3K4 methylation or SETD1A, histone mobility is reduced, destabilizing RAD51 at damaged forks. These forks then undergo deleterious resection, partly mediated via the unrestrained activity of CHD4, giving rise to severe genome instability.

DISCUSSION

Although many factors required to stabilize damaged replication forks have been identified, it remains to be determined how post-translational modification of chromatin helps to govern this process. Here we have demonstrated that the KMT SETD1A plays an integral role in preventing degradation of nascent DNA at reversed forks. Critically, we have shown that SETD1A-mediated H3K4 methylation is required for this protection and for the stabilization of RAD51 at reversed forks. As a consequence, cells depleted of SETD1A or expressing a mutant of H3 unable to be methylated on Lys4, fail to stabilize RAD51, are unable to protect nascent DNA from degradation by DNA2, and exhibit increased genome instability after exposure to replication stress. Moreover, because the fork degradation in cells lacking SETD1A or expressing H3K4A can be rescued by depletion of the anti-recombinase BLM, this strongly suggests that H3K4 methylation is inherently linked to maintaining RAD51 nucleofilament stability.

SETD1A and SETD1B Are Functionally Distinct KMTs

Although the COMPASS KMT Set1 and histone H3K4 methylation have previously been linked to resistance to replication stress in yeast (Faucher and Wellinger, 2010), the roles of the mammalian COMPASS homologs SETD1A and SETD1B in this process have so far not been examined. Here we demonstrate that, although these two KMTs both target H3K4 for methylation, only SETD1A is required to protect cells against replication stress. Indeed, in contrast to loss of SETD1A, depletion of SETD1B does not confer MMC hyper-sensitivity at the cellular level and does not lead to replication fork instability (Figures 1, 2, and 3). This functional divergence is likely linked to their ability to bind specific Shg1-like proteins, our data (Figures 1 and S1), combined with previous analyses, strongly suggest that SETD1A functionally interacts with BOD1L, whereas SETD1B interacts with BOD1 (Hein et al., 2015; van Nuland et al., 2013). Moreover, BOD1, like SETD1B, plays no discernible role in the cellular response to replication stress (Higgs et al., 2015). This is in agreement with a recent publication describing a functional cytoplasmic SETD1B-BOD1 complex (Wang et al., 2017). Interestingly, because BOD1 modulates the activity of specific PP2A regulatory subunits at kinetochores during mitosis (Porter et al., 2007; Porter et al., 2013), SETD1B-dependent histone methylation may also play a role in this process.

We speculate that, because both BOD1 and BOD1L contain a COMPASS-Shg1 domain (Marchler-Bauer et al., 2011; van Nuland et al., 2013), which, in the case of BOD1L, likely mediates its interaction with SETD1A (Figure S1B), it is these subunits of the SET1-containing complexes that regulate the distribution and/or function of the respective catalytic KMT. Because BOD1L is a target for ataxia telangiectasia mutated/ATR-dependent (ATM/ATR-dependent) phosphorylation (Matsuoka et al., 2007), we hypothesize that such damage-induced modification may modulate the recruitment and/or activity of the BOD1L/SETD1A complex at stalled replication forks. In addition, our data suggest that, despite their shared ability to methylate H3K4, SETD1A and SETD1B may methylate chromatin at distinct

(D) Micronucleus formation in cells from (A) was analyzed by fluorescence microscopy.

(E) Quantification of PLA signals between EdU and RAD51 in U-2-OS cells transfected with the indicated siRNA. Where denoted, cells were exposed to 4 mM HU for 5 hr.

In all cases, plots represent mean data ± SEM from 3 independent experiments. *$p < 0.05$ and ***$p < 0.001$. See also Figure S5 and Table S1.

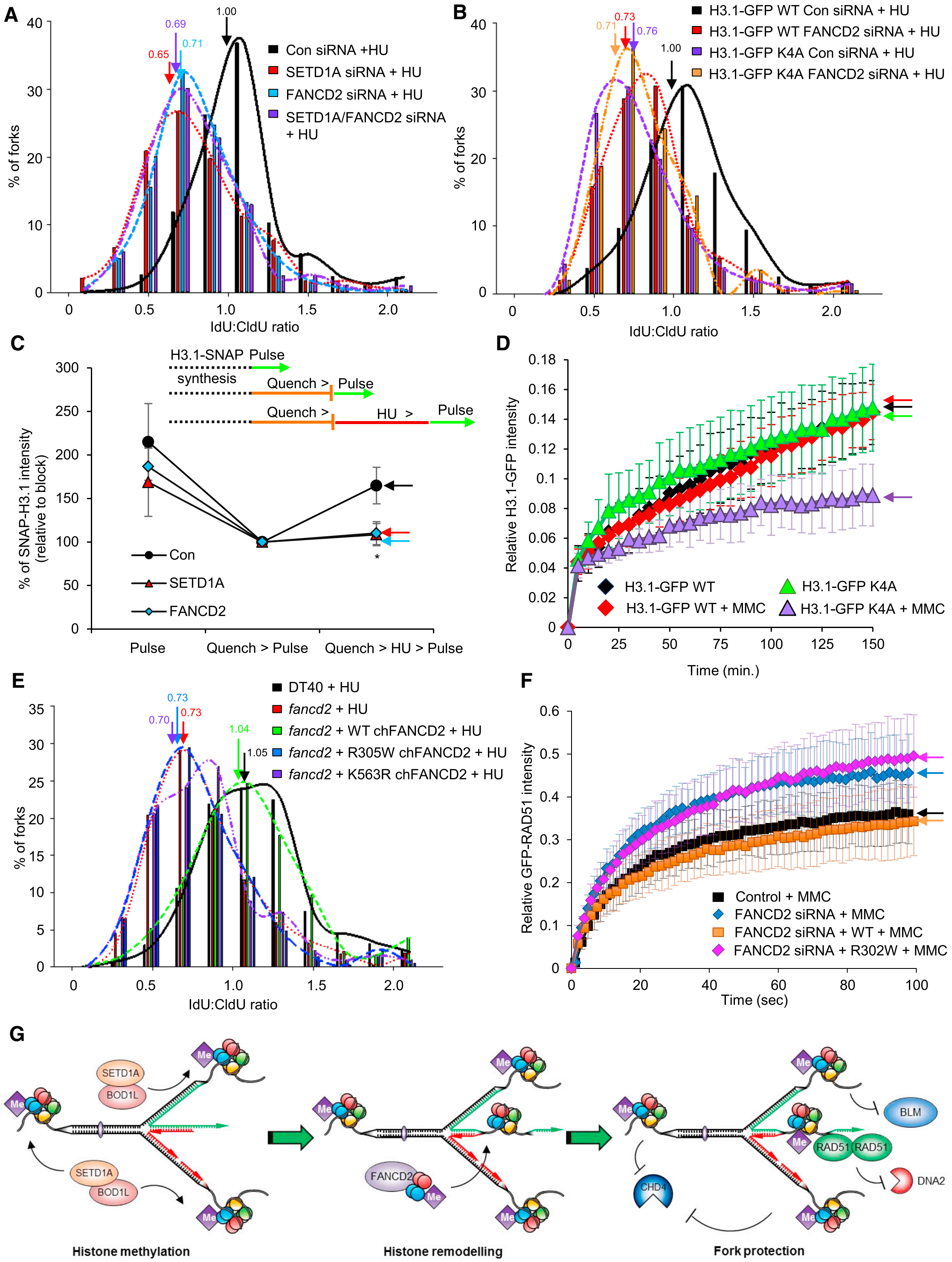

Figure 7. SETD1A and FANCD2 Promote H3 Mobility to Protect Fork Integrity

(A) U-2-OS cells were transfected with the indicated siRNAs and treated as in Figure 3. Arrows indicate mean ratios.

(B) Stable HeLa cells expressing WT H3.1-GFP or a K4A mutant (clone D1) were transfected with the indicated siRNAs and treated as above.

(C) U-2-OS cells from (A) were transfected with SNAP-tagged H3.1 and analyzed to reveal levels of pre-existing SNAP-H3.1 (Pulse), background fluorescence (Quench-Pulse), and new H3.1 after a 2-hr release into HU (Quench-HU-Pulse). Cells were analyzed by fluorescence microscopy, and fluorescence intensity per nucleus was quantified using ImageJ.

(legend continued on next page)

environments or in response to different stimuli. In support of this notion, SETD1A and SETD1B function in a non-redundant manner during embryogenesis and the embryonic lethality resulting from loss of either KMT occur at different stages of development (Bledau et al., 2014).

Mechanisms for Epigenetic Modifications in Regulating Fork Stability

Our data, when combined with observations assessing the effect of the H3K27 KMT EZH2 (Rondinelli et al., 2017) and the PTIP-containing MLL2/3 KMT complex (Ray Chaudhuri et al., 2016) on replication fork stability, are consistent with the hypothesis that epigenetic modifications control the recruitment and/or activity of factors that regulate fork stability. In keeping with this prediction, we demonstrate that H3K4 methylation regulates the localization of the chromodomain helicase DNA-binding protein CHD4 (Figure 6). Interestingly, our finding that CHD4 loss prevents nascent DNA degradation in the absence of SETD1A without restoring RAD51 recruitment (Figures 6A–6C) in a manner similar to observations in BRCA2 mutant cells (Guillemette et al., 2015; Ray Chaudhuri et al., 2016) suggests that CHD4 may either promote replication fork reversal or modulate the local chromatin environment to allow nucleases access to the damaged fork. Although the precise contribution of CHD4 to fork degradation in the context of SETD1A deficiency remains to be elucidated, in cells lacking BRCA1/2, this pro-resection activity of CHD4 occurs through dysregulation of RAD18-dependent translesion synthesis (TLS). It is currently unclear whether a similar mechanism occurs in BOD1L/SETD1A-depleted cells.

In addition to SETD1A and SETD1B, the KMT2 family of H3K4 methyltransferases also contains the myeloid/lymphoid (MLL) subfamily of enzymes (KMT2A–KMT2D). Recent studies have proposed a role for murine Mll3 and Mll4 (human KMT2C [MLL3] and KMT2D [MLL2] respectively) in promoting fork resection in the absence of BRCA2 (Ray Chaudhuri et al., 2016). These studies suggested that H3K4 methylation promotes fork degradation upon loss of BRCA1 or BRCA2 by recruiting MRE11 to stalled or reversed forks. In contrast, we have demonstrated that SETD1A-dependent H3K4 methylation protects reversed forks from resection by promoting RAD51-dependent fork stability. It is also clear that SETD1A depletion does not promote nascent strand degradation in the absence of BRCA2 and that it is functionally distinct from KMT2C/D (Figure 3). On face value, it is difficult to reconcile why different KMT complexes targeting H3K4 would have opposing roles at damaged replication forks. However, it is apparent that all 6 members of the KMT2 family of methyltransferases are not functionally redundant (Bledau et al., 2014; Duncan et al., 2015). Moreover, the timing, localization, and cellular context of specific nucleosomes being targeted for H3K4 methylation is likely to have very different outcomes. Interestingly, only loss of SETD1A (and not SETD1B or KMT2C/D) on a WT genetic background allows increased fork resection (Figure 3), indicating a primary constitutive role for this KMT2 family member in preventing fork degradation. In contrast, the enzymatic activity of the PTIP-associated methyltransferases (KMT2C/D) only appears to be required when fork stability has already been compromised; e.g., in the absence of BRCA2 (Ray Chaudhuri et al., 2016; Figure 3).

Several hypotheses arise from these observations: first, SETD1A may regulate multiple different processes required to stabilize a damaged fork; for example, stabilizing RAD51 nucleofilaments, suppressing anti-recombinase activity, and methylating H3 at reversed forks. Second, the context of the damaged fork might dictate which KMT is utilized to modify the nascent chromatin surrounding the fork. In this respect, it is conceivable that SETD1A and KMT2C/D could target histone H3 within different regions of the genome undergoing DNA replication (Calo and Wysocka, 2013; Duncan et al., 2015) or differentially methylate the histone variants H3.1 and H3.3 at damaged forks, depending on the composition of the chromatin undergoing replication; i.e., whether it is a transcriptionally active or inactive region. Finally, specific epigenetic changes surrounding damaged forks may dictate which KMT is recruited. For example, H2B mono-ubiquitylation in yeast and humans by the Bre1 or RNF20/40 ubiquitin ligases potentiates H3K4 methylation by promoting recruitment of the Set1-COMPASS complexes via their Swd2 subunit (human WDR82) (Hung et al., 2017; Wood et al., 2005; Wu et al., 2008). Because the MLL KMT complexes lack WDR82 (van Nuland et al., 2013), H2B mono-ubiquitylation would provide a high degree of specificity and selectivity between the different KMT2 complexes. Furthermore, because the KMT2 complexes differ in their associated cofactors, it is plausible that these complex-specific subunits (e.g., BOD1L and PTIP) may bind alternative epigenetic marks to convey further selectivity.

Methylation-Enhanced Nucleosome Mobility Is Required for Fork Protection

For the first time, our findings illustrate that histone methylation is intimately linked to nucleosome mobility at damaged forks and that this is required to prevent degradation of nascent DNA. At present, it is unclear how H3K4 methylation regulates FANCD2-dependent histone chaperoning. It is possible that SETD1A-dependent histone methylation may modulate the

(D) Stable HeLa cells expressing WT H3.1-GFP or a K4A mutant (clone D1) were left untreated or exposed to MMC for 24 hr and analyzed by FRAP.

(E) WT DT40 cells or *FANCD2*$^{-/-}$ cells expressing the indicated GFP-tagged chFANCD2 variants were treated as described in Figure 3. Arrows indicate mean ratios.

(F) Stable U-2-OS-GFP-RAD51 cells were co-transfected with the indicated siRNA and plasmids encoding either WT or chaperone-dead (R302W) mCherry-FANCD2 and exposed to MMC, and the mobility of GFP-RAD51 was analyzed by FRAP.

(G) Model for the role of SET1A-dependent H3K4 methylation in protecting stalled replication forks. Upon fork reversal during replication stress, H3K4me1 by SETD1A acts to restrict CHD4 localization and promote FANCD2-dependent histone mobility. This chaperone activity is vital to stabilize RAD51 nucleofilaments, preventing nucleolytic degradation of stalled forks by DNA2.

The plots in (D) and (F) represent mean ± SD relative fluorescence intensities from n = 21–26 and 25–48 cells, respectively. *$p < 0.05$. See also Figures S6 and S7 and Table S1.

accessibility of chromatin to FANCD2, allowing histones to be mobilized. Alternatively, H3K4 methylation may promote the recruitment and/or binding of co-factors vital to promote FANCD2-dependent nucleosome remodeling. Nevertheless, our working model places SETD1A-dependent H3K4 methylation as a major regulatory epigenetic modification that permits FANCD2 to mobilize histones onto the regressed arm of a reversed fork (Figure 7G), protecting the 3′ end of the RAD51 nucleofilament from the anti-recombinase activities of BLM. This is entirely in keeping with the finding that fork degradation in cells lacking FANCD2 is restored by co-depletion of BLM and that the histone chaperone activity of FANCD2 is required for RAD51 nucleofilament stability (Figures 7 and S7). Furthermore, this model predicts that movement of histones onto or near reversed forks is a vital mechanism to protect their integrity and suggests that other factors able to promote histone mobility in similar situations may also be involved in protecting nascent DNA.

In summary, our findings reveal the importance of the methylation and subsequent mobility of histones in protecting nascent DNA from nucleolytic degradation. Our data further reinforce the notion that epigenetic modifications of chromatin surrounding stalled forks play a critical role in governing the recruitment/activity of pro- and anti-resection factors and highlight the importance of context-dependent histone lysine methylation in maintaining genome stability.

STAR★METHODS

Detailed methods are provided in the online version of this paper and include the following:

- KEY RESOURCES TABLE
- CONTACT FOR REAGENT AND RESOURCE SHARING
- EXPERIMENTAL MODEL AND SUBJECT DETAILS
  - Cell Culture and Generation of Cell Lines
- METHOD DETAILS
  - siRNA Transfections
  - Drugs and Inhibitors
  - Cloning
  - Colony Survival Assays
  - DNA Fibers
  - iPOND
  - Proximity Ligation Assay (PLA)
  - Flow Cytometry
  - Metaphase Spreads
  - Immunofluorescence, Microscopy, and Image Analysis
  - SNAP Labeling of Histones
  - Fluorescence Recovery after Photobleaching (FRAP)
  - Western Blotting, Pull-downs, and Immunoprecipitations
- QUANTIFICATION AND STATISTICAL ANALYSIS

SUPPLEMENTAL INFORMATION

Supplemental Information includes seven figures and one table and can be found with this article online at https://doi.org/10.1016/j.molcel.2018.05.018.

ACKNOWLEDGMENTS

We thank members of the Birmingham Centre for Genome Biology (BCGB), in particular Marco Saponaro, Aga Gambus, and A. Malcolm Taylor, for invaluable discussions. We are grateful to David Cortez for providing iPOND mass spectrometry data and to Judith Campbell for the DNA2 inhibitor. We also thank Simon Boulton, Claus Storgaard Sørensen, Martin Cohn, Raymund Wellinger, and Francis Stewart for reagents and advice. M.R.H., S.B., and A.G. are funded by an MRC career development fellowship (to M.R.H., MR/P009085/1). R.B. and M.R.H. are funded by a Birmingham fellowship awarded to M.R.H. by the University of Birmingham. A.V. and G.S.S. are funded by a CR-UK senior fellowship (C17183/A13030) and a CR-UK program grant (C17183/A23303). J.J.R. was funded by an MRC project grant (MR/M009882/1) and is now supported by the University of Birmingham. This work was supported in part by JSPS KAKENHI grants JP25116002 and JP17H01408 (to H. Kurumizaka) and JP25116005 (to H. Kimura). D.G.S. is funded by the IUPUI School of Science.

AUTHOR CONTRIBUTIONS

Conceptualization, M.R.H., J.J.R., and G.S.S.; Methodology, M.R.H. and G.S.S.; Investigation, M.R.H., G.S.S., J.J.R., R.B., S.B., A.G., A.V., K.L.P., K.S., H. Kurumizaka, and H. Kimura; Writing – Original Draft, M.R.H. and G.S.S.; Writing – Review & Editing, M.R.H., J.J.R., and G.S.S.; Funding Acquisition, M.R.H. and G.S.S.; Resources, N.G.H., K.L.P., D.G.S., W.K., and M.T.; Supervision, M.R.H. and G.S.S.

DECLARATION OF INTERESTS

The authors declare no competing interests.

Received: January 22, 2018
Revised: April 20, 2018
Accepted: May 15, 2018
Published: June 21, 2018

REFERENCES

Adam, S., Polo, S.E., and Almouzni, G. (2013). Transcription recovery after DNA damage requires chromatin priming by the H3.3 histone chaperone HIRA. Cell *155*, 94–106.

Arndt, K., Kranz, A., Fohgrub, J., Jolly, A., Bledau, A.S., Di Virgilio, M., Lesche, M., Dahl, A., Höfer, T., Stewart, A.F., and Waskow, C. (2018). SETD1A protects HSCs from activation-induced functional decline in vivo. Blood *131*, 1311–1324.

Bledau, A.S., Schmidt, K., Neumann, K., Hill, U., Ciotta, G., Gupta, A., Torres, D.C., Fu, J., Kranz, A., Stewart, A.F., and Anastassiadis, K. (2014). The H3K4 methyltransferase Setd1a is first required at the epiblast stage, whereas Setd1b becomes essential after gastrulation. Development *141*, 1022–1035.

Brici, D., Zhang, Q., Reinhardt, S., Dahl, A., Hartmann, H., Schmidt, K., Goveas, N., Huang, J., Gahurova, L., Kelsey, G., et al. (2017). *Setd1b*, encoding a histone 3 lysine 4 methyltransferase, is a maternal effect gene required for the oogenic gene expression program. Development *144*, 2606–2617.

Calo, E., and Wysocka, J. (2013). Modification of enhancer chromatin: what, how, and why? Mol. Cell *49*, 825–837.

Duncan, E.M., Chitsazan, A.D., Seidel, C.W., and Sánchez Alvarado, A. (2015). Set1 and MLL1/2 target distinct sets of functionally different genomic loci in vivo. Cell Rep. *13*, 2741–2755.

Dungrawala, H., Rose, K.L., Bhat, K.P., Mohni, K.N., Glick, G.G., Couch, F.B., and Cortez, D. (2015). The replication checkpoint prevents two types of fork collapse without regulating replisome stability. Mol. Cell *59*, 998–1010.

Faucher, D., and Wellinger, R.J. (2010). Methylated H3K4, a transcription-associated histone modification, is involved in the DNA damage response pathway. PLoS Genet. *6*, e1001082.

Guillemette, S., Serra, R.W., Peng, M., Hayes, J.A., Konstantinopoulos, P.A., Green, M.R., and Cantor, S.B. (2015). Resistance to therapy in BRCA2 mutant

cells due to loss of the nucleosome remodeling factor CHD4. Genes Dev. *29*, 489–494.

Hein, M.Y., Hubner, N.C., Poser, I., Cox, J., Nagaraj, N., Toyoda, Y., Gak, I.A., Weisswange, I., Mansfeld, J., Buchholz, F., et al. (2015). A human interactome in three quantitative dimensions organized by stoichiometries and abundances. Cell *163*, 712–723.

Higgs, M.R., and Stewart, G.S. (2016). Protection or resection: BOD1L as a novel replication fork protection factor. Nucleus *7*, 34–40.

Higgs, M.R., Reynolds, J.J., Winczura, A., Blackford, A.N., Borel, V., Miller, E.S., Zlatanou, A., Nieminuszczy, J., Ryan, E.L., Davies, N.J., et al. (2015). BOD1L is required to suppress deleterious resection of stressed replication forks. Mol. Cell *59*, 462–477.

Hoshii, T., Cifani, P., Feng, Z., Huang, C.H., Koche, R., Chen, C.W., Delaney, C.D., Lowe, S.W., Kentsis, A., and Armstrong, S.A. (2018). A Non-catalytic function of SETD1A regulates cyclin K and the DNA damage response. Cell *172*, 1007–1021.e17.

Hung, S.H., Wong, R.P., Ulrich, H.D., and Kao, C.F. (2017). Monoubiquitylation of histone H2B contributes to the bypass of DNA damage during and after DNA replication. Proc. Natl. Acad. Sci. USA *114*, E2205–E2214.

Kais, Z., Rondinelli, B., Holmes, A., O'Leary, C., Kozono, D., D'Andrea, A.D., and Ceccaldi, R. (2016). FANCD2 maintains fork stability in BRCA1/2-deficient tumors and promotes alternative end-joining DNA repair. Cell Rep. *15*, 2488–2499.

Karanja, K.K., Lee, E.H., Hendrickson, E.A., and Campbell, J.L. (2014). Preventing over-resection by DNA2 helicase/nuclease suppresses repair defects in Fanconi anemia cells. Cell Cycle *13*, 1540–1550.

Kari, V., Mansour, W.Y., Raul, S.K., Baumgart, S.J., Mund, A., Grade, M., Sirma, H., Simon, R., Will, H., Dobbelstein, M., et al. (2016). Loss of CHD1 causes DNA repair defects and enhances prostate cancer therapeutic responsiveness. EMBO Rep. *17*, 1609–1623.

Kolinjivadi, A.M., Sannino, V., De Antoni, A., Zadorozhny, K., Kilkenny, M., Techer, H., Baldi, G., Shen, R., Ciccia, A., Pellegrini, L., et al. (2017). Smarcal1-mediated fork reversal triggers Mre11-dependent degradation of nascent DNA in the absence of Brca2 and stable Rad51 nucleofilaments. Mol. Cell *67*, 867–881.e7.

Lee, J.H., and Skalnik, D.G. (2005). CpG-binding protein (CXXC finger protein 1) is a component of the mammalian Set1 histone H3-Lys4 methyltransferase complex, the analogue of the yeast Set1/COMPASS complex. J. Biol. Chem. *280*, 41725–41731.

Lee, J.H., and Skalnik, D.G. (2008). Wdr82 is a C-terminal domain-binding protein that recruits the Setd1A Histone H3-Lys4 methyltransferase complex to transcription start sites of transcribed human genes. Mol. Cell. Biol. *28*, 609–618.

Lee, J.H., Tate, C.M., You, J.S., and Skalnik, D.G. (2007). Identification and characterization of the human Set1B histone H3-Lys4 methyltransferase complex. J. Biol. Chem. *282*, 13419–13428.

Leuzzi, G., Marabitti, V., Pichierri, P., and Franchitto, A. (2016). WRNIP1 protects stalled forks from degradation and promotes fork restart after replication stress. EMBO J. *35*, 1437–1451.

Liu, W., Zhou, M., Li, Z., Li, H., Polaczek, P., Dai, H., Wu, Q., Liu, C., Karanja, K.K., Popuri, V., et al. (2016). A selective small molecule DNA2 inhibitor for sensitization of human cancer cells to chemotherapy. EBioMedicine *6*, 73–86.

Luciano, P., Jeon, J., El-Kaoutari, A., Challal, D., Bonnet, A., Barucco, M., Candelli, T., Jourquin, F., Lesage, P., Kim, J., et al. (2017). Binding to RNA regulates Set1 function. Cell Discov. *3*, 17040.

Marchler-Bauer, A., Lu, S., Anderson, J.B., Chitsaz, F., Derbyshire, M.K., DeWeese-Scott, C., Fong, J.H., Geer, L.Y., Geer, R.C., Gonzales, N.R., et al. (2011). CDD: a Conserved Domain Database for the functional annotation of proteins. Nucleic Acids Res. *39*, D225–D229.

Matsuoka, S., Ballif, B.A., Smogorzewska, A., McDonald, E.R., 3rd, Hurov, K.E., Luo, J., Bakalarski, C.E., Zhao, Z., Solimini, N., Lerenthal, Y., et al. (2007). ATM and ATR substrate analysis reveals extensive protein networks responsive to DNA damage. Science *316*, 1160–1166.

Mijic, S., Zellweger, R., Chappidi, N., Berti, M., Jacobs, K., Mutreja, K., Ursich, S., Ray Chaudhuri, A., Nussenzweig, A., Janscak, P., and Lopes, M. (2017). Replication fork reversal triggers fork degradation in BRCA2-defective cells. Nat. Commun. *8*, 859.

Mochizuki, A.L., Katanaya, A., Hayashi, E., Hosokawa, M., Moribe, E., Motegi, A., Ishiai, M., Takata, M., Kondoh, G., Watanabe, H., et al. (2017). PARI regulates stalled replication fork processing to maintain genome stability upon replication stress in mice. Mol. Cell. Biol. *37*, e00117-17.

Neelsen, K.J., and Lopes, M. (2015). Replication fork reversal in eukaryotes: from dead end to dynamic response. Nat. Rev. Mol. Cell Biol. *16*, 207–220.

Petruk, S., Sedkov, Y., Johnston, D.M., Hodgson, J.W., Black, K.L., Kovermann, S.K., Beck, S., Canaani, E., Brock, H.W., and Mazo, A. (2012). TrxG and PcG proteins but not methylated histones remain associated with DNA through replication. Cell *150*, 922–933.

Porter, I.M., McClelland, S.E., Khoudoli, G.A., Hunter, C.J., Andersen, J.S., McAinsh, A.D., Blow, J.J., and Swedlow, J.R. (2007). Bod1, a novel kinetochore protein required for chromosome biorientation. J. Cell Biol. *179*, 187–197.

Porter, I.M., Schleicher, K., Porter, M., and Swedlow, J.R. (2013). Bod1 regulates protein phosphatase 2A at mitotic kinetochores. Nat. Commun. *4*, 2677.

Quinet, A., Lemaçon, D., and Vindigni, A. (2017). Replication fork reversal: players and guardians. Mol. Cell *68*, 830–833.

Ray Chaudhuri, A., Callen, E., Ding, X., Gogola, E., Duarte, A.A., Lee, J.E., Wong, N., Lafarga, V., Calvo, J.A., Panzarino, N.J., et al. (2016). Replication fork stability confers chemoresistance in BRCA-deficient cells. Nature *535*, 382–387.

Roguev, A., Schaft, D., Shevchenko, A., Pijnappel, W.W., Wilm, M., Aasland, R., and Stewart, A.F. (2001). The Saccharomyces cerevisiae Set1 complex includes an Ash2 homologue and methylates histone 3 lysine 4. EMBO J. *20*, 7137–7148.

Rondinelli, B., Gogola, E., Yücel, H., Duarte, A.A., van de Ven, M., van der Sluijs, R., Konstantinopoulos, P.A., Jonkers, J., Ceccaldi, R., Rottenberg, S., and D'Andrea, A.D. (2017). EZH2 promotes degradation of stalled replication forks by recruiting MUS81 through histone H3 trimethylation. Nat. Cell Biol. *19*, 1371–1378.

Sato, K., Ishiai, M., Toda, K., Furukoshi, S., Osakabe, A., Tachiwana, H., Takizawa, Y., Kagawa, W., Kitao, H., Dohmae, N., et al. (2012). Histone chaperone activity of Fanconi anemia proteins, FANCD2 and FANCI, is required for DNA crosslink repair. EMBO J. *31*, 3524–3536.

Sato, K., Shimomuki, M., Katsuki, Y., Takahashi, D., Kobayashi, W., Ishiai, M., Miyoshi, H., Takata, M., and Kurumizaka, H. (2016). FANCI-FANCD2 stabilizes the RAD51-DNA complex by binding RAD51 and protects the 5′-DNA end. Nucleic Acids Res. *44*, 10758–10771.

Schlacher, K., Christ, N., Siaud, N., Egashira, A., Wu, H., and Jasin, M. (2011). Double-strand break repair-independent role for BRCA2 in blocking stalled replication fork degradation by MRE11. Cell *145*, 529–542.

Schlacher, K., Wu, H., and Jasin, M. (2012). A distinct replication fork protection pathway connects Fanconi anemia tumor suppressors to RAD51-BRCA1/2. Cancer Cell *22*, 106–116.

Schlichter, A., and Cairns, B.R. (2005). Histone trimethylation by Set1 is coordinated by the RRM, autoinhibitory, and catalytic domains. EMBO J. *24*, 1222–1231.

Shenoy, T.R., Boysen, G., Wang, M.Y., Xu, Q.Z., Guo, W., Koh, F.M., Wang, C., Zhang, L.Z., Wang, Y., Gil, V., et al. (2017). CHD1 loss sensitizes prostate cancer to DNA damaging therapy by promoting error-prone double-strand break repair. Ann. Oncol. *28*, 1495–1507.

Sims, R.J., 3rd, Chen, C.F., Santos-Rosa, H., Kouzarides, T., Patel, S.S., and Reinberg, D. (2005). Human but not yeast CHD1 binds directly and selectively to histone H3 methylated at lysine 4 via its tandem chromodomains. J. Biol. Chem. *280*, 41789–41792.

Somyajit, K., Saxena, S., Babu, S., Mishra, A., and Nagaraju, G. (2015). Mammalian RAD51 paralogs protect nascent DNA at stalled forks and mediate replication restart. Nucleic Acids Res. *43*, 9835–9855.

Taglialatela, A., Alvarez, S., Leuzzi, G., Sannino, V., Ranjha, L., Huang, J.W., Madubata, C., Anand, R., Levy, B., Rabadan, R., et al. (2017). Restoration of replication fork stability in BRCA1- and BRCA2-deficient cells by inactivation of SNF2-family fork remodelers. Mol. Cell *68*, 414–430.e8.

Takahashi, D., Sato, K., Shimomuki, M., Takata, M., and Kurumizaka, H. (2014). Expression and purification of human FANCI and FANCD2 using Escherichia coli cells. Protein Expr. Purif. *103*, 8–15.

Thangavel, S., Berti, M., Levikova, M., Pinto, C., Gomathinayagam, S., Vujanovic, M., Zellweger, R., Moore, H., Lee, E.H., Hendrickson, E.A., et al. (2015). DNA2 drives processing and restart of reversed replication forks in human cells. J. Cell Biol. *208*, 545–562.

van Nuland, R., Smits, A.H., Pallaki, P., Jansen, P.W., Vermeulen, M., and Timmers, H.T. (2013). Quantitative dissection and stoichiometry determination of the human SET1/MLL histone methyltransferase complexes. Mol. Cell. Biol. *33*, 2067–2077.

Wang, A.T., Kim, T., Wagner, J.E., Conti, B.A., Lach, F.P., Huang, A.L., Molina, H., Sanborn, E.M., Zierhut, H., Cornes, B.K., et al. (2015). A dominant mutation in human RAD51 reveals its function in DNA interstrand crosslink repair independent of homologous recombination. Mol. Cell *59*, 478–490.

Wang, L., Collings, C.K., Zhao, Z., Cozzolino, K.A., Ma, Q., Liang, K., Marshall, S.A., Sze, C.C., Hashizume, R., Savas, J.N., and Shilatifard, A. (2017). A cytoplasmic COMPASS is necessary for cell survival and triple-negative breast cancer pathogenesis by regulating metabolism. Genes Dev. *31*, 2056–2066.

Watson, A.A., Mahajan, P., Mertens, H.D., Deery, M.J., Zhang, W., Pham, P., Du, X., Bartke, T., Zhang, W., Edlich, C., et al. (2012). The PHD and chromo domains regulate the ATPase activity of the human chromatin remodeler CHD4. J. Mol. Biol. *422*, 3–17.

Wood, A., Schneider, J., Dover, J., Johnston, M., and Shilatifard, A. (2005). The Bur1/Bur2 complex is required for histone H2B monoubiquitination by Rad6/Bre1 and histone methylation by COMPASS. Mol. Cell *20*, 589–599.

Wu, M., Wang, P.F., Lee, J.S., Martin-Brown, S., Florens, L., Washburn, M., and Shilatifard, A. (2008). Molecular regulation of H3K4 trimethylation by Wdr82, a component of human Set1/COMPASS. Mol. Cell. Biol. *28*, 7337–7344.

Wysocka, J. (2006). Identifying novel proteins recognizing histone modifications using peptide pull-down assay. Methods *40*, 339–343.

Ying, S., Hamdy, F.C., and Helleday, T. (2012). Mre11-dependent degradation of stalled DNA replication forks is prevented by BRCA2 and PARP1. Cancer Res. *72*, 2814–2821.

Zadorozhny, K., Sannino, V., Beláň, O., Mlčoušková, J., Špírek, M., Costanzo, V., and Krejčí, L. (2017). Fanconi-anemia-associated mutations destabilize RAD51 filaments and impair replication fork protection. Cell Rep. *21*, 333–340.

Zellweger, R., Dalcher, D., Mutreja, K., Berti, M., Schmid, J.A., Herrador, R., Vindigni, A., and Lopes, M. (2015). Rad51-mediated replication fork reversal is a global response to genotoxic treatments in human cells. J. Cell Biol. *208*, 563–579.

STAR★METHODS

KEY RESOURCES TABLE

REAGENT or RESOURCE	SOURCE	IDENTIFIER
Antibodies		
BOD1L	Grant Stewart; Higgs et al., 2015	N/A
SETD1A	Bethyl Labs	Cat# A300-289A; RRID: AB_263413
SETD1B	Bethyl Labs	Cat# A302-281A; RRID: AB_1850180
BOD1	Jason Swedlow; Porter et al., 2013	N/A
H3 (detection of GFP-H3)	Active Motif	Cat# 39763; RRID: AB_2650522
H3	Abcam	Cat# ab1791; RRID: AB_302613
H3K4me1	Abcam	Cat# ab8895; RRID: AB_306847
H3K4me2	Millipore	Cat# 04-790; RRID: AB_10562969
H3K4me3	Abcam	Cat# ab8580; RRID: AB_306649
H3K9me3	Abcam	Cat# ab8898; RRID: AB_306848
RPA	Millipore	Cat# NA18; RRID: AB_10682810
P-RPA (S4/8)	Bethyl Labs	Cat# A300-245A; RRID: AB_210547
RAD51	Millipore	Cat# PC130; RRID: AB_2238184
γH2AX	Millipore	Cat# 05-636; RRID: AB_309864
H2A	Millipore	Cat# 07-146; RRID: AB_11212920
CldU (BrdU)	Bio-Rad	Cat# OBT0030G; RRID: AB_609567
IdU (BrdU)	Becton Dickinson	Cat# 347580; RRID: AB_10015219
FANCD2	Santa Cruz	Cat# sc-20022; RRID: AB_2278211
FANCD2	Novus	Cat# NB100-182; RRID: AB_10002867
Actin	Sigma-Aldrich	Cat# A5316; RRID: AB_476743
BLM	Bethyl Labs	Cat# A300-110A; RRID: AB_2064794
FLAG	Sigma-Aldrich	Cat# F1804; RRID: AB_262044
PCNA	Santa Cruz	Cat# sc-56; RRID: AB_628110
MRE11	Abcam	Cat# ab397; RRID: AB_2145241
GFP	Roche	Cat# 11814460001; RRID: AB_390913
CHD1	Bethyl Labs	Cat# A301-218A; RRID: AB_890568
CHD4	Bethyl Labs	Cat# A301-082A; RRID: AB_873002
Cyclin K	Bethyl Labs	Cat# A301-939A; RRID: AB_1547934
GST	Thermo Fisher Scientific	Cat# 700775; RRID: AB_2532343
Biotin (rabbit)	Bethyl Labs	Cat# A150-109A; RRID: AB_67327
Biotin (mouse)	Jackson ImmunoResearch	Cat# 200-002-211; RRID: AB_2339006
Alexa Fluor anti-mouse 594	Thermo Fisher Scientific	Cat# A11032; RRID: AB_141672
Alexa Fluor anti-rabbit 488	Thermo Fisher Scientific	Cat# A11070; RRID: AB_142134
Alexa Fluor anti-rat 555	Thermo Fisher Scientific	Cat# A21434; RRID: AB_141733
Alexa Fluor anti-mouse 488	Thermo Fisher Scientific	Cat# A11029; RRID: AB_138404
Anti-rabbit HRP	Agilent	Cat# P0399; RRID: AB_2617141
Anti-mouse HRP	Agilent	Cat# P0447; RRID: AB_2617137
Anti-goat HRP	Agilent	Cat# P0449; RRID: AB_2617143
Anti-rabbit HRP (Reliablot)	Bethyl Labs	Cat# WB120
IgG	Agilent	Cat# X0903
Bacterial and Virus Strains		
BL21-CodonPlus (DE3)-RP-X Competent cells	Agilent	Cat# 260275

(*Continued on next page*)

Continued		
REAGENT or RESOURCE	SOURCE	IDENTIFIER
Chemicals, Peptides, and Recombinant Proteins		
Mitomycin C	Sigma-Aldrich	Cat# M4287
Hydroxyurea	Sigma-Aldrich	Cat# H8627
Doxycycline	Cayman Chemical	Cat# 14422
Mirin	Calbiochem	Cat# 475954-10MG
DNA2 inhibitor (C5)	Judith Campbell; Liu et al., 2016	N/A
CldU	Sigma-Aldrich	Cat# C6891
IdU	Sigma-Aldrich	Cat# I7125
EdU	Life Technologies	Cat# 11590926
Oligofectamine	Life Technologies	Cat# 2252011
Lipofectamine2000	Life Technologies	Cat# 11668-019
FugeneHD	Promega	Cat# E2311
Entellan Mounting Media	Millipore	Cat# HX61088761
Vectashield (with DAPI)	Vectorlabs	Cat# H-1200
Propidium Iodide	Sigma-Aldrich	Cat# P4864
Protein A Sepharose	GE Healthcare	Cat# 17-0780-01
Streptavidin Agarose	Sigma-Aldrich	Cat# S1638
Glutathione Sepharose	GE Healthcare	Cat# 17-0756-01
Diazo-Biotin Azide	Stratech	Cat# CLK-1041-10-JEN
Thymidine	Sigma-Aldrich	Cat# T1895
Fluoroshield	Sigma-Aldrich	Cat# F6182
Histone H3 Peptide - biotinylated	Active Motif	Cat# 81038
Histone H3K4me1 Peptide - biotinylated	Active Motif	Cat# 81040
Histone H3K4me2 Peptide - biotinylated	Active Motif	Cat# 81041
Histone H3K4me3 Peptide - biotinylated	Active Motif	Cat# 81042
FANCD2 (human)	Hiroshi Kimura; Sato et al., 2012	N/A
Histone H3.1 - biotinylated	Active Motif	Cat# 31296
Histone H3K4me1 (EPL) - biotinylated	Active Motif	Cat# 31284
Critical Commercial Assays		
Duolink *In Situ* Red Starter Kit Mouse/Rabbit	Sigma-Aldrich	Cat# DUO92101
SNAP-Cell Starter Kit	NEB	Cat# E9100S
Deposited Data		
Pre-deposited iPOND mass spectrometry data	Dungrawala et al., 2015	N/A
Experimental Models: Cell Lines		
HeLa-H3.1-GFP (WT and K4A)	Hiroshi Kimura; Sato et al., 2012	N/A
HeLa	ATCC	Cat# CCL-2
U-2-OS	ATCC	Cat# HTB-96
U-2-OS Flp-In TRex	Stephen Taylor (University of Manchester)	N/A
A549	ATCC	Cat# CCL-185
DT40s (WT and FANCD2 variants)	Minoru Takata; Sato et al., 2012	N/A
Oligonucleotides		
SETD1A siRNA (3′ UTR)	QIAGEN	Cat# SI05029045
SETD1A siRNA (SmartPool; SP)	Dharmacon	Cat# L-022793-01-0010
BOD1L siRNA (SP)	Dharmacon	Cat# L-017033-02-0005
FANCD2 siRNA (SP)	Dharmacon	Cat# J-016376-05-0005
FBH1 siRNA (SP)	Dharmacon	Cat# J-017404-05-0005
BLM siRNA (SP)	Dharmacon	Cat# J-007287-06-0005

(*Continued on next page*)

Continued		
REAGENT or RESOURCE	SOURCE	IDENTIFIER
BOD1 siRNA (SP)	Dharmacon	Cat# L-015526-02-0005
SETD1B siRNA (SP)	Dharmacon	Cat# J-027025-09-0005
CHD1 siRNA (SP)	Dharmacon	Cat# J-008529-05-0005
CHD4 siRNA (SP)	Dharmacon	Cat# L-009774-00-0005
KMT2B (MLL2) siRNA (SP)	Dharmacon	Cat# L-009670-00-0005
KMT2C (MLL3) siRNA (SP)	Dharmacon	Cat# L-007039-00-0005
BRCA2 siRNA (SP)	Dharmacon	Cat# L-003462-00-0005
MRE11 siRNA (SP)	Dharmacon	Cat# L-009271-00-0005
Cyclin K siRNA (SP)	Dharmacon	Cat# L-029590-00-0005
Control siRNA (luciferase) (CGUACGCGG AAUACUUCGdTdT)	Dharmacon	Cat# CTM-334043
Control siRNA (Negative Control Hi GC)	Life Technologies	Cat# 10317903
BOD1L F1 Fwd (TATCCTGTCGACATGG CCACCAACCCACAGCCGCAG)	Sigma-Aldrich	N/A
BOD1L F1 Rev (CGAGTTAGCGGCCGG GGTTTCTTTGGAATCTTCTTCATA)	Sigma-Aldrich	N/A
BOD1L F2 Fwd (CACGACGTCGACGAAA AAGAAGAGAGGCTTTTAAGA)	Sigma-Aldrich	N/A
BOD1L F2 Rev (CGTTTGAGGGGCCGCT TTCTCCTTTGCTAATGGTAACTT)	Sigma-Aldrich	N/A
Recombinant DNA		
pGEX-3x-BOD1L F1 (aa 1-600 of BOD1L)	This paper	N/A
pGEX-3x-BOD1L F2 (aa 500-1000 of BOD1L)	This paper	N/A
pSNAPm-H3.1	Geneviève Almouzni; Adam et al., 2013	N/A
Software and Algorithms		
ImageJ	NIH	RRID: SCR_003070
David Bioinformatics Resources 6.8	NIAID/NIH	https://david.ncifcrf.gov/; RRID: SCR_001881
Nikon Elements (v.4.5)	Nikon	RRID: SCR_014329

CONTACT FOR REAGENT AND RESOURCE SHARING

Further information and requests for resources and reagents should be directed to, and will be fulfilled by, the Lead Contact, Professor Grant Stewart (g.s.stewart@bham.ac.uk).

EXPERIMENTAL MODEL AND SUBJECT DETAILS

Cell Culture and Generation of Cell Lines

U-2-OS cells (ATCC) were cultured in McCoys 5A medium, supplemented with 10% FBS and penicillin/streptomycin. HeLa cells (ATCC) were grown in Dulbecco's modified Eagle's medium supplemented with 10% fetal calf serum (FCS) (Life Technologies) and penicillin/streptomycin.

U-2-OS-FLAG-SETD1A FL, ΔSET and ΔRRM inducible cell lines were generated by Flp recombinase–mediated integration using U-2-OS-Flp-In T-REx host cells transfected with pcDNA5/FRT-FLAG-SETD1A FL, ΔSET or ΔRRM, together with pOG44. Transfected cells were selected and expanded for testing, cultured as above in the presence of 10% Tet-free FBS (Lonza), and SETD1A variant expression was induced with doxycycline (Sigma-Aldrich).

The generation of HeLa cells expressing both H3.1-GFP WT and mCherry-PCNA was described previously (Sato et al., 2012). The HeLa cells expressing either H3.1-GFP K4A or WT H3.1-GFP were generated using PiggyBac Transposon Vector System (System Biosciences). After cloning the H3.1-GFP K4A or WT H3.1-GFP sequence into PB533A-2 vector, the resulting plasmid was cotransfected into HeLa cells with PiggyBac Transposase vector (PB210PA-1) using FuGene HD (Promega). The cells were then cultured in 1 mg/ml G418 (Nacalai Tesque) to select clones stably expressing either H3.1-GFP K4A or WT H3.1-GFP. The creation of stable

U-2-OS-GFP-RAD51 cells was described in Sato et al. (2016). HeLa and U-2-OS cells used for FRAP analysis were cultured in Dulbecco's modified Eagle's medium (High Glucose; Nacalai Tesque), supplemented with 10% fetal bovine serum (JRH Bioscience) and penicillin/streptomycin.

Wild-type and FANCD2 mutant DT40 cell lines are described in Sato et al. (2012) and were cultured in RPMI medium supplemented with 10% FCS, 1% chicken serum (Sigma Aldrich) and 10 μM β-mercaptoethanol (Life Technologies).

METHOD DETAILS

siRNA Transfections

siRNAs were purchased from Dharmacon as SMARTpool, and all siRNA transfections were performed with 100 nM of siRNA duplexes using Oligofectamine (Life Technologies) or Lipofectamine2000 (Life Technologies). Whenever siRNAs were combined, the total concentration was kept at 100 nM. An siRNA targeting lacZ (CGUACGCGGAAUACUUCGdTdT) or Negative Control Hi GC (Life Technologies; for GFP-RAD51 analysis) was used as "Control siRNA." SETD1A 3′ UTR siRNA was from QIAGEN. See Key Resources Table for siRNA sequences.

Drugs and Inhibitors

HU and MMC were from Sigma Aldrich, and were used as indicated in Figure Legends. dNTP analogs EdU, CldU and IdU were from Sigma Aldrich, and were used as denoted. Thymidine was from Sigma Aldrich. Mirin (100 μM) was from Calbiochem, and C5 (DNA2 inhibitor) (20 μM) was obtained from Judith Campbell (Liu et al., 2016).

Cloning

Constructs encoding pcDNA5/FRT-FLAG-SETD1A FL, ΔSET and ΔRRM variants were obtained from David Skalnik (Lee and Skalnik, 2005). pSNAPm-H3.1 was obtained from Geneviève Almouzni (Adam et al., 2013). Plasmids encoding WT or chaperone-dead (R302W) mCherry-FANCD2 are described in Sato et al. (2012). GST-tagged BOD1L fragments were amplified by PCR from human cDNA and cloned into the SalI-*NotI* restriction sites of pGEX-3X. Fragments 1 and 2 correspond to amino acids 1-600 and 500-1000 of BOD1L respectively. See Key Resources Table for primer sequences.

Colony Survival Assays

For colony survival assays, siRNA-transfected HeLa or HeLa-H3.1-GFP cells were plated at low density, and exposed to increasing doses of MMC. Colonies were fixed and stained after 14 days with 2% methylene blue (Sigma Aldrich) in 50% ethanol. Data are expressed as a percentage survival normalized to non-treatment control for each siRNA.

DNA Fibers

DNA fibers were carried out as described previously (Higgs et al., 2015). For quantification of replication structures, cells were treated with MMC (50 ng/ml) for 24 hr prior to analog labeling, and at least 250 structures were counted per experiment. For fork resection experiments, cells were pulse-labeled with CldU and IdU for 20 min each before a 5 hr exposure to 4 mM HU, and at least 200 replication forks were analyzed per experimental condition. For fork resection experiments in DT40 cells, cells were pulse-labeled as above, spun down in media containing 4 mM HU and then exposed to HU for 5 hr as above. The lengths of red or green labeled tracts were measured using ImageJ (National Institutes of Health; https://imagej.nih.gov/ij/) and arbitrary length values were converted into micrometers using the scale bars created by the microscope. Mean tract ratios, SEM values and statistical analyses for all DNA fiber analysis can be found in Table S1.

iPOND

iPOND was performed on HEK293T cells as described (Dungrawala et al., 2015). Briefly, light and heavy labeled cells were pulse-labeled with EdU, incubated with 3 mM HU for 4 hr where indicated, mixed prior to the click reaction, and DNA-protein complexes were captured with streptavidin-coupled beads. Samples were separated by SDS-PAGE, excised, reduced with DTT and carbamidomethylated, and then destained. Polypeptides were digested with trypsin and analyzed by mass spectrometry using a Q Exactive mass spectrometer in conjunction with MaxQuant. Plots are amalgamated from published log2 abundance data for the conditions denoted.

Proximity Ligation Assay (PLA)

EdU-PLA to detect proteins at nascent DNA was performed as described (Taglialatela et al., 2017), with minor modifications. Briefly, cells were pulsed with 10 μM EdU for 10 min cells before being permeabilised with nuclear extraction buffer (10 mM PIPES, 20 mM NaCl, 3 mM $MgCl_2$, 300 mM sucrose, 0.5% Triton X-100). Where appropriate, cells were exposed to 4 mM HU for 5 hr before pre-extraction. Cells were then fixed with 3.6% paraformaldehyde for 10 min at room temperature and blocked with ADB (Antibody Dilution Buffer; 3% BSA in PBS) overnight at 4°C. EdU was conjugated to biotin by incubating cells in Click reaction buffer for 1 hr at room temperature containing 10 μM Diazo-biotin Azide, 10 mM sodium ascorbate, and 1 mM copper (II) sulfate in PBS. Following the Click reaction, cells were blocked in ABD before incubated in primary antibodies before proceeding with proximity ligation using a

Duolink Detection Kit in combination with anti-Mouse PLUS and anti-Rabbit MINUS PLA Probes (Sigma Aldrich) according to the manufacturer's instructions. Cells were imaged as below and the number of PLA signals per nuclei quantified.

Flow Cytometry

HeLa cells were harvested, fixed in 70% ethanol at −20°C for 1 hr, and permeabilised with 0.25% Triton X-100 for 15 min at 4°C. Cells were then washed twice with 1% BSA in PBS, and stained with 25 μg/ml propidium iodide containing 0.1 mg/ml RNase A. Cells were analyzed using an Accuri flow cytometer (BDBiosciences) in conjunction with CFlowplus software. Data represent that obtained from at least 30,000 cells.

Metaphase Spreads

Chromosomal aberrations and radial chromosomes were scored in Giemsa stained metaphase spreads. For chromosome aberrations, demecolcine (Sigma) was added 3-4 hr prior to harvesting at a final concentration of 0.2 μg/ml. Cells were harvested by trypsinization, subjected to hypotonic shock for 1 hr at 37°C in 0.3 M sodium citrate and fixed in 3:1 methanol:acetic acid solution. Cells were dropped onto acetic acid humidified slides, stained for 15 min in Giemsa-modified (Sigma) solution (5% v/v in H2O) and washed in water for 5 min.

Immunofluorescence, Microscopy, and Image Analysis

HeLa, U-2-OS, HeLa-H3.1-GFP and U-2-OS-FLAG-SETD1A cells were grown on glass coverslips. Cells were washed with PBS twice before fixation. In all cases, cells were permeabilised with nuclear extraction buffer for 5 min on ice prior to fixation in 3.6% paraformaldehyde for 10 min at room temperature. After fixation, cells were washed with PBS three times and then blocked with ADB for 1 hr at 4°C. Cells were incubated with primary antibody (diluted in ADB) for 1 hr at room temperature, washed with PBS and then counterstained with Alexa Fluor-conjugated secondary antibodies (diluted in ADB) for 1 hr at room temperature. Cells were then washed twice with ADB and coverslips were mounted onto glass slides with Vectashield mounting agent containing 0.4 μg/ml DAPI (Vectashield). Images were taken using a Nikon Eclipse Ni microscope equipped with a 60X oil lens, and were acquired and analyzed using Elements v4.5 software (Nikon). For RPA intensity analyses, the intensity of nuclear foci was quantified for each cell using ImageJ. See Key Resources Table for details of antibodies used.

SNAP Labeling of Histones

U-2-OS cells were grown on glass coverslips, transfected with siRNA, incubated for 48 hr, and transfected with pSNAPm-H3.1 using FuGene HD. 24 hr post DNA transfection, pre-existing SNAP-H3.1 was labeled with SNAP-cell 505 star (New England Biolabs) according to the manufacturer's instructions ('pulse'). Alternatively, pre-existing H3.1 was quenched by incubating cells with SNAP-cell Block (New England Biolabs), and then cells were pulsed as described above ('quench-pulse'), or released into 2 mM HU for 2 hr before newly synthesized SNAP-H3.1 was labeled as above ('quench-HU-pulse'). In all cases, cells were fixed with ice cold methanol for 10 min before being mounted onto glass slides. Images were taken as above, and the intensity of nuclear SNAP signal was quantified for each cell using ImageJ.

Fluorescence Recovery after Photobleaching (FRAP)

FRAP was performed as previously described (Sato et al., 2012, 2016). HeLa cells expressing both H3.1-GFP and mCherry-PCNA were grown on a glass-bottom dish (Mat-tek), transfected with siRNA, incubated for 48 h, and treated with MMC (50 ng/ml) for 12-24 hr. FRAP was then performed using an FV-1000 with a PlanApoN 60x OSC (NA = 1.4) oil-immersion objective lens (Olympus) at 37°C under 5% CO2. Three confocal images were collected (800x800 pixels, zoom 2, scan speed 2 μs/pixel, Kalman filtration for four scans, pinhole 800 μm, 0.1% 488-nm laser transmission, and 20% 543-nm laser transmission). Afterward, one half of PCNA-foci positive nucleus was bleached using 75% transmission of 488 nm and 100% of 515 nm (three iterations), and images were obtained using the original setting at 5 min intervals. The fluorescence intensities of the unbleached and bleached areas and background were measured using ImageJ 1.46r software (https://imagej.nih.gov/ij/). After background subtraction, the relative intensity of the bleached area to the unbleached area in each time point was calculated and normalized to the average intensity before bleaching. H3.1-GFP K4A was analyzed by the same procedure with or without mitomycin C (50 ng/ml).

For GFP-RAD51 FRAP, U-2-OS cells expressing GFP-RAD51 were grown on a glass-bottom dish, transfected with siRNA, incubated for 24 hr, and transfected with either the RNAi-resistant mCherry-FANCD2 or mCherry-FANCD2 (R302W) vector using FuGene HD. The next day, cells were then treated with doxycycline (1-10 ng/ml) and MMC (100 ng/ml) for 12-24 hr, as described previously (Sato et al., 2016). FRAP was then performed using an FV-1000 confocal microscopy with a PlanApoN 60x OSC (NA = 1.4) oil-immersion objective lens (Olympus) at 37°C under 5% CO2. Six z-slice images (0.5 μm intervals) containing GFP-RAD51 foci were collected (256 × 32 pixels, scan speed 2 μs/pixel, zoom 12, pinhole 800 μm, 0.1% 488-nm laser transmission, and 20% 543-nm laser transmission). Afterward, a single GFP-RAD51 focus was photobleached using 100% transmission of 488-nm laser (1 iteration for each z-plane), and images were collected using the original settings at 1.89 s intervals for 100 s. The 2D maximum z-projection was reconstructed, and the fluorescence intensities of the bleached area were measured using ImageJ 1.46r software. After the background subtraction, the intensity was normalized to the initial intensity before bleaching.

Western Blotting, Pull-downs, and Immunoprecipitations

For western blotting, whole cell extracts were obtained by lysis in UTB buffer (8 M Urea, 50 mM Tris, 150 mM β-mercaptoethanol, protease inhibitor cocktail (Roche)). Cell extracts were sonicated, clarified by centrifugation, and protein concentration was determined by Bradford Assay (Bio-Rad). Polypeptides were separated by SDS-PAGE, transferred onto nitrocellulose membrane, incubated with primary antibody overnight, followed by HRP-linked secondary antibody for 1hr at room temperature. The signal was detected using ECL western blotting substrate (GE Healthcare).

For immunoprecipitations, HeLa nuclear cells extracts (Cilbiotech) were clarified by centrifugation at 44,000 x g, immunoprecipitated with 5 μg of the indicated antibody or IgG for 3 hr at 4°C. After further clarification, immune complexes were isolated using protein-A Sepharose (GE Healthcare), and analyzed by immunoblotting as above.

For GST pull-downs, 1 μg of affinity-purified GST or GST fusion protein was incubated with pre-clarified HeLa nuclear cells extract for 3 hr at 4°C. Fusion proteins and binding partners were isolated using glutathione Sepharose (GE Healthcare) and analyzed as above. See Key Resources Table for details of antibodies used.

For histone peptide pull-downs, lyophilized biotinylated peptides (Active Motif) were resuspended and immobilised onto Strepavidin Agarose beads (Sigma Aldrich) as described in Wysocka (2006) at a final concentration of 250 ng/ul. Immobilised peptides were then incubated with clarified HeLa nuclear cells extracts for 3 hr at 4°C before being washed with buffer D (100 mM KCl, 20 mM HEPES pH 7.9, 20% v/v glycerol, 0.2 mM EDTA, 0.2% Triton X-100, protease inhibitors). Alternatively, immobilised peptides were incubated with 100 ng of purified human FANCD2 (Sato et al., 2012; Takahashi et al., 2014) in binding buffer (20 mM Tris pH 7.5, 150 mM NaCl, 300 mM sucrose, 1 mM $MgCl_2$, protease inhibitors) for 3 hr at 4°C. Bound protein was washed with buffer D. In both cases, peptide binding partners were analyzed by immunoblotting as above.

For histone pull-downs, lyophilised recombinant biotinylated H3 or H3K4me1 (EPL) (Active Motif) were resuspended in 25 mM Tris pH 7.5, 150 mM NaCl, 5% glycerol at a final concentration of 1 μg/ul, and then incubated with 100 ng of purified human FANCD2 in binding buffer for 3 hr at 4°C. Histones were isolated with Strepavidin Agarose beads for a further 3 hr at 4°C, washed as above and analyzed by immunoblotting.

QUANTIFICATION AND STATISTICAL ANALYSIS

Differences in survival assays were analyzed by two-way ANOVA. Statistical differences in all other cases were determined by Student's t test, except for fork asymmetry, fork resection and EdU-PLA data, which were analyzed by Mann-Whitney rank sum test. Statistical differences denoted in the Figures were determined by comparison to the relevant control-treated samples unless otherwise indicated. In all cases: * = $p < 0.05$; ** = $p < 0.01$; *** = $p < 0.001$.

Molecular Cell, Volume *71*

Supplemental Information

Histone Methylation by SETD1A Protects Nascent DNA through the Nucleosome Chaperone Activity of FANCD2

Martin R. Higgs, Koichi Sato, John J. Reynolds, Shabana Begum, Rachel Bayley, Amalia Goula, Audrey Vernet, Karissa L. Paquin, David G. Skalnik, Wataru Kobayashi, Minoru Takata, Niall G. Howlett, Hitoshi Kurumizaka, Hiroshi Kimura, and Grant S. Stewart

Figure S1

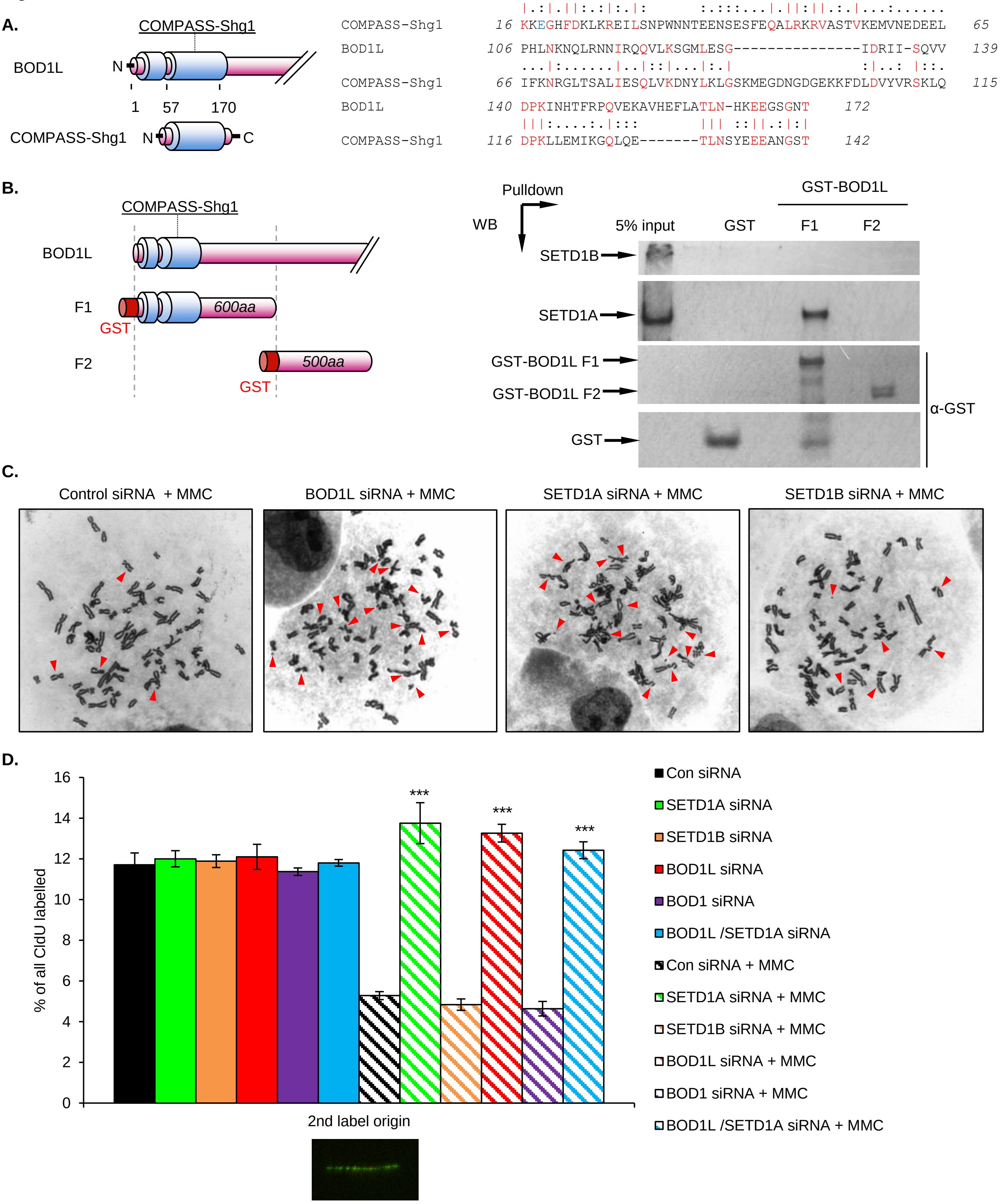
A.
COMPASS-Shg1
BOD1L
N
1
57
170
COMPASS-Shg1
N
C
BOD1L 62 KSQGLFDQFRRDCLA------DVDTKPAYQNLRQRVDNFVANHLATHTWS 105
COMPASS-Shg1 16 KKEGHFDKLKREILSNPWNNTEENSESFEQALRKRVASTVKEMVNEDEEL 65
BOD1L 106 PHLNKNQLRNNIRQQVLKSGMLESG---------------IDRII-SQVV 139
COMPASS-Shg1 66 IFKNRGLTSALIESQLVKDNYLKLGSKMEGDNGDGEKKFDLDVYVRSKLQ 115
BOD1L 140 DPKINHTFRPQVEKAVHEFLATLN-HKEEGSGNT 172
COMPASS-Shg1 116 DPKLLEMIKGQLQE-------TLNSYEEEANGST 142
B.
COMPASS-Shg1
BOD1L
F1
600aa
GST
F2
500aa
GST
Pulldown
GST-BOD1L
WB
5% input
GST
F1
F2
SETD1B
SETD1A
GST-BOD1L F1
GST-BOD1L F2
GST
α-GST
C.
Control siRNA + MMC
BOD1L siRNA + MMC
SETD1A siRNA + MMC
SETD1B siRNA + MMC
D.
% of all CldU labelled
0
2
4
6
8
10
12
14
16
***
***
***
2nd label origin
Con siRNA
SETD1A siRNA
SETD1B siRNA
BOD1L siRNA
BOD1 siRNA
BOD1L /SETD1A siRNA
Con siRNA + MMC
SETD1A siRNA + MMC
SETD1B siRNA + MMC
BOD1L siRNA + MMC
BOD1 siRNA + MMC
BOD1L /SETD1A siRNA + MMC

Figure S2

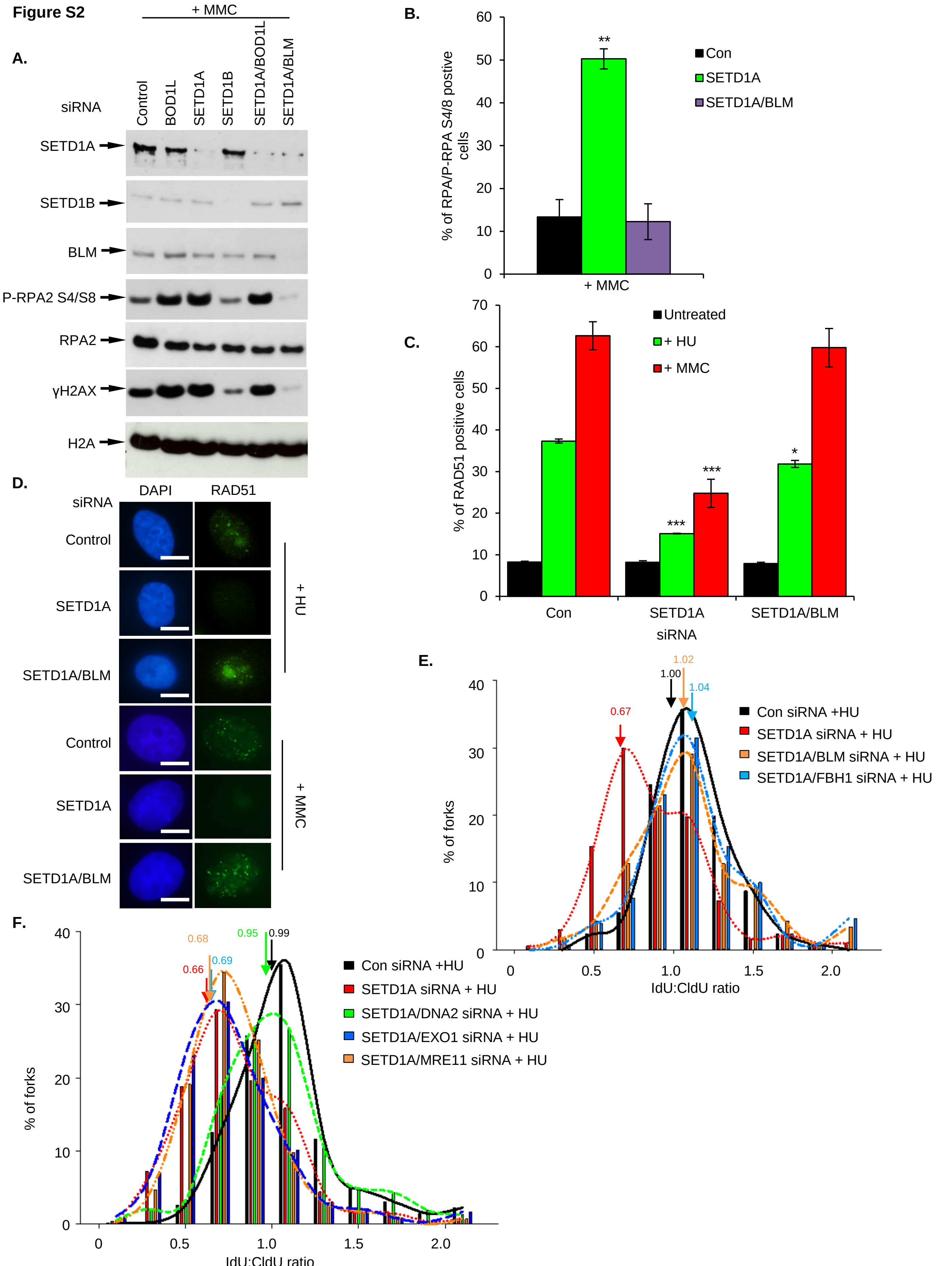

A.
+ MMC
siRNA
Control
BOD1L
SETD1A
SETD1B
SETD1A/BOD1L
SETD1A/BLM
SETD1A
SETD1B
BLM
P-RPA2 S4/S8
RPA2
γH2AX
H2A
B.
% of RPA/P-RPA S4/8 postive cells
60
50
40
30
20
10
0
**
Con
SETD1A
SETD1A/BLM
+ MMC
C.
% of RAD51 positive cells
70
60
50
40
30
20
10
0
Untreated
+ HU
+ MMC
***
***
*
Con
SETD1A
SETD1A/BLM
siRNA
D.
siRNA
DAPI
RAD51
Control
SETD1A
SETD1A/BLM
+ HU
Control
SETD1A
SETD1A/BLM
+ MMC
E.
% of forks
40
30
20
10
0
0.67
1.00
1.02
1.04
Con siRNA +HU
SETD1A siRNA + HU
SETD1A/BLM siRNA + HU
SETD1A/FBH1 siRNA + HU
0
0.5
1.0
1.5
2.0
IdU:CldU ratio
F.
% of forks
40
30
20
10
0
0.68
0.66
0.69
0.95
0.99
Con siRNA +HU
SETD1A siRNA + HU
SETD1A/DNA2 siRNA + HU
SETD1A/EXO1 siRNA + HU
SETD1A/MRE11 siRNA + HU
0
0.5
1.0
1.5
2.0
IdU:CldU ratio

Figure S3

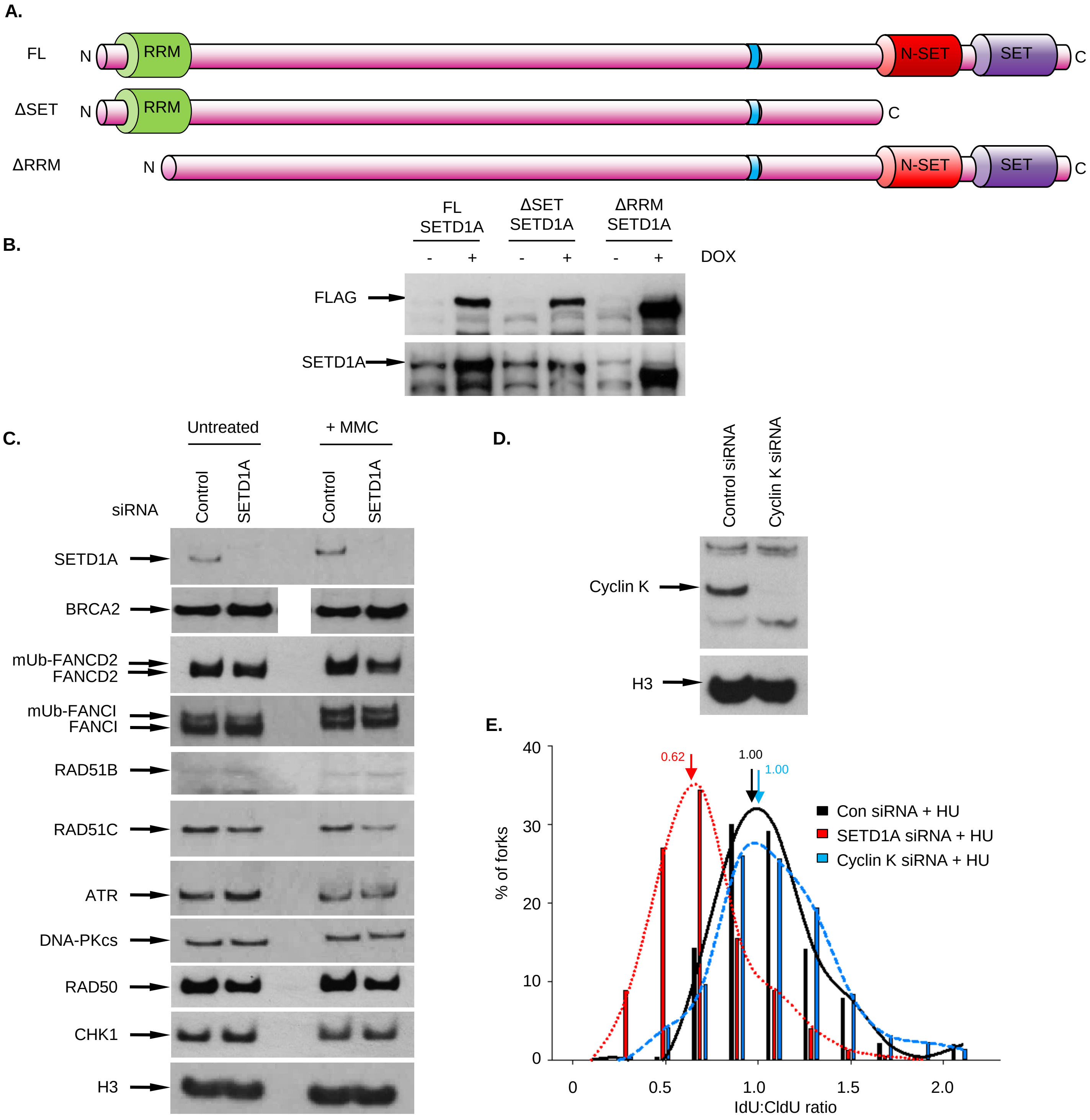
A.
FL
N
RRM
N-SET
SET
C
ΔSET
ΔRRM
B.
FL SETD1A
ΔSET SETD1A
ΔRRM SETD1A
- + - + - +
DOX
FLAG
SETD1A
C.
Untreated
+ MMC
siRNA
Control
SETD1A
SETD1A
BRCA2
mUb-FANCD2
FANCD2
mUb-FANCI
FANCI
RAD51B
RAD51C
ATR
DNA-PKcs
RAD50
CHK1
H3
D.
Control siRNA
Cyclin K siRNA
Cyclin K
H3
E.
0.62
1.00
1.00
Con siRNA + HU
SETD1A siRNA + HU
Cyclin K siRNA + HU
% of forks
0
10
20
30
40
0
0.5
1.0
1.5
2.0
IdU:CldU ratio

Figure S4

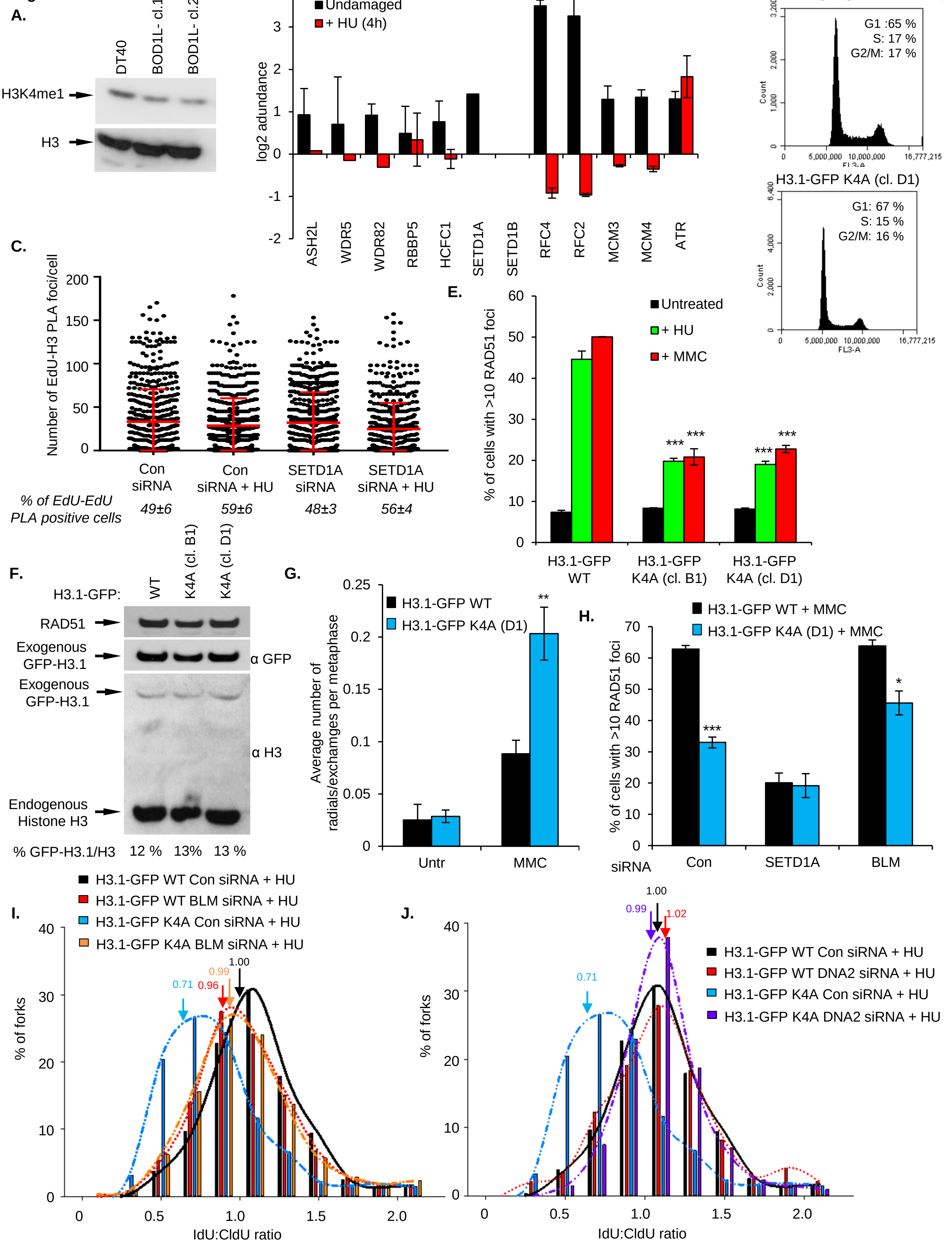

A.
DT40
BOD1L- cl.1
BOD1L- cl.2
H3K4me1
H3
B.
Undamaged
+ HU (4h)
log2 adundance
ASH2L
WDR5
WDR82
RBBP5
HCFC1
SETD1A
SETD1B
RFC4
RFC2
MCM3
MCM4
ATR
D.
H3.1-GFP WT
G1 :65 %
S: 17 %
G2/M: 17 %
H3.1-GFP K4A (cl. D1)
G1: 67 %
S: 15 %
G2/M: 16 %
Count
FL3-A
C.
Number of EdU-H3 PLA foci/cell
Con siRNA
Con siRNA + HU
SETD1A siRNA
SETD1A siRNA + HU
% of EdU-EdU PLA positive cells
49±6
59±6
48±3
56±4
E.
Untreated
+ HU
+ MMC
% of cells with >10 RAD51 foci
H3.1-GFP WT
H3.1-GFP K4A (cl. B1)
H3.1-GFP K4A (cl. D1)
F.
H3.1-GFP:
WT
K4A (cl. B1)
K4A (cl. D1)
RAD51
Exogenous GFP-H3.1
α GFP
Exogenous GFP-H3.1
α H3
Endogenous Histone H3
% GFP-H3.1/H3
12 %
13%
13 %
G.
H3.1-GFP WT
H3.1-GFP K4A (D1)
Average number of radials/exchamges per metaphase
Untr
MMC
H.
H3.1-GFP WT + MMC
H3.1-GFP K4A (D1) + MMC
% of cells with >10 RAD51 foci
siRNA
Con
SETD1A
BLM
I.
H3.1-GFP WT Con siRNA + HU
H3.1-GFP WT BLM siRNA + HU
H3.1-GFP K4A Con siRNA + HU
H3.1-GFP K4A BLM siRNA + HU
% of forks
IdU:CldU ratio
0.71
0.96
0.99
1.00
J.
H3.1-GFP WT Con siRNA + HU
H3.1-GFP WT DNA2 siRNA + HU
H3.1-GFP K4A Con siRNA + HU
H3.1-GFP K4A DNA2 siRNA + HU
% of forks
IdU:CldU ratio
0.71
0.99
1.00
1.02

Figure S5

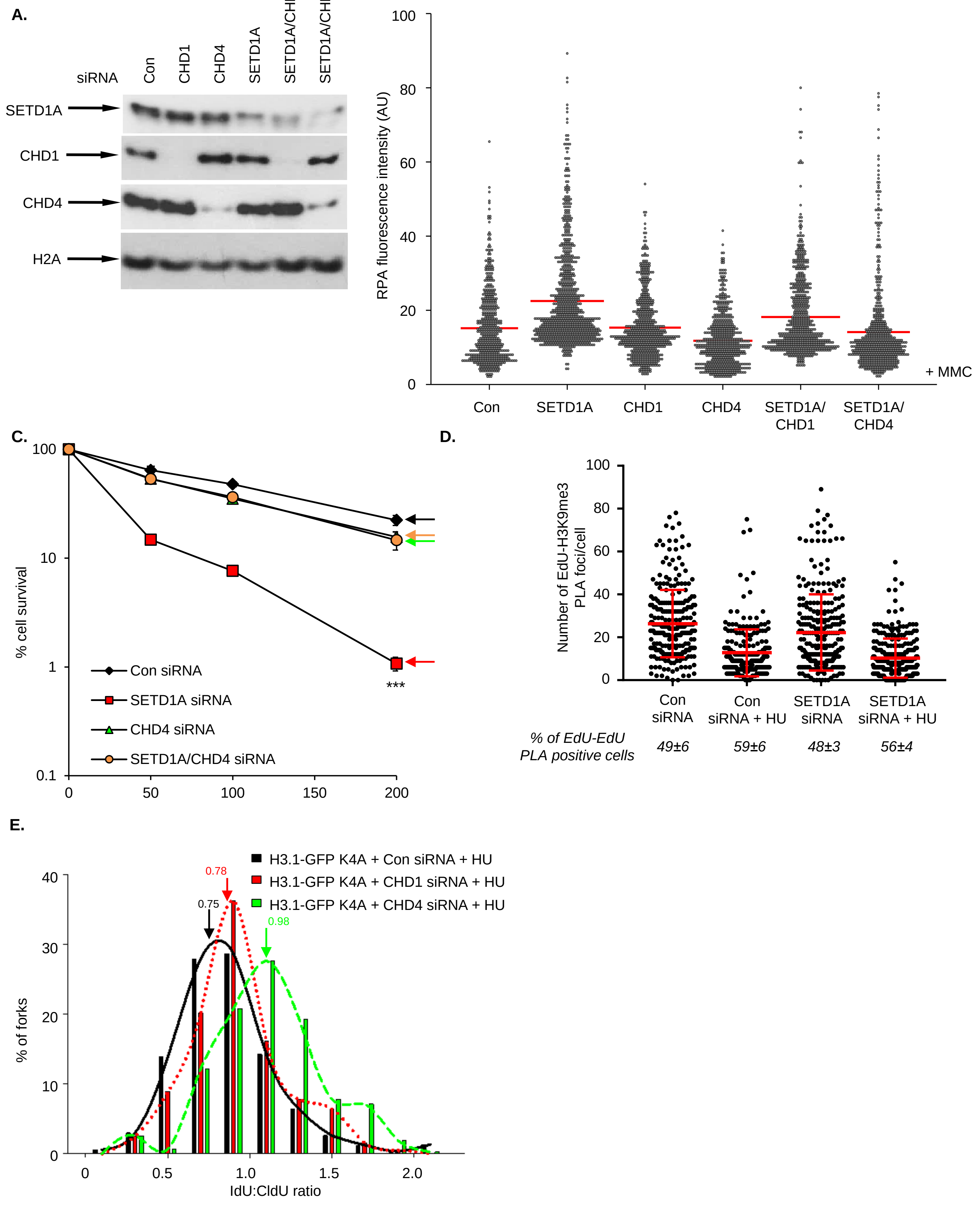

A.
siRNA
Con
CHD1
CHD4
SETD1A
SETD1A/CHD1
SETD1A/CHD4
SETD1A
CHD1
CHD4
H2A
B.
RPA fluorescence intensity (AU)
100
80
60
40
20
0
+ MMC
Con
SETD1A
CHD1
CHD4
SETD1A/ CHD1
SETD1A/ CHD4
C.
% cell survival
100
10
1
0.1
0
50
100
150
200
***
Con siRNA
SETD1A siRNA
CHD4 siRNA
SETD1A/CHD4 siRNA
D.
Number of EdU-H3K9me3 PLA foci/cell
100
80
60
40
20
0
Con siRNA
Con siRNA + HU
SETD1A siRNA
SETD1A siRNA + HU
% of EdU-EdU PLA positive cells
49±6
59±6
48±3
56±4
E.
% of forks
40
30
20
10
0
0
0.5
1.0
1.5
2.0
IdU:CldU ratio
H3.1-GFP K4A + Con siRNA + HU
H3.1-GFP K4A + CHD1 siRNA + HU
H3.1-GFP K4A + CHD4 siRNA + HU
0.78
0.75
0.98

Figure S6

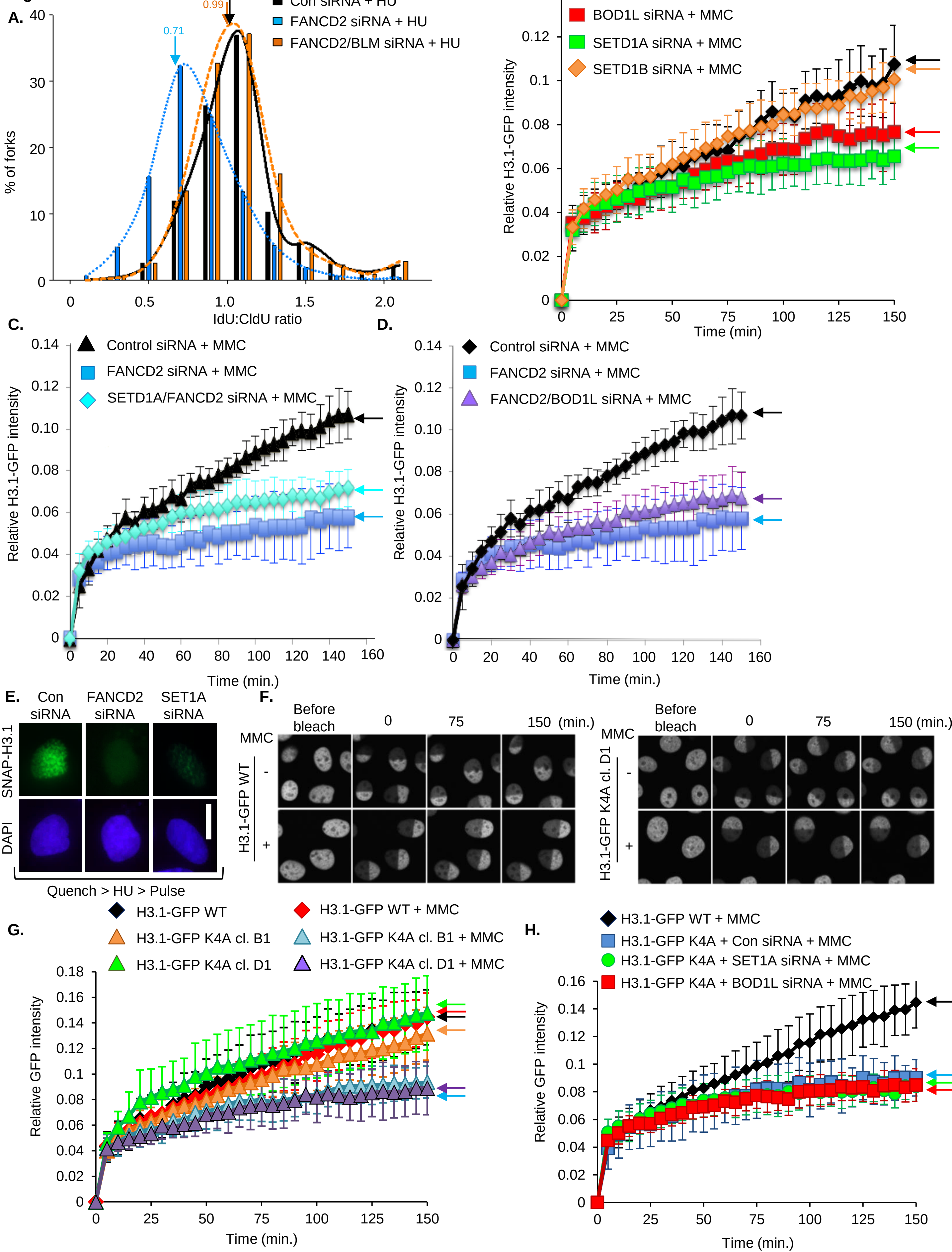
A.
Con siRNA + HU
FANCD2 siRNA + HU
FANCD2/BLM siRNA + HU
0.99
0.99
0.71
% of forks
IdU:CldU ratio
B.
Con siRNA + MMC
BOD1L siRNA + MMC
SETD1A siRNA + MMC
SETD1B siRNA + MMC
Relative H3.1-GFP intensity
Time (min)
C.
Control siRNA + MMC
FANCD2 siRNA + MMC
SETD1A/FANCD2 siRNA + MMC
Relative H3.1-GFP intensity
Time (min.)
D.
Control siRNA + MMC
FANCD2 siRNA + MMC
FANCD2/BOD1L siRNA + MMC
Relative H3.1-GFP intensity
Time (min.)
E.
Con siRNA
FANCD2 siRNA
SET1A siRNA
SNAP-H3.1
DAPI
Quench > HU > Pulse
F.
Before bleach
0
75
150 (min.)
MMC
H3.1-GFP WT
H3.1-GFP K4A cl. D1
G.
H3.1-GFP WT
H3.1-GFP K4A cl. B1
H3.1-GFP K4A cl. D1
H3.1-GFP WT + MMC
H3.1-GFP K4A cl. B1 + MMC
H3.1-GFP K4A cl. D1 + MMC
Relative GFP intensity
Time (min.)
H.
H3.1-GFP WT + MMC
H3.1-GFP K4A + Con siRNA + MMC
H3.1-GFP K4A + SET1A siRNA + MMC
H3.1-GFP K4A + BOD1L siRNA + MMC
Relative GFP intensity
Time (min.)

Figure S7

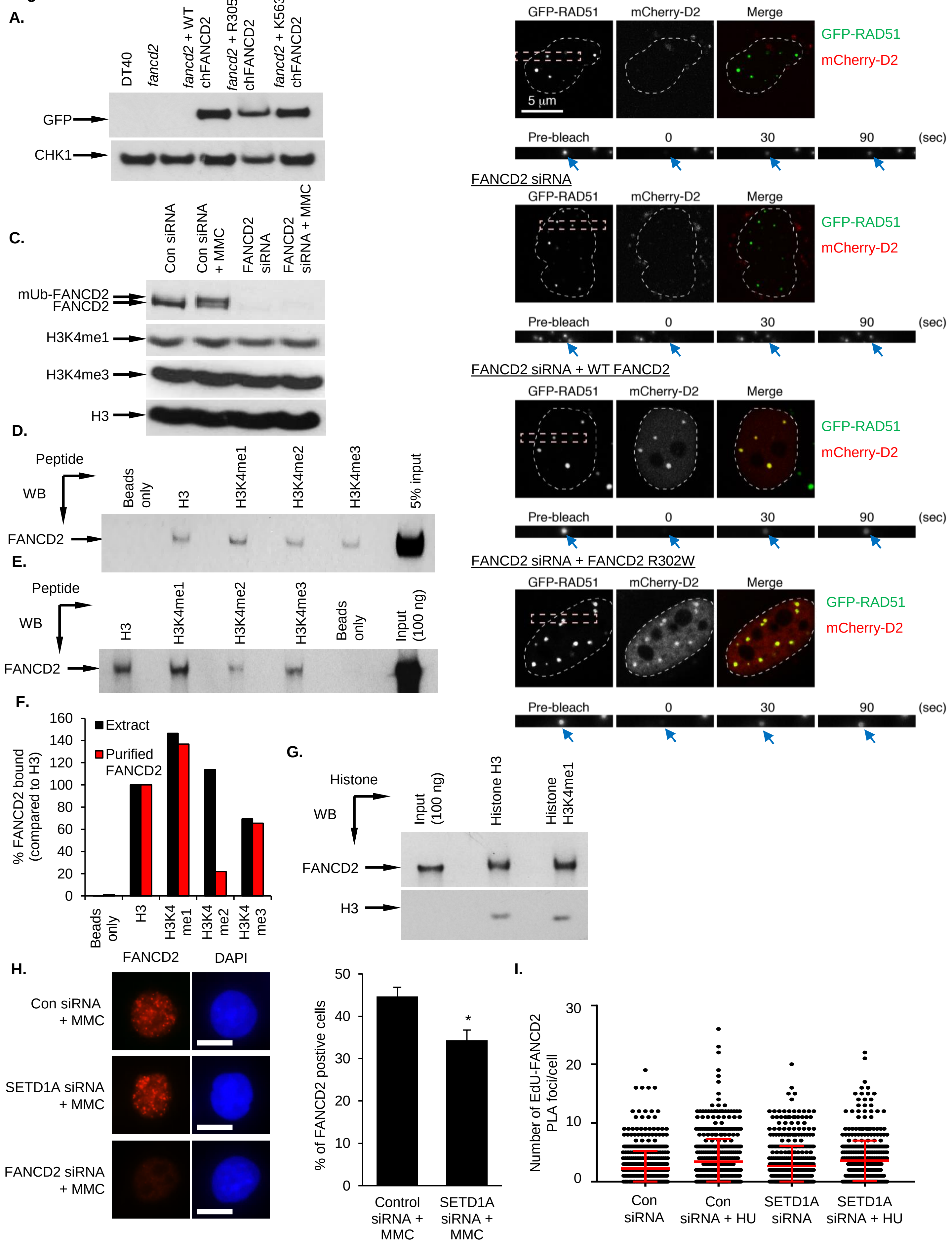
A.
DT40
fancd2
fancd2 + WT chFANCD2
fancd2 + R305W chFANCD2
fancd2 + K563R chFANCD2
GFP
CHK1
B. Control siRNA
GFP-RAD51
mCherry-D2
Merge
5 μm
Pre-bleach
0
30
90
(sec)
FANCD2 siRNA
FANCD2 siRNA + WT FANCD2
FANCD2 siRNA + FANCD2 R302W
C.
Con siRNA
Con siRNA + MMC
FANCD2 siRNA
FANCD2 siRNA + MMC
mUb-FANCD2
FANCD2
H3K4me1
H3K4me3
H3
D.
Peptide
WB
Beads only
H3
H3K4me1
H3K4me2
H3K4me3
5% input
FANCD2
E.
Input (100 ng)
F.
Extract
Purified FANCD2
% FANCD2 bound (compared to H3)
160
140
120
100
80
60
40
20
0
Beads only
H3
H3K4 me1
H3K4 me2
H3K4 me3
G.
Histone
Input (100 ng)
Histone H3
Histone H3K4me1
H.
FANCD2
DAPI
Con siRNA + MMC
SETD1A siRNA + MMC
FANCD2 siRNA + MMC
% of FANCD2 postive cells
50
40
30
20
10
0
*
Control siRNA + MMC
SETD1A siRNA + MMC
I.
Number of EdU-FANCD2 PLA foci/cell
30
20
10
0
Con siRNA
Con siRNA + HU
SETD1A siRNA
SETD1A siRNA + HU

HISTONE METHYLATION BY SETD1A PROTECTS NASCENT DNA THROUGH THE NUCLEOSOME CHAPERONE ACTIVITY OF FANCD2

SUPPLEMENTAL INFORMATION

SUPPLEMENTAL FIGURE LEGENDS

Figure S1, related to Figure 1: BOD1L and SETD1A functionally interact to regulate the replication stress response. (A) *Left:* Schematic comparison of the N-terminus of human BOD1L and the yeast COMPASS-Shg1 protein. *Right:* Amino acid sequence alignment of BOD1L and COMPASS-Shg1. Conserved residues are denoted (red). **(B)** Analysis of proteins that co-precipitate with GST or GST–BOD1L fragments isolated by glutathione-sepharose from HeLa nuclear cell extracts. Inputs and co-precipitates were analyzed by immunoblotting with the indicated antibodies. **(C)** HeLa cells were treated as in Figure 1C. Representative metaphase spreads are shown, with chromosomal damage denoted by arrowheads. **(D)** HeLa cells were transfected with the indicated siRNAs for 72 h, exposed to 50 ng/ml MMC for 24 h, then pulsed for 20 min with sequential pulses of CldU and IdU. DNA was spread onto glass slides and visualised with antibodies to CldU and IdU. DNA fibres were quantified, and the percentage of new origins is displayed.

Figure S2, related to Figure 2: SETD1A protects RAD51 from BLM/FBH1 to prevent over-resection of stalled replication forks. (A) HeLa cells were transfected with the indicated siRNAs for 72 h, exposed to 50 ng/ml MMC for 24 h, and WCE were analysed by immunoblotting. **(B-D)** Transfected HeLa cells from (A) were left untreated or treated with MMC or HU (4 mM for 5 h) as indicated, immunostained with antibodies to antibodies to RPA2 and phospho-RPA S4/S8 (B) or RAD51 (C), and foci formation was analysed by fluorescence microscopy. Plots indicate quantification of foci-positive cells from three independent experiments. Representative images of RAD51 foci are shown in (D). **(E-F)** U-2-OS cells were transfected with the indicated siRNAs for 72 h, pulsed for 20 min each with CldU and IdU, and exposed to 4 mM HU for 5 h. DNA was visualised with antibodies to CldU and IdU, and tract length was calculated. Plots denote the

average ratios of IdU:CldU label length from three independent experiments. Arrows indicate mean ratios. Average values and SEM are denoted in Table S1.

Figure S3, related to Figure 4: The methyltransferase activity of SETD1A is required for fork protection after replication stress. (A) Schematic of the FL, ΔSET and ΔRRM SETD1A constructs. **(B)** U-2-OS Flp-In T-Rex cell lines bearing the indicated SETD1A constructs were exposed to doxycycline for 24 h, and WCE prepared. Extracts were analysed by immunoblotting with the indicated antibodies. **(C)** U-2-OS cells were transfected with the indicated siRNAs for 72 h, exposed to 50 ng/ml MMC for 24 h, and WCE were analysed by immunoblotting. Spaces denote removal of irrelevant lanes. **(D-E)** U-2-OS cells were transfected with the indicated siRNAs for 72 h, and cells were either harvested for immunoblotting (D) or were pulsed for 20 min each with CldU and IdU, and exposed to 4 mM HU for 5 h (E). DNA was visualised with antibodies to CldU and IdU, and tract length was calculated. Plots denote the average ratios of IdU:CldU label length from three independent experiments. Arrows indicate mean ratios. Average values and SEM are denoted in Table S1.

Figure S4, related to Figure 5: SETD1A-mediated H3.1 methylation is required for fork protection after replication stress. (A) WCE of WT DT40s (cl.18), and two clones lacking exons 1-5 and exon 10 of BOD1L (cl.1 and cl.2), were analysed by immunoblotting with the indicated antibodies. **(B)** Data from mass-spectometry analysis of EdU-coprecipitates from HEK293T cells left untreated or pulsed with HU for 4 h. Data is taken from (Dungrawala *et al.,* 2015). **(C)** Quantification of PLA signals between EdU and H3 in U-2-OS cells transfected with the indicated siRNAs. Where denoted, cells were exposed to 4 mM HU for 5 h. The mean±SD number of Biotin/Biotin PLA-signal positive cells (below) was used as control for the number of S-phase cells in each condition. **(D)** HeLa cells expressing WT H3.1-GFP or a K4A mutant (clone D1) were fixed and cell cycle profiles analysed by flow cytometry. Representative profiles with the mean percentages from three independent experiments are shown. **(E)** Plots indicate quantification of cells from Figure 5F, and represent enumeration of cells with >10 foci from three independent experiments. **(F)** WCE of untreated cells from (E) were analysed by immunoblotting with the

indicated antibodies. **(G)** Quantification of the incidence of radial chromosome formation in cells from Figure 5H. **(H)** HeLa cells from (D) were transfected with the indicated siRNAs and treated as above. Cells were immunostained with antibodies to antibodies to RAD51, and cells with >10 foci enumerated. Data represents mean and SEM from three independent experiments. **(I-J)** HeLa cells from (D) were transfected with the indicated siRNAs, pulsed for 20 min each with CldU and IdU, and exposed to 4 mM HU for 5 h (E). DNA was visualised with antibodies to CldU and IdU, and tract length was calculated. Plots denote the average ratios of IdU:CldU label length from three independent experiments. Arrows indicate mean ratios. Average values and SEM are denoted in Table S1.

Figure S5, related to Figure 6: Histone methylation protects against CHD4-mediated fork degradation. (A) WCE of U-2-OS cells from Figure 6A were subjected to immunoblotting using the indicated antibodies. **(B)** U-2-OS cells from Figure 6A were immunostained with antibodies to RPA2, foci formation was analysed by fluorescence microscopy, and fluorescence intensity per nucleus was quantified using ImageJ. Lines denote mean values from three independent experiments. **(C)** HeLa cells were transfected with the indicated siRNAs, exposed to the indicated doses of mitomycin C (MMC), left to form colonies for 14 days, and then stained with methylene blue and colonies counted. Error bars = SEM. **(D)** Quantification of PLA signals between EdU and H3K9me3 in U-2-OS cells transfected with the indicated siRNAs. Where denoted, cells were exposed to 4 mM HU for 5 h. The mean±SD number of Biotin/Biotin PLA-signal positive cells (below) was used as control for the number of S-phase cells in each condition. **(E)** Stable HeLa cells expressing WT H3.1-GFP or K4A (clone D1) were transfected with the indicated siRNAs, pulsed for 20 min each with CldU and IdU, and exposed to 4 mM HU for 5 h. Plots denote the average ratios of IdU:CldU label length from three independent experiments. Arrows indicate mean ratios. Average values, SEM and p values from three independent experiments are denoted in Table S1.

Figure S6, related to Figure 7: SETD1A and FANCD2 promote histone H3 remodelling and fork protection. (A) U-2-OS cells were transfected with the indicated siRNAs, treated as in Figure

S2E, and tract length was calculated. Plots denote the average ratios of IdU:CldU label length, and arrows indicate mean ratios. Average values and SEM from three independent experiments are given in Table S1. **(B-D)** Stable HeLa cells expressing H3.1-GFP WT were transfected with the indicated siRNAs, exposed to 50 ng/ml MMC for 24 h, and the mobility of histone H3.1-GFP was analysed by FRAP. Graphs represent mean relative fluorescence intensities with SD (n = 10-11). In (C) and (D), control siRNA and FANCD2 siRNA data are presented in for comparison. (**E**) Representative images of U-2-OS cells transfected with the indicated siRNAs and expressing newly synthesised SNAP-H3.1 after HU exposure. See Figure 7C for schematic and average values. Scale bar = 10 μm. **(F)** Representative images of stable HeLa cells expressing WT H3.1-GFP or a K4A mutant (clone D1) from Figure 7D. **(G)** Stable HeLa cells from (F) were treated and analysed as in Figure 7D. Plots represent mean relative fluorescence intensities. Error bars indicate SD of values from n= 21-31 cells. Data from Figure 7D (WT and K4A (clone D1)) are presented for comparison. **(H)** Stable HeLa cells from (F) (WT and K4A clone D1) were transfected with the indicated siRNAs, exposed to MMC and analysed by FRAP as above (n = 10-22).

Figure S7, related to Figure 7: H3K4 methylation is required to promote fork protection through FANCD2-dependent histone H3 remodelling. (A) WCE from DT40 cells expressing the denoted GFP-tagged chFANCD2 variants were analysed by immunoblotting. **(B)** Representative FRAP images of stable U-2-OS-GFP-RAD51 cells from Figure 7F after exposure to MMC. Areas analysed are indicated with dashed boxes, and foci analysed by FRAP are denoted with arrows. Represented images are contrast enhanced for display purposes to make foci clearly visible. **(C)** U-2-OS cells were transfected with the indicated siRNAs, exposed to 50 ng/ml MMC for 24 h, and WCE analysed by immunoblotting. **(D)** HeLa nuclear cell extracts were incubated with the indicated immobilised histone peptides, and inputs and immunoprecipitates were analyzed by immunoblotting. **(E)** Purified FANCD2 (100 ng) was incubated with the indicated immobilised histone peptides, and inputs and immunoprecipitates were analyzed by immunoblotting. **(F)** Densitometric quantification of signals from (D-E). Immunoblots from two independent experiments were used. **(G)** Purified FANCD2 (100 ng) was incubated with recombinant histone H3 or an H3K4me1 EPL mimic, and inputs and immunoprecipitates were analyzed by immunoblotting. **(H)**

U-2-OS cells were transfected with the indicated siRNAs, treated as in (C), and immunostained with an antibody to FANCD2. Representative images are shown (*left*), and foci-positive cells were enumerated (*right*). **(I)** Quantification of PLA signals between EdU and FANCD2 in U-2-OS cells from (H). Where denoted, cells were exposed to 4 mM HU for 5 h.

SUPPLEMENTAL TABLE LEGEND

Table S1, related to Figures 3-7: Average values, SEM and p values for fork degradation experiments. The average tract length ratios (IdU:CldU) and SEM for all nascent strand degradation experiments are collated, along with the p values obtained for each condition relative to relevant control cells (denoted with '-'). ND = not determined.
